# Antioxidants: a comprehensive review

**DOI:** 10.1007/s00204-025-03997-2

**Published:** 2025-04-15

**Authors:** İlhami Gulcin

**Affiliations:** Faculty of Sciences, Department of Chemistry, Atatürk University, 25240 Erzurum, Türkiye

**Keywords:** Antioxidants, Antioxidant activity, Antioxidant methods, Oxidative stress, Phenolic compounds, Reactive oxygen species

## Abstract

Antioxidants had a growing interest owing to their protective roles in food and pharmaceutical products against oxidative deterioration and in the body and against oxidative stress-mediated pathological processes. Screening of antioxidant properties of plants and plant derived compounds requires appropriate methods, which address the mechanism of antioxidant activity and focus on the kinetics of the reactions including the antioxidants. Many studies have been conducted with evaluating antioxidant activity of various samples of research interest using by different methods in food and human health. These methods were classified methods described and discussed in this review. Methods based on inhibited autoxidation are the most suited for termination-enhancing antioxidants and, for chain-breaking antioxidants while different specific studies are needed for preventive antioxidants. For this purpose, the most commonly methods used in vitro determination of antioxidant capacity of food and pharmaceutical constituents are examined and also a selection of chemical testing methods is critically reviewed and highlighting. In addition, their advantages, disadvantages, limitations and usefulness were discussed and investigated for pure molecules and raw plant extracts. The effect and influence of the reaction medium on performance of antioxidants is also addressed. Hence, this overview provides a basis and rationale for developing standardized antioxidant capacity methods for the food, nutraceuticals, and dietary supplement industries. Also, the most important advantages and shortcomings of each method were detected and highlighted. The underlying chemical principles of these methods have been explained and thoroughly analyzed. The chemical principles of methods of 1,1-diphenyl-2-picrylhydrazyl (DPPH•) radical scavenging, 2,2'-azinobis-(3-ethylbenzothiazoline-6-sulphonate) radical (ABTS^·+^) scavenging, ferric ions (Fe^3+^) reducing assay, ferric reducing antioxidant power (FRAP) assay, cupric ions (Cu^2+^) reducing power assay (Cuprac), Folin–Ciocalteu reducing capacity (FCR assay), superoxide radical anion (O_2_^·−^), hydroxyl radical (OH·) scavenging, peroxyl radical (ROO·) removing, hydrogen peroxide (H_2_O_2_) decomposing, singlet oxygen (^1^O_2_) quenching assay, nitric oxide radical (NO·) scavenging assay and chemiluminescence assay are overviewed and critically discussed. Also, the general antioxidant aspects of the main food and pharmaceutical components were discussed through several methods currently used for detecting antioxidant properties of these components. This review consists of two main sections. The first section is devoted to the main components in food and their pharmaceutical applications. The second general section includes definitions of the main antioxidant methods commonly used for determining the antioxidant activity of components. In addition, some chemical, mechanistic, and kinetic properties, as well as technical details of the above mentioned methods, are provided. The general antioxidant aspects of main food components have been discussed through various methods currently used to detect the antioxidant properties of these components.

## Introduction

### Reactive oxygen species (ROS) and free radicals

Oxygen is a highly reactive nonmetal and a powerful oxidizing agent, easily forming oxides with most elements as well as with other compounds. In the atmosphere, it exists in its ground state as a stable triplet biradical (^3^O₂) and undergoes reduction in a stepwise manner (Taslimi and Gulçin, 2018; Rezai et al. 2018; Gulcin 2020). An oxygen-rich atmosphere enabled the emergence of aerobic organisms and various energy generation systems that use O_2_ as the final electron acceptor. Although molecular oxygen is relatively stable, its reduction leads to the formation of reactive oxygen species (ROS), which are naturally and continuously produced as a byproduct of aerobic cellular metabolism. From this perspective, approximately 1–2% of the total O_2_ used or consumed by metabolism is converted into ROS (Soares et al. 2019; Izol et al. 2024). In this ground state, molecular oxygen contains two unpaired electrons with parallel spins in separate anti-bonding orbitals, which limits its reactivity by restricting direct electron pairing. As a result, molecular oxygen can accept paired electrons from an electron donor. Meanwhile, redox reactions play a crucial role in metabolism within living systems, where electrons transfer from one molecule to another. These processes are fundamental in biological systems, setting off a chain of reactions in organisms that utilize oxygen from the air for oxidation, producing energy in the form of ATP (Gulcin 2012; Zengin et al. 2025a, b). Oxygen is deeply integrated into a variety of oxidation–reduction and enzymatic processes within living organisms. Its ability to electron transfer between atoms makes it crucial for aerobic life and cellular metabolism, as oxygen serves as the final electron acceptor in the electron transport chain, ultimately generating energy in the form of ATP (Davies 1995; Gülçin et al. 2005a, b; Elmastas et al. 2018; Kızıltaş et al., 2025). However, complications can arise if electron flow becomes uncoupled, leading to the generation of free radicals.

Free radicals are atoms, molecules, or ions with unpaired electrons, making them highly unstable and reactive in chemical interactions with other molecules. They originate from three elements: oxygen, nitrogen, and sulfur, giving rise to reactive oxygen species (ROS), reactive nitrogen species (RNS), and reactive sulfur species (RSS) (Carocho et al. 2013; Aslan et al. 2024). Free radicals were first described by Moses Gomberg over a century ago (1900). For a long time, they were thought to be absent in biological systems due to their high reactivity and short lifespans. While this assertion was generally incorrect, it sparked interest in the role of free radicals in biological processes. In the 1950s, free radicals were discovered in biological systems (Commoner et al. 1954; Gülçin et al. 2025), and it was immediately hypothesized that they played a role in various pathological processes (Gerschman et al. 1954) and aging (Harman 1956, 2009). Since then, our understanding of free radicals’ involvement in living processes has expanded significantly. For a long time, they were primarily considered harmful due to their potential for causing damage. This view was reinforced by the discovery of McCord and Fridovich (1969), who identified the first protective enzyme against free radicals, superoxide dismutase (SOD). From the 1970s to the 1990s, the idea of free radicals as solely damaging agents in biological systems was challenged by several key discoveries. First, it was found that free radicals were responsible for fighting infections through the immune system (Babior et al. 1973, 1975; Rosi et al. 1985; Britigan et al. 1987). Second, in the 1980s, vascular endothelial cells were shown to produce nitric oxide from l-arginine, which explained the biological effects attributed to endothelium-derived relaxing factor (Furchgott and Zawadzki 1980; Palmer et al. 1987; Furchgott and Vanhoutte 1989). This discovery opened a new avenue for free radical research, highlighting their role in signaling, initially for nitric oxide and later for other reactive species (Scandalios 2005). Finally, it was discovered that the levels of free radicals are regulated by hormones like insulin (Spagnoli et al. 1995), and they were proposed to regulate key metabolic pathways (Shaikhali 2008). ROS have a dual role in plant cells, depending on their concentration. At low levels, they function as intracellular signaling molecules, triggering a positive response in the antioxidant system. However, at high concentrations, all forms of ROS become toxic and can interact with various organic molecules, such as nucleic acids and lipids (Sharma et al. 2012; Nilofar et al. 2024). As a result, oxidative stress occurs when there is an imbalance between ROS production and elimination, representing a complex biochemical and physiological phenomenon (Soares et al. 2019; Zengin et al. 2025b) Therefore, it is now clear that free radicals are active participants in various processes and should no longer be considered merely as damaging agents, but rather as key players in many normal functions of living organisms (Güven et al. 2024a; b). In order to understand the biological effects of ROS, we need to know and determine what species are produced in different circumstances and their reactivities. Free radicals are generated internally as a natural byproduct of metabolism in mitochondria, as well as through xanthine oxidase activity, peroxisomes, inflammatory processes, phagocytosis, arachidonate pathways, ischemia, and physical exercise. External factors that contribute to the production of free radicals include smoking, environmental pollutants, radiation, drugs, pesticides, industrial solvents, and ozone (Güven et al. 2023a; Kınalıoğlu et al. 2023). It is paradoxical that these elements particularly oxygen, essential for life, can exert harmful effects on the human body via these reactive species. The problem here is not the production of free radicals, but rather their abnormal and excessive production under conditions such as stress. Researches on free radicals, more commonly referred to as reactive species, within biological systems represents one of the most dynamic fields; however, it is also among the most complex for several reasons (Halliwell and Gutteridge 1989; Ozden et al. 2023). The primary challenges can be listed as follows.

- Their low stability and high reactivity.
- The broad diversity of reactions in which they can engage.
- The complex spatiotemporal distribution within cellular and extracellular spaces.
- Their dependency on the physiological state of the organism.
- The lack of reliable technical tools for accurately assessing their absolute or even relative levels.

Due to these inherent challenges in studying free radicals, the involvement of researchers from diverse fields, and the universal significance of reactive species-related processes, this review seeks to clarify and define key terms, describe the concept of oxidative stress, and provide general classifications of this type of stress (Lushcak et al. 2014; Karageçili et al. 2023a).

**Fig. 1:**
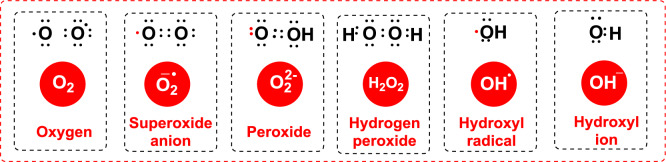
Structure of common reactive oxygen species (ROS)

Free radicals are highly reactive and prone to initiating chemical reactions with other molecules. They primarily stem from oxygen, nitrogen, and sulfur. For instance, oxygen-based free radicals, known as reactive oxygen species (ROS), include species like hydroxyl (HO·), superoxide (O_2_^·−^), peroxyl (ROO·), alkoxyl (RO·), and nitric oxide (NO·) radicals, which differing for half-life, reactivity, and localization in the cell (Durmaz et al. 2023a). Among these, the hydroxyl radical, with a half-life of 10^–9^ s, and the alkoxyl radical, with a half-life of several seconds, are especially reactive, rapidly attacking molecules in nearby cells. The O_2_^·−^ has a very short lifespan in biological systems due to its high reactivity. Under normal conditions, its lifespan ranges from a few microseconds to a few milliseconds (Gülçin 2020; Kızıltaş et al. 2023). ROS generation is tightly regulated by redox signaling and sensing mechanisms (Fig. 1).

**Fig. 2:**
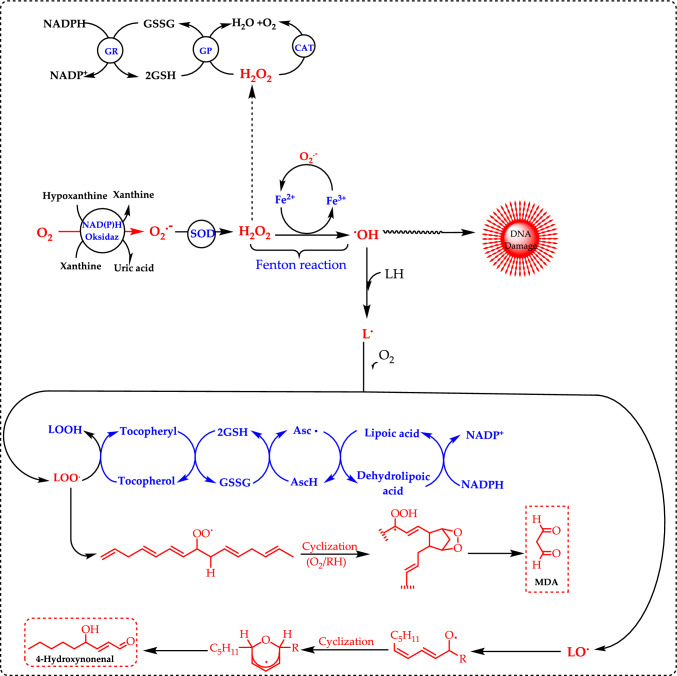
A summary of the reactions that result in the generation of reactive oxygen species (ROS) and their impacts. (*SOD* superoxide dismutase enzyme, *CAT* catalase enzyme, *GR* Glutathione reductase, *GP* Glutathione peroxidase)

It is mostly inactivated by being rapidly converted to water and oxygen by the enzyme superoxide dismutase (SOD). In this way, it protects cells from oxidative stress. The damage caused by free radicals is often inevitable and typically managed by cellular repair mechanisms. In contrast, species like O_2_^·−^, NO·, and lipid hydroperoxides tend to be less reactive (Ames et al. 1993; Han et al. 2018; Bulut et al. 2018). In addition to these ROS in living organisms there are other ROS nonradical forms, such as the singlet oxygen (^1^O_2_), hydrogen peroxide (H_2_O_2_), and hypochlorous acid (HOCl) (Pietta 2000; Durmaz et al. 2023a). Regarding ROS, the reactions leading to the production of reactive species are displayed in Fig. 2. Also, a summary of the putative ROS, RNS, and non-free radical species is presented in Table 1. The term ROS is typically used to refer to the initial reactive species formed during oxygen reduction and their secondary reactive products, while RNS is a commonly used term to describe reactive species derived from nitric oxide (Karageçili et al. 2023c).

**Table 1:** Reactive oxygen species (ROS) and reactive nitrogen species (RNS)

Reactive oxygen species		Non free-radical species	
• Hydroxyl radical	HO·	• Hydrogen peroxide	H_2_O_2_
• Superoxide radical	O_2_·^­^	• Singlet oxygen	^1^O_2_
• Hydroperoxyl radical	HOO·	• Ozone	O_3_
• Lipid radical	L·	• Lipid hydroperoxide	LOOH
• Lipid peroxyl radical	LOO·	• Hypochlorous acid	HOCl
• Peroxyl radical	ROO·	• Peroxynitrite	ONOO^−^
• Lipid alkoxyl radical	LO·	• Dinitrogen trioxide	N_2_O_3_
• Nitrogen dioxide radical	NO_2_·	• Nitrous acid	HNO_2_
• Nitric oxide radical	NO·	• Nitryl chloride	NO_2_Cl
• Nitrosyl cation	NO^+^	• Nitroxyl anion	NO^−^
• Thiyl radical	RS·	• Peroxynitrous acid	ONOOH
• Protein radical	P·	• Nitrous oxide	N_2_O

A free radical, often referred to as ROS or RNS, is an unstable entity that possesses one or more unpaired electrons in its outermost orbital. This instability arises from its failure to achieve an octet configuration. ROS and RNS exhibit several characteristics, including the ability to produce additional vulnerable ROS, a brief lifespan, and the potential to cause damage to various tissues. The process of sequentially reducing molecular oxygen to water occurs in four univalent steps, as outlined by Gulcin in 2012 and 2020.

$${\text{O}}_{2}\stackrel{{\text{e}}^{-}}{\to } {\text{O}}_{2}^{\cdot -}\stackrel{{\text{e}}^{-}}{\to }{\text{O}}_{2}^{2-}\stackrel{{\text{e}}^{-}}{\to }\text{ OH}\cdot \stackrel{{\text{e}}^{-}}{\to } {\text{H}}_{2}\text{O}$$

In this electron flow, O_2_ can be reduced through various pathways, including the generation of O_2_·^−^, the formation ^1^O_2_, or the production of H_2_O_2_. Most of the oxygen utilized by the body is completely reduced to water in a single step at the terminal cytochrome of the mitochondrial oxygen transport chain. However, under certain conditions, reduced oxygen may transition through intermediate forms before ultimately producing H_2_O_2_. This compound can be broken down into water and oxygen through the action of catalase (CAT) and various peroxidase (POD) enzymes (Gülçin et al. 2009; Apak et al. 2022). This process is facilitated by intracellular SOD, which serves as a protective mechanism against superoxide toxicity and is found throughout the body. On the contrary, there are also enzymes such as polyphenoloxidase (PPO) that induce oxidation in metabolism (Gülçin, Küfrevioğlu and Oktay 2005; Gülçin and Yıldırım, 2005; Köksal and Gülçin 2008). The damage caused by ROS arises from the partial reduction of oxygen. Notably, most ROS are generated at low levels during normal aerobic metabolism and play crucial roles in the redox-dependent regulation of various signaling pathways in living organisms (Shivakumar and Kumar 2018; Çakmakçı et al. 2023). ROS are continuously generated by the body’s normal utilization of oxygen, including processes like respiration and certain immune functions (Gulcin 2006a; Atalar et al. 2023). A free radical is a chemical species capable of existing independently and characterized by the presence of one or more unpaired electrons. These free radicals are highly unstable molecules produced as a natural outcome of metabolic activities within the mitochondria, as well as through pathways involving xanthine oxidase, peroxisomes, phagocytosis, inflammatory processes, ischemia, arachidonic acid metabolism, and even physical exercise. In contrast, various external factors contribute to the increased production of free radicals, including environmental pollutants, smoking, radiation, pharmaceuticals, ozone, pesticides, and industrial solvents. Ironically, while these elements are vital for life, they can also have harmful effects on the human body through the generation of these reactive species (Lobo et al. 2010; Güven et al. 2023b). Free radicals engage in various reaction mechanisms, interacting with surrounding molecules through electron donation or acceptance, reducing or oxidizing radicals, as well as hydrogen abstraction, self-annihilation reactions, addition reactions, and disproportionation (Slater 1984; Carocho and Ferreira 2013).

- $${\text{OH}}^{\cdot }+ {\text{RS}}^{-} \to {\text{OH}}^{-}+ {\text{RS}}^{\cdot }$$
- $${\text{CCl}}_{3}^{\cdot }+\text{RH} \to {\text{CHCl}}_{3}+{\text{R}}^{\cdot }$$
- $${\text{CCl}}_{3}^{\cdot }+{\text{CCl}}_{3}^{\cdot } \to {{\text{CH}}_{2}\text{Cl}}_{6}$$
- $${\text{CCl}}_{3}^{\cdot }+{\text{CH}}_{2}={\text{CH}}_{2} \to {{\text{CH}}_{2}(\text{CCl}}_{3}) -{\text{CH}}_{2}$$
- $${\text{CH}}_{3}{\text{CH}}_{2}^{\cdot }+{\text{CH}}_{3}{\text{CH}}_{2}^{\cdot } \to {\text{CH}}_{2}={\text{CH}}_{2}+ {\text{CH}}_{3}-{\text{CH}}_{3}$$

The term of reactive oxygen species (ROS) is an umbrella term for an array of molecular oxygen derivatives, which occur as a normal attribute of aerobic life. ROS are characterized as either radicals with at least one unpaired electron or reactive nonradical compounds that can oxidize biomolecules. Also, elevated formation of the different ROS leads to molecular damage, denoted as ‘oxidative distress’ (Sies and Jones 2020; Karageçili et al. 2023b). These intermediates are often referred to as oxidants or prooxidants (Sies 1991; Koksal et al. 2017a). ROS are produced continuously during normal physiological processes and have the potential to initiate lipid peroxidation, resulting in the accumulation of lipid peroxides (Gulçin 2010; Ekinci Akdemir et al. 2017; Kandemir et al. 2017a). At physiological levels, ROS are essential for various normal cellular functions. However, when produced in excess, they can lead to oxidative damage over time, disrupting cellular functions and ultimately causing cell death by harming DNA, RNA, lipids, and proteins (Isik et al. 2017; Krawczyk 2019). They can also inflict damage on vital biomolecules, including nucleic acids, lipids, proteins, polyunsaturated fatty acids, and carbohydrates (Tohma et al. 2017; Koksal et al. 2017b; Mutlu et al. 2023a). This damage may result in mutations due to DNA damage. If the cellular mechanisms to scavenge ROS are inadequate, they can trigger free radical chain reactions that further damage proteins, lipids, and nucleic acids, potentially leading to various disease conditions (Craft et al. 2012; Schieber and Chandel 2014). ROS have significant destructive effects on mitochondria, which are known as the cell's powerhouse due to their role in producing ATP through oxidative phosphorylation in the electron transport chain. Mitochondrial dysfunction can lead to increased oxidative stress and ROS production, resulting from various factors (Gulcin et al. 2004a; Krawczyk 2019). To protect against these damaging effects, living organisms, including humans, utilize both endogenous and exogenous antioxidant compounds that scavenge free radicals (Hamad et al. 2017; Anraku et al. 2018). The human body possesses a sophisticated array of natural enzymatic and non-enzymatic antioxidant defenses, which help mitigate the harmful impact of free radicals, known to be linked to numerous diseases and other oxidizing agents (Ekinci Akdemir et al. 2016a, 2016b). By scavenging ROS, the human body can lower the risk of developing these conditions, thereby enhancing overall quality of life (Gülçin 2012; Aksu et al. 2016; Yılmaz et al. 2023). Moreover, a sufficient intake of dietary antioxidants can further bolster protection against free radicals (Alam et al. 2013a, b; Ekinci Akdemir et al. 2016a).

ROS are produced in the body as part of the primary immune defense mechanism. Phagocytic cells, including neutrophils, monocytes, and macrophages, generate significant amounts of O_2_·^−^ and NO· to combat foreign invaders. However, excessive activation of these phagocytes in certain diseases can lead to tissue damage primarily due to ROS activity (Diplock et al. 1998; Bae et al. 2016; Tohma et al. 2016). Moreover, ROS can cause oxidative damage to various biomolecules, including lipids, nucleic acids, proteins, and carbohydrates, contributing to aging, cancer, and numerous other health issues (Aruoma 1994; Kiziltas et al. 2022a). Also, ROS have been linked to over 100 diseases, such as cardiovascular disease, diabetes, malaria, acquired immunodeficiency syndrome, heart disease, stroke, arteriosclerosis, and cancer (Tanizawa et al. 1992; Duh 1998).

The exposure to environmental factors such as smoking, ultraviolet radiation (UV), heavy metals, ozone, allergens, drugs, toxins, and environmental pollutants can elevate ROS levels within cells. Ionizing radiation contributes to this process by breaking down water molecules, which are integral to cellular structures. During radiolysis, these water molecules are split into hydrogen atoms and hydroxyl radicals (Kiziltas et al. 2022b; Bayrak et al. 2022). Subsequently, interactions between the radiolysis products can result in the formation of hydrogen peroxide. Studies have shown that ionizing radiation exposure in fibroblast cells leads to an increase in ROS production. Notably, modern radiotherapy approaches leverage the radiation-induced generation of ROS within cancer cells as a strategy for enhanced cancer treatment outcomes (Mucha et al. 2021; Durmaz et al. 2022a). UV radiation also plays a role in ROS production, notably by activating NADPH oxidase. When UV radiation is absorbed, it triggers ionization and the breakdown of molecules, ultimately resulting in the formation of free radicals. Among the effects of UV radiation is ozone generation, which can impair lung function and induce inflammation in the respiratory epithelium (Antunes et al. 2018; Topal and Gulçin 2022).

The biological pathways responsible for the endogenous production of ROS exemplify a broader class of reactive intermediates and their generation mechanisms. Free radicals are generated internally as a natural part of metabolism in mitochondria, as well as through xanthine oxidase activity, peroxisomes, inflammation processes, phagocytosis, arachidonic acid pathways, ischemia, and physical exercise (Durmaz et al. 2022b; Gulçin et al. 2022). In living beings, various forms of ROS can arise through multiple pathways. External factors contributing to free radical production include normal aerobic respiration activates, smoking, environmental pollutants, polymorphonuclear leukocytes, macrophages, radiation, certain medications, pesticides, industrial solvents, and ozone. It is somewhat paradoxical that elements essential for life (notably oxygen) can harm the human body through these reactive species (Lobo et al. 2010; Polat Köse and Gulçin 2021). It is also important to recognize that organisms are exposed to ROS from external sources. On the other hand, the external sources of ROS encompass tobacco smoke, organic solvents, specific pollutants, and pesticides (Halliwell and Gutteridge 1989; Topal et al. 2016a). Additionally, certain pro-oxidant compounds, such as quinones that participate in redox cycling, are ingested through diet. Furthermore, cigarette smoke introduces a range of radicals, and ozone—an ROS whose levels are rising due to air pollution—can oxidize lipids (Diplock et al. 1998; Polat Köse et al. 2015). While numerous sources of specific ROS exist within the human body, the O_2_·^−^ is particularly significant as it initiates reaction sequences leading to the formation of other reactive intermediates. It is estimated that about 1–2% of the oxygen we utilize is converted into ROS including O_2_^·−^ (Halliwell 1996; Gulcin 2012). Biological systems are constantly exposed to reactive oxidants from external sources as well as those generated internally, which can be seen as a trade-off for the benefits of oxygen metabolism. Mitochondrial respiration serves as a primary source of ROS. Additionally, they are produced by ionizing and UV radiation, as well as through the metabolism of various drugs and xenobiotics (Winterbourn 2008; Kızıltaş et al. 2021a, b). ROS can be detailed as follows.

**Superoxide anion radicals (O**_**2**_^**.−**^**)** are typically the first ROS produced, and its formation is primarily linked to electron transport chains. O_2_^·−^ is a type of ROS that forms when molecular oxygen (O_2_) gains a single electron. It is a relatively unstable and highly reactive molecule that plays a dual role in biological systems, acting as both a signaling molecule and a source of oxidative stress in moderate levels (Kızıltaş et al. 2021a). However, in excess, it can cause oxidative damage to lipids, proteins, and DNA, contributing to aging, inflammation, and diseases such as cancer and neurodegenerative disorders. It is generated in cells during various metabolic processes, primarily as a byproduct of the electron transport chain in mitochondria. Cells counteract superoxide's harmful effects through antioxidant defense systems, including SOD, which catalyzes its conversion into H_2_O_2_, which is then further detoxified by catalase or peroxidases. Enzymes like NADPH oxidase can also produce superoxide during immune responses. The main sources of O_2_^·−^ within plant cells are mitochondria and chloroplasts, specifically in complexes I and III, and PS I and PS II, respectively (Noctor et al. 2006; Sharma et al. 2012). However, its production can also occur in other organelles, such as peroxisomes, glyoxysomes, and even in the cell wall (Gill and Tuteja 2010). It is less reactive than some other ROS (e.g., hydroxyl radicals), but it can react with other molecules to form more harmful species, such as H_2_O_2_ or peroxynitrite (ONOO^−^). Compared to other ROS, O_2_^·−^ is considered a moderately reactive radical with a short half-life and low mobility due to its negative charge, which limits its ability to cross biological membranes (Demidchik 2015). The O_2_^·−^ cannot directly interact with organic macromolecules, and its toxicity arises from its strong reducing capacity, which can convert Fe^3+^ to Fe^2+^, allowing it to later react with H_2_O_2_ and generate OH·, one of the most toxic ROS (Ahmad et al. 2014; Demidchik 2015; Mittler 2017). This process is widely known as the Haber–Weiss reaction, and its final step, where Fe^2+^ interacts with H_2_O_2_, is referred to as Fenton's reaction (Cuypers et al., 2016). Additionally, O_2_^·−^ can undergo protonation, leading to the formation of HO_2_^.−^, a more reactive and stable molecule that can pass through biological membranes (Bielski, Arudi and Sutherland, 1983).

**Hydroxyl radicals (OH·)** is the most dangerous and primarily formed through the Fenton reaction, where H_2_O_2_ reacts with ferrous iron (Fe^2+^).

$$\frac{\begin{gathered} {\text{Fe}}^{2 + } + {\text{H}}_{2} {\text{O}}_{{2{ }}} \to {\text{Fe}}^{3 + } + {\text{OH}}^{ - } + {\text{OH}}^{ \bullet } \left( {\text{Fenton reaction}} \right) \hfill \\ {\text{O}}_{2}^{ \bullet - } + {\text{H}}_{2} {\text{O}}_{{2{ }}} + {\text{Fe}}^{3 + } \to {\text{O}}_{2} + {\text{Fe}}^{2 + } + {\text{OH}}^{ - } + {\text{OH}}^{ \bullet } \left( {{\text{Haber}}{-}{\text{Weiss reaction}}} \right) \hfill \\ \end{gathered} }{{{\text{O}}_{2}^{ \bullet - } + {\text{H}}_{2} {\text{O}}_{{2{ }}} \to {\text{O}}_{2} + {\text{H}}_{2} {\text{O}} + {\text{OH}}^{ \bullet } \left( {\text{Overall reaction}} \right)}}$$

**Fig. 3:**
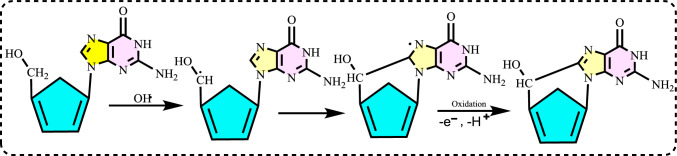
Reaction of hydroxyl radical with the nükleozite moiety of DNA

It is also produced via the Haber–Weiss reaction, which involves the interaction of O_2_^·−^ and H_2_O_2_.O_2_^·−^ is the neutral counterparts of hydroxide ions (OH −) and are known for their high reactivity, leading to the formation of OH − groups and a very short lifespan. The hydroxyl radical is highly reactive because it has an unpaired electron. It is non-selective in its reactivity, meaning it attacks any nearby biomolecule, resulting in extensive damage to cellular structures (Cooke et al. 2003). Research indicates that a human cell is subjected to OH· and other reactive species approximately 100,000 times daily, contributing to oxidative stress (Valko et al. 2004; Carocho and Ferreira 2013). The main targets of free radicals are proteins, DNA, RNA, sugars and lipids. Nucleosides in DNA can be given as examples of how ROS attack biomolecules. As illustrated in Fig. 3, the OH· reacts with the sugar moiety of DNA by abstracting a hydrogen atom from the carbon atom. A unique reaction involving the C5'-centered radical of the sugar moiety in DNA is its addition to the C8 position of the purine ring within the same nucleoside like guanine. This intramolecular cyclization leads to the formation of 8,5'-cyclopurine-2'-deoxynucleosides. The reactions of carbon-centered sugar radicals result in DNA strand breaks and the formation of base-free sites through various mechanisms.

These radicals can be produced as byproducts of the immune response, primarily generated by microglia and macrophages when they encounter specific pathogens and certain bacteria. The damaging effects of OH· are associated with various neurological autoimmune conditions, particularly when immune cells become overly activated and harm surrounding healthy cells (Gulcin 2012). Hydroxyl radical has a very short half-time of around 1 ns (Mittler 2017). Recognized as the most reactive species, OH· have an estimated half-life of about 10^–9^ s. Its major targets and sites of action are therefore located in close proximity to its production site (Sharma et al. 2012). Due to its chemical properties, which result in high reactivity and consequently high toxicity, the ·OH can inflict severe damage on all organic molecules despite its extremely short lifespan. These potential damages are further amplified by the absence of any enzymatic mechanism dedicated to its degradation and metabolism. Unsurprisingly, elevated levels of ·OH are associated with programmed cell death (Gill and Tuteja 2010; Sharma et al. 2012; Demidchik 2015). They can be generated in vivo through high-energy irradiation, which causes hemolytic cleavage of water, or through metal-catalyzed processes involving endogenous H_2_O_2_. While UV light lacks the energy to directly split water, it can decompose H_2_O_2_, resulting in the production of two OH·. Due to their high reactivity, OH·tends to react immediately at their site of generation (Diplock et al. 1998).

**Nitric oxide (NO·)** is a crucial signaling molecule in the body, playing vital roles in various physiological and pathological processes. It is an enzymatically generated signaling molecule derived from arginine that plays a crucial role in relaxing smooth muscle within blood vessel walls, leading to reduced blood pressure. As a vital cellular messenger, NO· is involved in various physiological and pathological processes. It is also produced by activated macrophages, where it contributes to the body's primary immune defense. In the body, NO is primarily synthesized by the enzyme nitric oxide synthase (NOS), which converts L-arginine into NO and L-citrulline in the presence of oxygen and cofactors like NADPH and tetrahydrobiopterin. NOS contains three types include endothelial NOS (eNOS), which regulates blood vessel dilation and cardiovascular function, neuronal NOS (nNOS),which involved in neurotransmission and brain function and inducible NOS (iNOS) that produced during immune responses, helping to kill pathogens.

$$\text{L}-\text{Arginin}+ {\text{O}}_{2}\stackrel{\text{NOS}}{\to } \text{L}-\text{Citruline}+\text{NO}$$

NO is a vasodilator, meaning it relaxes blood vessels, improving blood flow and lowering blood pressure. It is produced by the endothelial cells (cells lining blood vessels) by nitric oxide synthase enzymes. White blood cells (macrophages) produce NO to kill bacteria and viruses. (Moncada et al. 1991). Also, NO acts as a neurotransmitter in the brain, involved in memory, learning, and neuroprotection. NO improves oxygen and nutrient delivery to muscles, enhancing endurance and recovery (Murad 2004). However, excessive levels of NO· can be cytotoxic, as it may react directly with biomolecules or combine with O_2_^·−^ to form peroxynitrite (ONOO-) (Hou et al. 1999). NO is a vital signaling molecule involved in vascular function, immune response, and neural activity. Maintaining balanced NO levels is essential for overall health and disease prevention (van Faassen et al. 2009).

**Peroxynitrite (ONOO**^**−**^**)** is a powerful oxidant that can cause damage to cells, lipids, proteins, and DNA, making it an important factor in oxidative stress and various diseases. It is known to induce lipid peroxidation in lipoproteins and can also disrupt cellular signaling by nitrating tyrosine residues in proteins (Packer 1996). NO is beneficial in normal conditions, but when it reacts with superoxide (O₂^•^⁻), it produces peroxynitrite, which can be harmful. As both an oxidant and a nitrating agent, peroxynitrite's oxidizing properties enable it to inflict damage on various cellular molecules, including proteins and DNA (Szabo et al. 2007). Peroxynitrite is formed in biological systems through the following reaction:

$${\text{NO}} + {\text{ O}}_{2}^{ \bullet - } \to {\text{ONOO}}^{ - }$$

The formation of ONOO^−^ from the reaction of NO with O_2_^•−^ represents a crucial factor in understanding the dual roles of NO in health and disease. Moreover, ONOO − is a potent oxidant that can directly interact with electron-rich groups, such as sulfhydryls, zinc-thiolates, iron-sulfur centers, and the active site sulfhydryl groups in tyrosine phosphatases (Pacher et al. 2007). It reacts with lipids, causing lipid peroxidation and damaging cell membranes. It modifies proteins by nitrating tyrosine residues, altering protein function. NO can damages DNA, which leading to mutations and cell death (Beckman and Koppenol 1996).

**Peroxyl radical (ROO·)** is known for its relatively long lifespan and significant diffusion distance within biological systems. ROO**·** is another highly ROS that play a crucial role in oxidative stress, lipid peroxidation, and various biological processes. They are often formed during the oxidation of lipids, proteins, and DNA, contributing to cellular damage and disease progression. Peroxyl radicals are mainly formed through the reaction of organic radicals (R•) with molecular oxygen (O_2_):

$${\text{R}}^{ \bullet } + {\text{ O}}_{2} \to {\text{ROO}}^{\cdot}$$

This process commonly occurs in lipid peroxidation damaging cell membranes and leading to loss of function (Yin et al. 2011). Peroxyl radical leads to enzyme inactivation and protein aggregation, linked to neurodegenerative like Alzheimer’s and Parkinson’s diseases. It can be produced during lipid peroxidation, a process that begins with the abstraction of a hydrogen atom from polyunsaturated fatty acids. OH· can initiate this chain reaction (Reaven and Witzum, 1996). As lipid peroxidation progresses, it generates additional products, including RO· and ROOH. These hydroperoxides may rearrange into endoperoxide intermediates, which can then be cleaved to form aldehydes. The interaction of these aldehydes with amine groups in proteins has been proposed as a potential mechanism for modifying the protein components of lipoproteins (Diplock et al. 1998). Peroxyl radicals (ROO•) are key players in oxidative damage, affecting lipids, proteins, and DNA. The body uses antioxidant defenses to minimize their harmful effects, but imbalances in oxidative stress contribute to various chronic diseases and aging (Davies 2016).

**Hydrogen peroxide (H**_**2**_**O**_**2**_**)** is the simplest peroxide, characterized by its oxygen–oxygen single bond. H_2_O_2_ and O_2_^·−^ are regarded as primary ROS, but the former can induce more severe oxidative stress due to its greater stability compared to O_2_^•−^ (Sharma et al. 2012). H_2_O_2_ production is closely linked to electron transport chain in the various organelles including mitochondria, chloroplasts, endoplasmic reticulum, and plasma membrane, as well as to photorespiration metabolism and the β-oxidation of fatty acids (Sharma et al. 2012; Mittler 2017). It is inherently unstable and decomposes gradually when exposed to light. H_2_O_2_ occurs naturally in biological systems, including the human body, where enzymes known as peroxidases play a role in its utilization and decomposition. Formed through the two-electron reduction of O_2_, H_2_O_2_ is not a free radical but acts as an oxidizing agent. In the presence of oxygen and transition metal ions, H_2_O_2_ can produce OH· via the Fenton reaction, as described by Halliwell and Gutteridge (1989, 2015). Additionally, the Haber–Weiss reaction generates OH· from H_2_O_2_ and O_2_^•−^, with Fe^2+^ acting as catalysts. Superoxide reduces Fe^3^⁺ back to Fe^2^⁺, allowing the cycle to continue. This reaction was first introduced by Fritz Haber and his student Joseph Joshua Weiss in 1932. Subsequent research has established that the Haber–Weiss and Fenton reactions are significant contributors to the generation of radicals, which can lead to oxidative stress and cellular damage. This reaction is significant in biological systems because the OH• it produces is highly reactive and can damage cellular components such as lipids, DNA, proteins and contributing to aging and various diseases. The high toxicity of H_2_O_2_ can be easily explained by its chemical nature: it has no unpaired electrons and possesses a relatively long half-time (1 ms), so it is able to cross biological membranes and to diffuse across long distances, increasing the number of potential sites of action (Gupta et al. 2015). Superoxide can reduce Fe^3^⁺ back to Fe^2^⁺ in biological and chemical systems. This reduction helps to regenerate Fe^2^⁺, allowing cycles like the Fenton reaction to continue. In the Fenton reaction, Fe^2^⁺ reacts with H₂O₂ to produce •OH, which are highly reactive and can cause oxidative damage in cells. However, once Fe^2^⁺ is oxidized to Fe^3^⁺, O₂^•^⁻ can reduce it back to Fe^2^⁺, sustaining the cycle and contributing to ongoing ROS production (Gülçin 2020).

The short-lived OH· radical, once formed, attacks biomolecules non-specifically in a diffusion-limited reaction, allowing it to degrade proteins, polysaccharides, and nucleic acids within just a few nanometers of its generation site (Shivakumar and Kumar 2018). In contrast, H₂O₂, a substrate for the Fenton reaction, is naturally produced as a by-product of oxidative metabolism in organisms. It is a non-radical reactive species that can easily diffuse between cells. The catalase enzyme efficiently converts H₂O₂ into water, a process that regulates its half-life. Evidence suggests that H₂O₂ is also involved in signal transduction, regulating gene expression via pathways like nuclear factor and apoprotein (Sen and Packer 1996).

**Singlet oxygen (**^**1**^**O**_**2**_**)** is a high-energy, reactive form of molecular oxygen (O_2_) in which both electrons in the highest occupied molecular orbital are paired with opposite spins. This makes it more reactive than the normal triplet oxygen (^3^O₂), which has two unpaired electrons with parallel spins (Foote 2001; Atkins et al. 2018). ^1^O₂ is highly reactive reagent and can oxidize biomolecules including lipids, proteins and DNA. It has a short lifetime in solution (µs in water, longer in organic solvents). It plays a significant role in photodynamic therapy for cancer treatment (George and Abrahamse 2022). The generation of singlet oxygen (^1^O_2_) is associated with the energy transfer from the triplet state of chlorophyll to O_2_, which occurs under intense light conditions and/or low CO_2_ assimilation rates. Under these conditions, this ROS can cause damage to photosystems I and II (Gill and Tuteja 2010; Soares et al. 2019). It is not a radical, but it is a highly ROS known to cause skin damage from UV exposure and to exert cytotoxic effects in tumor cells during photodynamic therapy. This ROS plays a role in skin photoaging and in triggering cell death in cancer cells as part of photodynamic treatments. Despite its importance, the biological effects of ^1^O₂ are still not fully understood (Homma et al. 2019). Singlet oxygen is the name for the diamagnetic form of O₂, which is less stable than the usual triplet oxygen. It is another non-radical ROS believed to form in light-exposed tissues in the body. Its half-life is estimated to be around 10⁻⁶ seconds, depending on the properties of the surrounding matrix. Singlet oxygen can react with various cellular components, showing a strong preference for reacting with conjugated double bonds, which makes PUFAs or guanine bases in DNA primary targets in cells (Di Mascio et al. 2019). It can also interact with other molecules through energy transfer or by forming chemical bonds (Stahl and Sies 1993). Chemically, ^1^O₂ is a highly reactive species with a short lifespan of 4–100 μs. It can interact with various biological molecules, leading to lipid peroxidation and the oxidation of other biomolecules including proteins, nucleic acids and fatty acids (Mittler 2017; Singh et al. 2018). Some cellular metabolites, such as β-carotene, tocopherol, and plastoquinone can neutralize ^1^O_2_. When present in excess, ^1^O₂ also induces the upregulation of several defense-related genes (Soares et al. 2019).

In a typical cell, there exists a delicate balance between prooxidants and antioxidants. This equilibrium, however, can be disrupted in favor of prooxidants when there is a substantial increase in ROS production or a significant reduction in antioxidant levels. This disrupted state is known as oxidative stress. Sies (1997) originally defined oxidative stress as a breakdown in the prooxidant-antioxidant balance. Another definition describes oxidative stress as an imbalance between the formation of ROS and a biological system’s capacity to neutralize these reactive intermediates or repair the damage they cause (Kalin et al. 2015; Oztaskin et al. 2015). Oxidative stress generally arises through two main mechanisms. Firstly, antioxidant levels may be diminished due to genetic mutations affecting antioxidant enzymes, exposure to toxins, or inadequate intake of natural antioxidants. Secondly, the number of reactive oxygen, nitrogen, or carbon species generated by activated immune cells increases under chronic inflammation (Somogyi et al. 2007; Gulcin 2012).

All aerobic organisms possess antioxidant defense systems, including antioxidant enzymes and antioxidant components that work to eliminate or repair damaged molecules. Similar to chemical antioxidants, cells are shielded from oxidative stress through an interconnected network of antioxidant enzymes (Davies 1995). Moreover, enzymes have been investigated as a novel category of natural antioxidants in certain food applications. They can be effectively utilized to remove oxygen and ROS and to decrease lipid hydroperoxides (Frankel 1998; Isik et al. 2015).

### Oxidative stress

Oxidative stress, a concept introduced by Sies in 1985, arises when the endogenous antioxidant defense system of cells and tissues is unable to cope with a sudden or prolonged increase in ROS production, leading to the disruption of redox signaling and regulation, as well as molecular damage (Sies 1985, 2015; Bingöl et al. 2021). Oxidative stress is a relatively recent concept that has been widely utilized in medical sciences over the past 3 decades. Researchers generally refer to oxidative stress when one or more parameters reflecting the balance of free radical processes are disturbed, leading to an increase in the steady-state level of ROS that affects many vital processes. In most cases, it is known that several parameters which include levels of at least couple oxidatively modified cellular components and activities of antioxidant and related enzymes are used as markers of oxidative stress (Lushchak 2014; Kızıltaş et al. 2021b). The production and removal of ROS and NOS were discussed above, and it is evident that these two processes are closely interconnected. Living organisms have finely tuned systems to keep ROS levels very low, meaning that their generation and elimination are balanced, leading to a certain steady-state ROS level. However, under specific conditions, this balance may be disrupted. Several factors contribute to this disruption can be given as follows:

(i) An increased level of endogenous and exogenous compounds undergoing autoxidation associated with ROS generation.
(ii) Depletion of low-molecular-weight antioxidant reserves.
(iii) Inactivation of antioxidant enzymes.
(iv) Reduced production of antioxidant enzymes and low-molecular-weight antioxidants; and finally
(v) Particular combinations of two or more of these factors.

An increase in the steady-state ROS level, resulting from an imbalance between generation and elimination processes. These factors can undoubtedly impact many, if not all, biological processes. The effects of this increase vary depending on the level and location of ROS generation, the efficiency of antioxidant systems, the availability of plastic and energy resources, and the cellular targets involved (Sies 1997; Yapıcı et al. 2021). Under normal conditions, ROS levels fluctuate within a specific range, controlled by the coordinated function of generation and elimination systems. Certain factors, such as the introduction of specific oxidants, may cause a sharp rise in ROS levels, pushing them beyond the usual control (or resting) range. If antioxidant systems are able to manage this surge effectively, ROS levels can return to their original range. This type of event is often referred to as “acute oxidative stress”. It is important to note, however, that merely having an elevated ROS level for a period does not, in itself, constitute oxidative stress. To be classified as oxidative stress, it must have some distinct physiological impacts. One of the most studied examples is the increased expression of antioxidant and related enzymes, such as SOD, catalase (CAT), and glutathione reductase (GR) (Lushchak 2011a). In some cases, the cell fails to neutralize the excess ROS, preventing the ROS level from returning to its initial range. Even an increase in the expression of antioxidant enzymes might not be enough to achieve this. Consequently, ROS levels may stay slightly elevated, or the initial range may need to be broadened. This elevated ROS level can then become stabilized, leading to the modification of various cellular components and causing significant disruptions to homeostasis. This state is referred to as “chronic oxidative stress.” (Bursal et al. 2020).

Another scenario could occur following oxidative surges or physiological changes within the organism. ROS levels might not return to the original range and instead stabilize at a new, so-called “quasi-stationary level” (Lushchak 2011b). This state demands a substantial reorganization of the overall homeostatic mechanisms, including ROS regulation. Several pathologies, such as cancer (Townsend et al. 2014), diabetes mellitus (Yan 2014), cardiovascular diseases (Mei et al. 2014), and neurodegenerative disorders (Ahmad et al. 2014), exemplify chronic oxidative stress. It is essential to investigate these situations carefully to determine whether oxidative stress causes these diseases or if the reverse is true. In many cases, these conditions are observed simultaneously, and it is often unclear if this is merely coincidental. Furthermore, these pathological states can be examined in the context of chronic oxidative stress and quasi-stationary ROS levels (Lushchak 2014; Polat Köse et al. 2020).

Oxidative stress can be induced not only by externally added oxidants and compounds that either stimulate ROS production or weaken antioxidant defenses (Artunç et al. 2020). While an increase in external oxygen levels might be expected to cause oxidative stress and potential tissue injury (Zara et al. 2013), and ischemia/reperfusion could similarly affect biological systems (Sari et al. 2014), hypoxia-induced oxidative stress was somewhat unexpected but has been well supported by experimental evidence to date (Lushchak and Bagnyukova 2007).

Oxidative stress is a concept that has gained significant attention in medical sciences over the past 30 years. It plays a key role in the development and progression of many widespread diseases, including diabetes, hypertension, preeclampsia, atherosclerosis, acute kidney failure, Alzheimer’s disease, and Parkinson’s disease (Taslimi et al. 2020; Karakaya et al. 2020). The generation of ROS is essential for maintaining cellular homeostasis. Living organisms employ an antioxidant defense system to achieve this balance, which helps them manage the equilibrium between oxidative stress and antioxidant protection (Huyut et al. 2017a; Oztaskin et al. 2017). ROS are produced during normal cellular metabolism and can adversely affect critical biomolecules such as lipids, carbohydrates, proteins, and nucleic acids (Cetin Cakmak and Gulcin 2019). Living organisms are continually exposed to ROS, which are generated as by-products of metabolic processes, normal respiration, and the autoxidation of xenobiotics. These species can also arise from the stress associated with various diseases (Anraku et al. 2018; Turkan et al. 2020). Oxidative stress occurs when there is an imbalance between ROS and the body's antioxidant defenses. This stress can disrupt numerous cellular functions and contribute to a range of pathological conditions. Typically, the rate and extent of oxidant production are balanced by their removal. However, when the equilibrium between pro-oxidants and antioxidants is disrupted, oxidative stress occurs. Elevated ROS levels in cells significantly affect cellular function, contributing to impaired cell performance, aging, or the onset of disease (Munteanu and Apetrei 2021). When ROS levels exceed the organism's antioxidant defenses, they can lead to oxidative modifications of essential biological macromolecules, tissue damage, and accelerated cell death. This phenomenon underlies many diseases (Sindhi et al. 2013; Apak et al. 2022).

### Antioxidants

Antioxidants are compounds that help protect the body’s cells from damage caused by harmful molecules called free radicals. Antioxidants are substances that, even in very small amounts in foods or the human body, can slow down, manage, or block oxidative processes. This ability helps to prevent the decline in food quality and may also hinder the onset and spread of degenerative diseases within the body. Various methods and actions contribute to how these antioxidant compounds work to inhibit oxidation (Shahidi and Zhong 2011). They are crucial in both food systems and the human body, as they help reduce oxidative processes and mitigate the harmful effects of ROS (Cakmakci et al. 2015; Gocer et al. 2013). In food systems, antioxidants delay lipid peroxidation and prevent the formation of secondary lipid peroxidation products including 4-hydroxynonenal and malon dialdehyde (MDA). This action is essential for preserving the flavor, color, and texture of food products during storage (Bursal et al. 2013; Cakmakci et al. 2015). Furthermore, antioxidants help reduce biomolecules including amino acid and protein oxidation and prevent the interaction of lipid-derived carbonyls with proteins, which can alter protein functionality (Sindhi et al. 2013). An antioxidant is defined as a molecule that can inhibit or delay the oxidation of other molecules. In the context of food, an antioxidant was initially defined as “any substance that, when present at low concentrations compared to that of an oxidizable substrate, significantly delays or inhibits the oxidation of that substrate.” In terms of biochemistry, antioxidants were defined as “any substance that delays, prevents, or removes oxidative damage to a target biological molecule” (Halliwell and Gutteridge 1989; Sies 1993; Halliwell 1995). An alternative definition of antioxidants describes them as “any substance that directly neutralizes ROS and free radicals or indirectly enhances antioxidant defenses or curtails ROS production.” It is also worth clarifying an important point here. What is the difference between the terms “antioxidant activity” and “antioxidant capacity” in terms of the clarity of antioxidant concepts? The term “antioxidant activity” should, in principle, be used to describe the antioxidant properties of a single compound in a specific assay. In contrast, the term “total antioxidant capacity” (TAC) refers to the antioxidant properties of a complex material (such as a beverage, extract, or biological fluid) composed of many compounds in the same assay. Furthermore, the term “antioxidant activity,” used in a broader sense (as a concept rather than a measurable parameter), encompasses various properties of an antioxidant, such as redox potential and reaction rate constants with different oxidants. Therefore, in this study, the term “TAC” is used as the parameter to characterize the antioxidant behavior of complex samples (Bartosz 2003; Sadowska-Bartosz and Bartosz 2022). Antioxidants have the ability to scavenge free radicals, extending shelf life by slowing lipid peroxidation, a primary cause of food and pharmaceutical product degradation during processing and storage (Halliwell 1997; Gülçin 2020).

Antioxidants can be categorized in multiple ways. Based on their mechanism of action, antioxidants can be classified as primary and secondary antioxidants. However, the human antioxidant system is categorized into two main groups including enzymatic antioxidants and non-enzymatic natural antioxidants. Additionally, synthetic antioxidants can be mentioned as another group of antioxidants.

#### Enzymatic antioxidants

Enzymatic antioxidants are further divided into primary and secondary enzymatic defenses. There are several intracellular enzymes that produce oxidants, or ROS, however, the primary defense comprises three key enzymes that prevent free radical formation or neutralize them: glutathione peroxidase (GPx), catalase (CAT), and superoxide dismutase (SOD). GPx donates two electrons to reduce peroxides by forming selenols and eliminates peroxides that could otherwise act as substrates for the Fenton reaction. CAT, with one of the highest known turnover rates, converts H_2_O_2_ into H_2_O and O_2_, enabling a single CAT molecule to transform six billion H_2_O_2_ molecules. Lastly, SOD converts ${\text{O}}_{2}^{ \cdot - }$ into H_2_O_2_, which then serves as a substrate for CAT (Rahman 2007; Carocho and Ferreira 2013). The secondary enzymatic defense includes glutathione reductase (GR) and glucose-6-phosphate dehydrogenase (G6PD). GR regenerates glutathione by reducing it from its oxidized form, allowing it to continue neutralizing additional free radicals. G6PD regenerates nicotinamide adenine dinucleotide phosphate (NADPH), a coenzyme involved in anabolic reactions, thereby maintaining a reducing environment. Although both enzymes do not directly neutralize free radicals, they play essential supporting roles for other endogenous antioxidants (Carocho and Ferreira 2013). Antioxidant enzymes neutralize harmful oxidative products by converting them into H₂O₂ and subsequently into water through a multi-step process that requires cofactors like copper, zinc, manganese, and iron. On the other hand, non-enzymatic antioxidants function by disrupting free radical chain reactions. Examples of non-enzymatic antioxidants include vitamins C and E, plant polyphenols, carotenoids, and glutathione (Shahidi and Zhong 2010; Nimse and Pal, 2015).

**Fig. 4:**
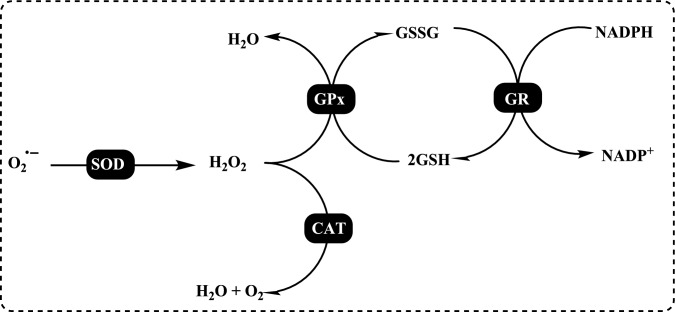
Chemical reactions catalyzed by antioxidant enzymes in the cellular system. The superoxide anion (O_2_^•−^) is converted into H_2_O_2_ through the action of SOD. Catalase facilitates the dismutation of H_2_O_2_, which contains a heme group. GPx catalyzes the reduction of hydroperoxides by using electrons transferred from NADPH via GR and reduced glutathione (GSH)

The antioxidant enzymes can use as biomarkers in various human diseases, because they are the first to indicate the redox state through oxidation and reduction processes (Yang and Lee 2015). The level of H_2_O_2_ or hydroperoxides and other ROS such as O_2_^•−^, OH^•^ and ^1^O_2_ can be elevated either by their enhanced production or the decreased activity of the defence system. Under oxidative stress conditions the antioxidant enzymes activities such as SOD, CAT, GR, and GPx are generally increased in the metabolism and, in many cases the activities of antioxidant enzymes correlate well with enhanced tolerance (Foyer et al. 1997; Apak et al. 2022). However, these enzymes are often insufficient in fully preventing degenerative diseases and other health issues (Borneo et al. 2009). The role of antioxidant enzymes in regulating oxidative stress related to vascular disease has been previously highlighted (Leopold and Loscalzo 2009). Antioxidant enzymes can serve as biomarkers for various human diseases, as they are the first indicators of redox status through oxidation and reduction processes (Yang and Lee 2015). The levels of H_2_O_2_ or hydroperoxides, along with other ROS such as O_2_^•−^, OH•, and ^1^O_2_, can be elevated either due to increased production or decreased activity of the defense system (Fig. 4). During oxidative stress, the activities of antioxidant enzymes like SOD, CAT, GR, and GPx are generally elevated, and in many cases, the activity levels of these enzymes are closely correlated with improved tolerance (Foyer et al. 1997).

#### Superoxide dismutase

Cells continuously generate superoxide anion (O_2_^•−^) as a by-product of normal aerobic metabolism. Superoxide dismutase (SOD, E.C.1.15.1.1) as ubiquitous metalloenzyme, found in almost all aerobic organisms, including plants, as well as in all intracellular organelles and apoplastic spaces. SOD plays an active role at the forefront of defense against oxidative damage caused by ROS. The main function of SOD is to convert superoxide radicals (O₂^•^⁻) into H₂O₂ and O₂, thereby preventing the formation of OH• through the metal-catalyzed Haber–Weiss reaction (Apak et al. 2022).Superoxide dismutase (SOD, E.C.1.15.1.1) acts as the primary defense against O_2_^•−^ by catalyzing its dismutation into oxygen and H_2_O_2_, a non-radical reactive oxygen species, along with O_2_ (Ragsdale 2009). While H_2_O_2_ can also be harmful, its effects are less severe, and it is reduced by other antioxidant enzymes, such as CAT. Thus, SOD plays a crucial role in antioxidant defense in almost all living organisms exposed to the damaging effects of molecular oxygen. Depending on the metal cofactor, SODs are classified into Cu/ZnSOD, MnSOD, and extracellular SOD. MnSOD is typically found in peroxisomes and mitochondria, while Cu/ZnSOD is generally the most abundant form and is located in the cytosol. The extracellular SOD is the excreted form of Cu/ZnSOD (Reddi et al. 2009).

Since the reaction is limited only by the frequency of collisions between SOD and O_2_^•−^, SOD plays a key role in antioxidant defense. The dismutation reaction catalyzed by SOD can be represented for Cu/ZnSOD, which is commonly found in eukaryotes, including humans. The scavenging of the O_2_^•−^ by SOD is shown in the following reactions.

$${\text{O}}_{{2}}^{ \bullet - } + {\text{ Cu}}^{{{2} + }} - {\text{SOD }} \to {\text{O}}_{{2}} + {\text{ Cu}}^{ + } - {\text{SOD}}$$

$${\text{O}}_{{2}}^{ \bullet - } + {\text{ 2H}}^{ + } + {\text{ Cu}}^{ + } - {\text{SOD }} \to {\text{H}}_{{2}} {\text{O}}_{{2}} + {\text{ Cu}}^{{{2} + }} - {\text{SOD}}$$

In a general sense, the following reactions can be applied and written for all the different metal-coordinated forms of SOD (where n = 1 for Cu and n = 2 for Mn, Fe, and Ni, with M representing the metal).

$${\text{O}}_{{2}}^{ \bullet - } + {\text{ M}}^{{({\text{n}} + {1})}} + - {\text{SOD }} \to {\text{O}}_{{2}} + {\text{ M}}^{ + } - {\text{SOD}}$$

$${\text{O}}_{{2}}^{ \bullet - } + {\text{ 2H}}^{ + } + {\text{ M}}^{ + } - {\text{SOD }} \to {\text{H}}_{{2}} {\text{O}}_{{2}} + {\text{ M}}^{{\left( {{\text{n}} + {1}} \right) + }} - {\text{SOD}}$$

### Catalase

Catalase (CAT, E.C. 1.11.1.6) is an iron-dependent enzyme with two different functions. This Fe-containing homotetrameric CAT is responsible for the catalysation of H_2_O_2_ overproduced during light respiration or photorespiration in peroxisomes, H_2_O_2_ produced during β-oxidation of fatty acids in glyoxysomes or H_2_O_2_ produced by SOD (Zandi and Schnug 2022). It can function either catalytically or peroxidatively (Tokarz et al. 2013). In mammals, including humans, CAT is present in all tissues and organs. CAT facilitates the breakdown of H_2_O_2_ into H_2_O and O_2_ with a high catalytic efficiency, utilizing either an Fe or Mn cofactor (Gülçin et al. 2005a). The enzyme is encoded by a single, highly conserved gene across species, and its expression is regulated at transcriptional, post-transcriptional, and post-translational levels. High concentrations of CAT are found in erythrocytes, the kidneys, and the liver (Nishikawa et al. 2002; Kang and Won 2013). The reaction by which CAT decomposes H_2_O_2_ in living tissue is as follows:

$${\text{2H}}_{{2}} {\text{O}}_{{2}} \to {\text{ 2H}}_{{2}} {\text{O }} + {\text{ O}}_{{2}}$$

However, it was estimated that this reaction consists of two steps.

$$\frac{\begin{gathered} {\text{H}}_{{2}} {\text{O}}_{{2}} + {\text{ Fe}}^{{{3} + }} - {\text{E }} \to {\text{ H}}_{{2}} {\text{O }} + {\text{ O}} = {\text{Fe}}^{{{4} + }} - {\text{E}}\left( {. + } \right) \hfill \\ {\text{H}}_{{2}} {\text{O}}_{{2}} + {\text{ O}} = {\text{Fe}}^{{{4} + }} - {\text{E}}\left( {. + } \right) \, \to {\text{ H}}_{{2}} {\text{O }} + {\text{ O}}_{{2}} + {\text{Fe}}^{3 + } - {\text{E}} \hfill \\ \end{gathered} }{{{\text{2H}}_{{2}} {\text{O}}_{{2}} \to {\text{ 2H}}_{{2}} {\text{O }} + {\text{ O}}_{{2}} }}$$

Here, Fe^3+^-E represents the iron center of the heme group in CAT. Fe^4+^-E(. +) is a resonance form of Fe^5+^-E, indicating that the iron is not fully oxidized to + V but instead receives partial electron support from the heme ligand. Consequently, the heme should be depicted as a radical cation (. +).

### Glutathione reductase

**Fig. 5:**
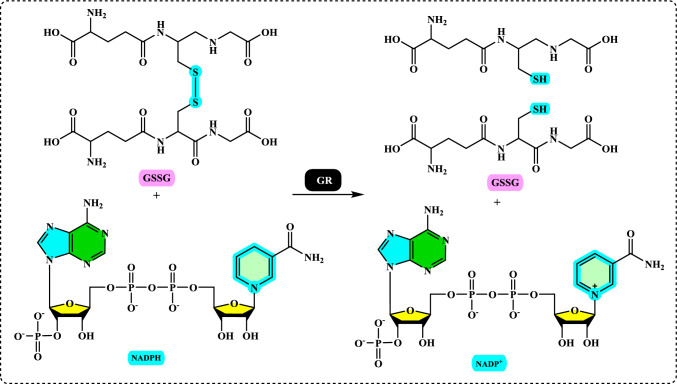
Chemical reactions catalysed by glutathione reductase (GR)

Glutathione (GSH) is composed of the amino acids glutamic acid, cysteine, and glycine. It is widely recognized for its antioxidant properties within the central nervous system and plays a crucial role in maintaining redox homeostasis. GSH is present at millimolar concentrations in many cell types. It neutralizes various reactive oxygen and nitrogen species, including O_2_^•−^, NO, OH^•^, and ONOO^•^, within cells (Aoyama et al. 2008; Zhang et al. 2013). As a key antioxidant, GSH participates in the decomposition of H_2_O_2_, a process that can be catalyzed by selenium-dependent glutathione peroxidase (GPx). The studies have shown an age-related decline in plasma GSH levels, while the levels of glutathione disulfide (GSSG), the oxidized form of GSH, increase with age in whole blood (Kretzschmar and Müller 1993). Additionally, GSH depletion can contribute to oxidative stress. Elevated GSH levels can result from at least two processes: one involves increased GSH biosynthesis and a higher rate of GSSG conversion back to GSH via Glutathione reductase (GR, E.C.1.8.1.7) (Tokarz et al. 2013). This enzyme also known as flavoprotein oxidoreductase, is the last in the ascorbate–glutathione cycle that is found predominantly in chloroplasts and partially in the cytosol and mitochondria. It catalyzes the reduction of GSSG to its sulfhydryl form as 2GSH (Fig. 5).

GSH is essential for combating oxidative stress and preserving the cell's reducing environment. In the second process, GR utilizes an FAD prosthetic group and NADPH to reduce one mole of GSSG into two moles of GSH (Mannervik 1987; Şentürk et al. 2008).

### Glutathione peroxidase

Peroxidases can contain a heme cofactor in their active site, as seen in ascorbate peroxidases and guaiacol peroxidases. Others, known as non-heme peroxidases, possess redox-active cysteine or selenocysteine residues. Non-heme peroxidases include thiol peroxidases, such as thioredoxin peroxidases and glutathione peroxidase (GPx, E.C.1.11.1.9) (Bela et al. 2015). GPx was the first selenocysteine-containing protein identified in mammals. Initially discovered as an erythrocyte enzyme that specifically reduces H_2_O_2_ via GSH, GPx was later shown to reduce a wide range of organic hydroperoxides, including membrane phospholipids, cholesterol, and long-chain fatty acids, into H_2_O and O_2_ (Toppo et al. 2009). GPx catalyzes the breakdown of H_2_O_2_ and other organic peroxides and contains a single selenocysteine residue essential for its activity. This residue is present in all five distinct isoforms identified to date (El-Far et al. 2005).

- GPx1: Cytosolic (cGPx) and expressed in all cells and tissues, predominantly in erythrocytes, kidneys, and liver (Zotova et al. 2004).
- GPx2: Another cytosolic isoform found primarily in the colon and liver.
- GPx3: The only extracellular GPx isoform (eGPx), found in plasma and primarily localized in the proximal tubule cells of the kidney (Crawford et al. 2012).

The primary reaction catalyzed by GPx is:

$${\text{H}}_{{2}} {\text{O}}_{{2}} + {\text{ 2GSH }} \to {\text{ 2H}}_{{2}} {\text{O }} + {\text{ GSSG}}$$

In this reaction include GSH represents reduced monomeric glutathione and GS-SG denotes glutathione disulfide. The mechanism involves oxidation of the selenol group in the selenocysteine residue by H_2_O_2_, forming a selenenic acid (RSeOH) derivative. The selenenic acid is then regenerated into selenol through a two-step process including the reaction with GSH: Forms GS-SeR and releases H_2_O and reduction of GS-SeR by GSH: Produces GS-SG and restores RSeH. These simplified reactions are as follows (Şentürk et al. 2008):

$$\begin{gathered} {\text{H}}_{{2}} {\text{O}}_{{2}} + {\text{ RSeH }} \to {\text{ H}}_{{2}} {\text{O }} + {\text{ RSeOH}} \hfill \\ {\text{GSH }} + {\text{ RSeOH }} \to {\text{ H}}_{{2}} {\text{O }} + {\text{ GS}} - {\text{SeR}} \hfill \\ {\text{GSH }} + {\text{ GS}} - {\text{SeR }} \to {\text{ GS}} - {\text{SG }} + {\text{ RSeH}} \hfill \\ \end{gathered}$$

Finally, glutathione reductase (GR) reduces oxidized glutathione (GS-SG) to complete the cycle:

$${\text{NADPH }} + {\text{ H}}^{ + } + {\text{ GS}} - {\text{SG }} \to {\text{ 2GSH }} + {\text{ NADP}}^{ + }$$

#### Non-enzymatic antioxidants

Non-enzymatic antioxidants play a crucial role in protecting the human body from the harmful effects of free radicals and ROS, which can contribute to chronic diseases including cardiovascular diseases, diabetes, cancer and arthritis. Chronic diseases are long-term medical conditions that often progress slowly over time and typically last for a year or more. They generally require ongoing medical attention, may impact daily activities, and often cannot be entirely cured. Instead, they are usually managed through lifestyle changes, medications, or other treatments. These chronic diseases are influenced by various risk factors, including genetics, age, lifestyle, environmental factors and especially ROS (Gülçin 2020). Major contributors to chronic diseases include smoking, poor diet, physical inactivity, and excessive alcohol use. Recently, there has been a growing interest in identifying safe, natural food antioxidant sources, particularly those derived from plants. Antioxidants are commonly added to food products to interrupt oxidative chain reactions. They work by inhibiting the initiation and propagation steps, leading to reaction termination and delaying oxidation (Shahidi et al. 1992; Gulcin 2006a). As indispensable food additives, antioxidants extend shelf life without negatively impacting sensory or nutritional quality. However, antioxidants used in food systems must be affordable, effective, and nontoxic at low levels; they should remain stable and retain their effectiveness after processing, have no intrinsic odor, taste, or color, be easy to incorporate, and dissolve well in the product (Shahidi and Ambigaipalan 2015). When evaluating the antioxidant properties of food components, the terms “antioxidant activity” and “antioxidant capacity” are often used interchangeably, though they have distinct meanings. Antioxidant activity refers to the rate of reaction between a specific antioxidant and a particular oxidant, while antioxidant capacity measures the quantity of a given free radical that a sample can neutralize (MacDonald-Wicks et al. 2006). Consuming fruits and vegetables has been linked to a lower risk of certain chronic diseases, including severe coronary atherosclerosis (Rimm et al. 1996). Epidemiological research has shown an inverse relationship between fruit and vegetable intake and mortality from age-related illnesses, such as coronary heart disease and cancer, which may be due to their antioxidant effects (Ganesan et al. 2011). The primary bioactive compounds found in these natural sources, especially phenolics and flavonoids, are credited with their health-promoting properties (Bocco et al. 1998). The antioxidant capabilities of phenolics help prevent the oxidation of low-density lipoprotein and cholesterol (Eberhardt et al. 2000). Consequently, a higher intake of fruits and vegetables is associated with a reduced incidence of coronary atherosclerosis (Rimm et al. 1996). Bioavailability refers to the proportion of a nutrient that is digested, absorbed, and metabolized through standard biological pathways. It is well established that after the intake of foods rich in polyphenols, an increase in plasma antioxidant capacity provides indirect evidence of polyphenol absorption across the intestinal barrier. The absorption rate and extent of polyphenols in the intestine are influenced by their chemical properties and structures. For example, while aglycones are absorbed in the small intestine, polyphenols in the form of esters, glycosides, or polymers cannot be absorbed in their original form. These forms must first undergo hydrolysis by intestinal enzymes like lactase and β-glycosidases or by the colonic microflora before they can be absorbed (Nemeth et al. 2003). Consequently, the forms of polyphenols present in human blood and tissues differ from those in foods, complicating the identification of all metabolites and the assessment of their biological activity. The chemical structure of polyphenols is actually more critical than their concentration, as it determines both the rate and extent of absorption and the nature of the metabolites circulating in the plasma (Cipolletti et al. 2018). A wide range of biologically active phenolic compounds, featuring one or more aromatic rings, naturally occur in plant-based foods. These compounds contribute significantly to the flavor, color, and texture of these foods. The simpler phenolic compounds include monophenols with a single benzene ring, such as 3-ethylphenol and 3,4-dimethylphenol, commonly found in fruits and seeds. Additionally, the hydroxycinnamic acids, like caffeic and ferulic acid, and the flavonoid group, which includes catechins, proanthocyanidins, anthocyanidins, and flavonols, are notable. Tannins, a complex and not fully characterized group of water-soluble phenolics with high molecular weights, are also present. The daily intake of phenolic compounds may reach up to 1 g, though the amount of specifically identified flavonoids in the diet is likely limited to a few tens of milligrams per day (Pokorny et al. 2000).

Phenolic compounds in foods originate from one of the primary classes of secondary metabolites in plants. They function as antioxidants and, even at low concentrations, help protect food from oxidative rancidity (Karakaya 2004). The antioxidant potential of phenolic compounds is influenced by the number and arrangement of hydroxyl groups in their molecular structure. Phenolics become active antioxidants when substitution at either the ortho- or para-position enhances the electron density at the hydroxyl group, which in turn reduces the oxygen–hydrogen bond energy and increases reactivity against lipid free radicals. Substitution at the meta-position, however, has a limited effect. The antioxidant activity and effectiveness of chain-breaking phenolic antioxidants are governed by both steric and electronic factors (Barclay et al. 1993). Molecular orbital theory has been used to understand the hydrogen abstraction mechanism of phenolic antioxidants in the autoxidation chain process (Tomiyama et al. 1993; Gulcin 2012). It is a method in chemistry that explains the bonding, structure, and properties of molecules by describing electrons in terms of molecular orbitals, which are spread over the entire molecule rather than being localized around individual atoms (Robert and Pauling 2005).

Recent studies have shown that bromophenols possess a variety of medicinal properties, including antioxidant (Oztaskin et al. 2015; Boztas et al. 2015, 2019), antimicrobial, anticancer, anti-inflammatory, and antidiabetic effects (Cherian et al. 2019). They also inhibit some enzymes linked to global health concerns, such as carbonic anhydrase (Taslimi et al. 2016), acetylcholinesterase (Oztaskin et al. 2015), butyrylcholinesterase (Bayrak et al. 2017; 2019), aldose reductase (Demir et al. 2018), and paraoxonase (Cherian et al. 2019) α-amylase and α-glycosidase (Taslimi et al. 2018b). Several scientific studies are exploring the various health benefits of antioxidants in relation to stress, pathogen infestation, aging, apoptosis, and neurological disorders. Antioxidants play a crucial role in mitigating the damaging effects of free radicals on cells. Humans obtain antioxidants primarily from fresh and dried fruits and vegetables, which are rich in flavonoids, as well as from antioxidant supplements. These compounds help protect against a range of diseases, including cancers and cardiovascular issues (Sindhi et al. 2013). Although synthetic antioxidants have undergone extensive toxicological testing, some are now facing scrutiny as new toxicological data raise concerns about their safety (Thompson and Moldeus 1988). In this light, natural antioxidants are emerging as healthier and safer alternatives to synthetic ones. Since around 1980, natural antioxidants have been increasingly considered a substitute for synthetic options. Thus, antioxidants can be classified as either synthetic or natural based on their source (Gulcin 2012).

#### Synthetic antioxidants

**Fig. 6:**
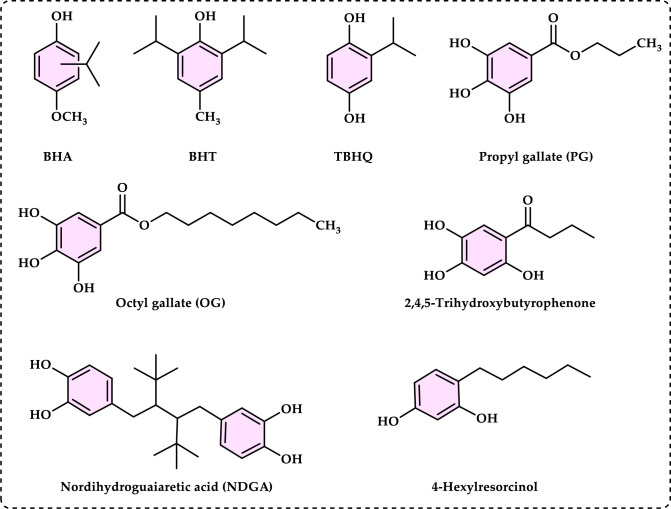
The chemical structures of most putative synthetic antioxidants

Food additives including synthetic antioxidants are commonly used in the food industry for purposes such as preservation, coloring, and enhancing flavor or sweetness. These substances help to prevent or delay nutrient degradation caused by bacterial, enzymatic, or chemical changes in food, and they also extend shelf-life and improve overall product quality. Synthetic antioxidants, in particular, play an essential role in food preservation (Thorat et al. 2013). They play a crucial role in the daily diet as functional additives, yet their usage is restricted by both the United States and the Food and Drug Administration (FDA). Although certain chemical antioxidants have shown no adverse effects on human health when incorporated into food products, many of them still demonstrate significant impacts in both in vitro and in vivo studies. Recent research has revealed that some of these synthetic antioxidants are allowed in functional foods, while being banned in certain countries due to their potential harmful effects (Xu et al. 2021). They have been developed to create a standardized system for measuring antioxidant activity that can be compared with natural antioxidants and incorporated into food. These pure compounds are added to food to ensure its stability under various processes and conditions, as well as to extend its shelf life. Figure 6 shows the most important and widely used synthetic antioxidants to prevent the oxidation of fatty acids. Today, synthetic antioxidants are found in almost all processed foods. Recently, however, food additives and synthetic antioxidants have gained attention as potential contributors to various health issues, including liver and kidney damage, mutagenic effects, and even cancer. Studies have documented the cytotoxic and genotoxic effects of different additives in a range of cell lines. Among various food additives, tert-butylhydroquinone (TBHQ), butylated hydroxyanisole (BHA), and propyl gallate (PG) have shown the ability to interact with DNA structures (Pandir 2016; Yang et al. 2023; Yilmaz et al. 2014). Synthetic antioxidants are chemical compounds that are artificially produced to prevent oxidation in various materials, including food, cosmetics, and industrial products. Synthetic antioxidants have been developed to establish a standardized system for measuring antioxidant activity, allowing for comparisons with natural antioxidants and facilitating their incorporation into food products. These compounds are added to food to enhance stability under various processing conditions and to extend shelf life. Nearly all processed food items contain synthetic antioxidants, which are generally considered safe, although some research suggests potential concerns (Carocho and Ferreira 2013). The primary purpose of these additives is to prevent or slow down lipid oxidation in fats, oils, and lipid-containing foods during processing and storage. The food industry has utilized synthetic antioxidants for approximately 80 years. The market for antioxidants in food and beverages is valued at around $500 billion, with a growth rate of 5–7% per year, projected to continue at this pace through 2017 (Shahidi and Ambigaipalan 2015). Synthetic food antioxidants are widely used across various food industries due to their strong protective properties, which help prevent food spoilage and reduce the risk of foodborne diseases in both humans and animals. As shown in Fig. 6, the most commonly used synthetic antioxidants are phenolic compounds, including BHA, butylated hydroxytoluene (BHT), TBHQ, PG, and octyl gallate (OG) (Gulcin 2012). These primary synthetic antioxidants commonly used are subject to a good manufacturing practice limit of 0.02% of the fat or oil content in foods (Simic 1981). BHA is often used in food products and cosmetics. It helps to prevent rancidity and prolong shelf-life and is particularly effective in inhibiting the oxidation of short-chain fatty acids. Similar to BHA, BHT is used in food packaging and preservation to prevent oxidation. In contrast, BHT is generally less effective than BHA due to the presence of two tert-butyl groups, which create greater steric hindrance in the molecule (Gulcin, 2020).

### Butylhylated hydroxyanisole (BHA)

Butylated hydroxyanisole (BHA) consists of two isomeric organic compounds including 2-tert-butyl-4-hydroxyanisole and 3-tert-butyl-4-hydroxyanisole. It is prepared from isobutylene and 4-methoxyphenol. These compounds can be synthesized through a reaction between methoxyphenol and isobutene (Additives et al., 2018). Since 1947, BHA has been incorporated into edible fats and foods containing fats due to its antioxidant qualities, which help prevent rancidity and the development of unpleasant odors in food (Lam et al. 1979). BHA has long been used as a food antioxidant to stabilize and preserve the nutritional value and sensory qualities of products, such as animal feed and the freshness and flavor of food. It is particularly effective in reducing free radical formation, thus preventing food spoilage and lipid oxidation, which is especially important in fried foods. BHA serves as a preservative in products for human consumption, animal feed, veterinary medications, and related food products. Additionally, BHA is generally recognized as safe by the FDA when used in amounts below 0.02% in foods containing fats or oils, in accordance with good manufacturing practices. BHA is also synergistic when combined with PG (St. Angelo 1996). Furthermore, it was reported that BHA can protect metabolism against the toxicity and radiation poisoning caused by various xenobiotics and mutagens (Adeyemi 2021; Dawidowicz et al. 2015). However, some studies have shown that BHA exhibits a broad spectrum of biological effects in in vivo models (Xu et al. 2021). Research indicates that BHA can act as either a tumor initiator or promoter in various animal tissues (Imbabi et al. 2021). In particular, BHA has been linked to carcinoma development in the stomachs of animals such as hamsters, mice, and rats when consumed in high quantities over extended periods. Additionally, BHA has demonstrated proliferative effects in mammalian and porcine esophageal tissues. Vandghanooni et al. (2013) reported that BHA can induce cytotoxicity, and other studies have shown that BHA promotes HO-1 gene expression in smooth muscle cells. Recent findings also confirm that BHA is associated with acute toxicity, immune responses, activation of phase II detoxification enzymes, and tumor-promoting activities. Consequently, the extensive use of BHA in food products could pose risks of cytogenetic and molecular toxicity (Esazadeh et al. 2024).

### Butylhylated hydroxytolune (BHT)

Butylated hydroxytoluene (BHT) also known as dibutylhydroxytoluene is a synthetic antioxidant commonly used as a preservative to prevent the oxidation of fats and oils in food, cosmetics, pharmaceuticals, and other products. It is a white crystalline powder and its color turns yellow and darkens gradually when exposed to light. It is an excellent antioxidant additive for various plastic, rubber and petroleum products. It is an aromatic compound with a hydroxyl group attached to a methylated toluene ring (Yehye et al. 2015). It was reported that phytoplankton, including the green algae (*Botryococcus braunii*) as well as three different cyanobacteria (*Cylindrospermopsis raciborskii*, *Microcystis aeruginosa* and *Oscillatoria* sp.) are capable of producing BHT as a natural product (Babu and Wu 2008). The fruit lychee also produces BHT in its pericarp (Jiang et al. 2013). Several fungi like *Aspergillus conicus* living in olives produce BHT (Gharbi et al. 2017). This structure enables it to act as a free radical scavenger. In the pharmaceutical industry, BHT is used as an antioxidant in medicinal and vitamin formulations, preserving the active ingredients by preventing degradation from oxygen and light exposure. This protection helps maintain the potency and effectiveness of pharmaceutical products. It helps maintain freshness and prolong shelf life by inhibiting the chemical reactions that cause spoilage, rancidity, and loss of flavor. BHT works by scavenging free radicals, which can otherwise react with and degrade food components. Like BHA, BHT is Generally Recognized As Safe (GRAS) by the FDA in small amounts. However, its use is controversial, as high doses have shown toxic effects in animal studies. Regulatory agencies set limits on BHT concentration to ensure consumer safety. BHA and BHT are relatively heat-stable, making them ideal for stabilizing fats in baked and fried products. Antioxidants that withstand high temperatures possess what is known as “carry-through” properties (Gulcin 2020).

BHT is a lipophilic organic compound, meaning it is fat-soluble and binds well with oils. It has antioxidant properties due to its phenolic structure, which allows it to neutralize free radicals that can cause oxidation. The European Food Safety Authority (EFSA) reviewed BHT and concluded it is safe at regulated levels in food. However, the EFSA noted that at high doses, BHT could potentially cause adverse effects in animals, making its long-term safety a concern for human use in high amounts. Another comprehensive review discusses both the antioxidant and pro-oxidant effects of BHT, underlining its complex role in health. It emphasizes that, while BHT is generally safe at low doses, caution is warranted with prolonged exposure or high doses (Witschi et al. 1986). It has been described in various ways: as a prooxidant, antioxidant, anticarcinogen, carcinogen, and tumor promoter. Additionally, BHA has demonstrated anticarcinogenic properties at concentrations close to those typically used as food additives. Nevertheless, BHT and BHA are the most commonly used synthetic antioxidants, approved for food and pharmaceutical applications and authorized as food additives by several national regulatory bodies (Shahidi and Wanasundara 1992). When BHA and BHT are used together to create synergistic effects (Omura 1995), Legislative bodies have imposed restrictions on BHA and BHT due to suspicions regarding their toxic and carcinogenic effects (Sherwin 1990). BHA has been found to be slightly superior to BHT in its carry-through properties (Shahidi and Ambigaipalan 2015). Consequently, there is a widespread view that these concerns may be addressed by substituting synthetic antioxidants with effective natural antioxidants for broader use in the food and medicinal sectors (Anraku et al. 2018). The FDA regulates synthetic antioxidant use, fueling the need to identify effective antioxidants from natural sources. This has led to increased interest in natural, safer antioxidants for food applications and a consumer-driven preference for natural antioxidants, motivating the exploration of natural antioxidant sources (Gülçin 2006b, 2007).

### Tert-butylhydroquinone (TBHQ)

TBHQ, is a metabolite derived from 3-tert-butyl-4-hydroxyanisole. Known for being a hydroquinone derivative with a tert-butyl group, TBHQ is primarily synthesized through chemical methods. It appears as a light-colored crystalline solid with a faint odor, exhibiting solubility in ethanol but not in water. Additionally, TBHQ is effective in preserving the color of iron-containing foods, preventing discoloration (Van Esch 1986). It is a highly effective antioxidant and commonly used in various food products, including different types of meats, animal fats, unsaturated oils, and other oils, to protect against oxidative deterioration. By adding TBHQ at levels below 0.02%, the onset of rancidity can be delayed, thereby extending the shelf life of these products. Notably, TBHQ does not alter the color of foods, even in the presence of iron, nor does it affect the taste or smell of the foods it is added to. Additionally, it functions as a stabilizer, preventing auto-polymerization in certain foodstuffs (Esazadeh et al. 2024). TBHQ is commonly used in frying oils and processed foods and helps to stabilize products and extends their shelf-life. TBHQ is used in foods containing fats and oils to prevent spoilage (Nanditha and Prabhasankar 2009). Also, TBHQ is considered the most suitable antioxidant for vegetable oils. While these ingredients are legally permitted in food products, they could be harmful if absorbed by the body in amounts exceeding acceptable limits. Beyond its role in food preservation, TBHQ is also widely utilized in the cosmetics industry as an antioxidant in products like blush, eyeshadow, and lipstick, typically at concentrations below 0.1%. It is frequently found in foods rich in fats, as it is an approved food antioxidant in regions such as the United States, the European Union, and Australia. TBHQ's key benefit is its stability at high temperatures, helping foods maintain antioxidant capacity during cooking processes, including frying at 180 °C and above (Beker et al. 2016; Hojjati-Najafabadi et al. 2020). However, in large amounts, TBHQ has been shown to have several side effects, including changes in DNA structure and the development of stomach tumors in animal studies (Blundell et al. 2022). Recent research has confirmed TBHQ’s nephrotoxic effects in rats, as well as cortical apoptosis and traumatic brain damage in mice. Studies suggest that TBHQ could lead to increased production of 8-hydroxydeoxyguanosine in calf thymus DNA due to the generation of ROS. Interestingly, while high doses of TBHQ have been observed to induce cancer cell death, they also appear to paradoxically enhance carcinogenic effects in laboratory animals. However, the impact of TBHQ on breast cancer specifically remains largely unexplored. Some researchers have proposed a possible link between the use of TBHQ in processed foods and the prevalence of certain diseases, such as breast cancer (Kashanian and Dolatabadi 2009; Esazadeh et al. 2024). Among various food additives, BHA, TBHQ, and PG have been reported to potentially interact with nucleic acid structures, potentially damaging DNA’s double-helix structure (Dolatabadi and Kashanian 2010). However, consumer acceptance of these additives has decreased, as their synthetic nature has raised safety concerns about their metabolism, absorption, and possible accumulation in cells, tissues, organs, and the body (Kulawik et al. 2013; Anraku et al. 2018). It is widely used as a food antioxidant due to its low cost, effectiveness, and broad availability. While it has previously been evaluated by the former Scientific Committee on Food (SCF), additional information was still required. The acceptable daily intake (ADI) for TBHQ is set between 0 and 0.7 mg/kg for body weight. When TBHQ is used in combination with other antioxidants like PG and BHA, their individual levels should be reduced proportionally (Eskandani et al. 2014).

TBHQ is a synthetic antioxidant widely used as a preservative in food products to extend shelf life. It is particularly effective in stabilizing fats and oils, preventing them from becoming rancid. It helps maintain the freshness, nutritional quality, color, and flavor of animal food items without causing any discoloration, even when iron is present. Moreover, TBHQ does not alter the taste or smell of the substances it is added to (Kashanian and Dolatabadi 2009). Research indicates that among synthetic antioxidants, TBHQ outperforms BHA and BHT, proving to be the most potent antioxidant in borage and evening primrose oil. The antioxidant properties of TBHQ are attributed to its two para-hydroxyl groups (Nanditha and Prabhasankar 2009; Shahidi and Ambigaipalan 2015).

### Propyl gallate (PG)

Propyl gallate (PG), an ester formed from gallic acid and propanol, is a synthetic antioxidant widely used as a food preservative to prevent spoilage and extend shelf life. It is commonly found in processed foods like oils, baked goods, meats, soups, chewing gum, and cosmetics. PG is a widely recognized antioxidant used to prevent rancidity in oils, fats, and fat-containing foods by inhibiting the formation of peroxides. Since 1948, it has been applied to stabilize food and cosmetic packaging materials, fat-containing foods, and as an additive in edible oils, mayonnaise, shortenings, pressure-sensitive adhesives, baked goods, lubricants, and transformer oils. It is produced by extracting the pods of the Tara tree using propan-1-ol, followed by purification. Additionally, PG can be synthesized through a chemical reaction between propyl alcohol and gallic acid, which involves condensation to remove any excess alcohol (Nguyen et al. 2021). PG is widely used as a synthetic antioxidant across various industries, including cosmetics and food, to prevent fat rancidity and oxidative degradation, particularly for protecting polyunsaturated fats (Dolatabadi & Kashanian 2010). It is often used alongside other food antioxidants, such as BHA and TBHQ. Unlike PG, OG have a longer chain structure, which enhances their solubility in fats and stability during food processing. Studies have shown that PG can impact lipid peroxidation in sunflower and sorghum seedlings. Gallate is a vital antioxidant-based hepatoprotective agent in both in vitro and in vivo models (Javaheri-Ghezeldizaj et al. 2023). PG may also offer promising applications for controlling pericarp browning, inhibiting tyrosinase activity, and extending the shelf life of commercially harvested fruits (Xu et al. 2021). Furthermore, PG has been reported to inhibit microbial growth by blocking nucleic acid synthesis and respiration. Its cytoprotective and antioxidative properties have recently been explored (Dolatabadi et al. 2014).

Due to the widespread use of PG as an antioxidant across various industries, its cytotoxic and genotoxic effects have been thoroughly studied in both in vitro and in vivo models (Silva et al. 2017). Although PG exhibits low toxicity, it can still negatively affect the normal functions of targeted cells and tissues. In another study, it was shown potential liver toxicity and has been linked to an increased risk of carcinogenesis. Recent studies suggest that PG may lead to mitochondrial dysfunction and inhibit cellular respiration, resulting in ATP depletion (Dolatabadi et al. 2014; Shahidi and Ambigaipalan 2015). Toxicological assessments of chemical antioxidants like PG have been conducted using DNA fragmentation, DAPI and MTT assays. While PG is generally regarded as an antioxidant, recent findings indicate that it cannot only reduce intracellular glutathione levels but also increase ROS by inhibiting enzymes such as SOD and CAT. In the presence of Cu^2+^ ions, PG may cause single-strand DNA breaks, though these are minimal. However, when PG converts to gallic acid in the presence of metal ions, it is known to induce DNA breaks. PG is suggested to play a critical role in the cytotoxic and potentially carcinogenic effects of gallate (Additives et al. 2020; Javaheri-Ghezeldizaj et al. 2023). Some studies have indicated that PG and its metabolites exhibit liver toxicity and may promote carcinogenesis. PG is GRAS by regulatory agencies such as the FDA, though its use is limited in concentration. Some people may experience allergic reactions, and there is ongoing debate about its long-term health effects, but studies indicate it's safe within regulated amounts (Shahidi and Ambigaipalan 2015). Gallates, such as PG, however, have disadvantages, including a tendency to form dark precipitates when interacting with iron ions and a sensitivity to heat (Omura 1995).

### Octyl gallate (OG)

Octyl gallate (OG), octyl ester of gallic acid, is an antioxidant compound used as a food preservative and an additive in cosmetics and pharmaceuticals. Chemically, it is an ester formed from gallic acid and octanol. Its main function is to prevent oxidation in products, which helps preserve flavor, color, and overall shelf life. It appears as a white to slightly yellowish powder and is often included in products susceptible to oxidative degradation, such as oils, fats, and emulsions. OG is the ester formed from 1-octanol and gallic acid and serves as an antioxidant and food preservative (Koudelka et al. 2015). Generally, OG was GRAS in small quantities, though some individuals may have mild sensitivities to it. Excessive intake of OG is not recommended as, like other phenolic compounds, it can be toxic at high levels. However, OG is an antioxidant that has shown antitumor, antidiabetic and antiamyloidogenic effects. Its antioxidant properties help protects against the oxidation of unsaturated fats, thereby extending the shelf life of processed foods. It is a compound used primarily as an antioxidant in the food, cosmetic, and pharmaceutical industries. OG is used as a food additive to prevent rancidity in fats, oils, and various food products and also commonly found in cosmetics, especially those containing fats and oils. It helps prevent product degradation due to exposure to air and light, maintaining quality and stability. In medications, OG functions as an excipient, protecting active ingredients sensitive to oxidation. This property makes it valuable in formulations where stability is critical. While OG is generally recognized as safe in moderate amounts, excessive exposure or ingestion may have some health risks. Studies have shown that it has potential allergenic effects, with reports of contact dermatitis in sensitive individuals. Moreover, although OG has antioxidant effects, certain studies have raised concerns about its potential for cytotoxicity in high concentrations. These studies have suggested that high doses of these synthetic antioxidants may lead to liver damage and carcinogenesis in laboratory animals. To enhance their solubility in fats and oils, synthetic antioxidants are typically modified with alkyl groups (Hudson 1990). Due to concerns about their potential toxicity, the use of synthetic antioxidants in food is strictly regulated by governments. Their application as food preservatives is limited (Biparva et al. 2012; Shahidi and Ambigaipalan 2015).

### Nordihydroguaiaretic acid (NDGA)

Nordihydroguaiaretic acid (NDGA, 4-[4-(3,4-dihydroxyphenyl)-2,3-dimethylbutyl]benzene-1,2-diol) is a phenolic lignan derived from the evergreen desert plant known as Creosote bush (*Larrea tridentata*). In natural products chemistry, the Creosote bush is particularly notable for its high concentration of NDGA, which accumulates on the surface of its leaves as the plant’s main metabolite. NDGA makes up between 5 and 10% of the dry weight of the leaves and constitutes about 80% of all phenolic compounds present in the plant’s resin (Arteaga et al. 2005). During the 1940s, NDGA was used as a food antioxidant in the United States, but in 1970, the FDA restricted its use due to toxicity concerns. Nowadays, NDGA is used as an antioxidant in the preservation of natural and synthetic rubber. NDGA’s structure allows it to donate an electron and a proton from each of its four hydroxyl groups in the two catechol rings, converting itself into an oxidized catechol-quinone (Yam-Canul et al. 2008). Studies have shown that a diet high in NDGA (2%) can lead to cystic nephropathy in rats (Evan and Gardner Jr. 1979). Additionally, NDGA may have prooxidant effects in the liver and kidneys, especially in time- and dose-dependent situations, both in vivo and in vitro (Sahu et al. 2006). Thus, it appears that NDGA’s antioxidant or prooxidant effects may vary based on its concentration and the cellular environment. With two catechol groups, NDGA’s four phenolic hydroxyl groups can react with ROS, making it a strong antioxidant. NDGA has also shown neuroprotective effects, such as guarding against amyloid-β neurotoxicity in cultured rat hippocampal neurons (Hernández-Damián et al. 2014; Goodman et al. 1994). Several synthetic routes for NDGA have been reported, with one of the latest involving Ti-induced carbonyl coupling, which yields NDGA and its stereoisomer in high quantities (Gezginci and Timmermann 2001). NDGA is known for its potential as a cancer chemopreventive agent, and multiple mechanisms have been proposed for this activity. From an antioxidant standpoint, NDGA therapy could be beneficial for chemoprevention in patients at risk or in the early stages of carcinogenesis, where continuous oxidative stress and exposure to carcinogens may initiate the transformation of normal cells into cancerous ones (Milkovic et al. 2017). It has demonstrated antimutagenic effects in a variety of model systems. The DNA-protective effect of NDGA is thought to be due to its ability to donate phenolic hydrogens, which neutralize hydroxyl radicals formed via Fenton chemistry. Furthermore, the NDGA phenolate anion can chelate ferrous ions, helping prevent further iron-induced hydrogen peroxide oxidation (Lambert et al. 2004). Studies also indicate that NDGA has antihyperglycemic effects in rat and mouse models of Type II diabetes (Reed et al. 1999).

### 2,4,5-Trihydroxybutyrophenone (THBP)

2,4,5-Trihydroxybutyrophenone (THBP) is a synthetic antioxidant. Structurally, it is a phenolic compound, meaning it has a benzene ring with hydroxyl (OH) groups attached at the 2nd, 4th, and 5th positions, as well as a butyrophenone side chain (a four-carbon side chain attached through a carbonyl group). This specific arrangement of hydroxyl groups makes THBP effective at scavenging free radicals, helping to prevent the oxidation of fats, oils, and other compounds in food products and industrial applications. THBP was developed to help extend the shelf life of food by preventing oxidative damage, especially in fatty foods. However, due to concerns over the safety of certain synthetic antioxidants, its usage is limited, and natural antioxidants are often preferred today (Shahidi and Zhong 2005; Pokorny 2007).

### 4-Hexylresorcinol (4-HR)

4-Hexylresorcinol (4-HR) is a phenolic lipid derivative naturally synthesized by higher plants. It possesses antimicrobial and antiparasitic properties and has been included as an active ingredient in topical antiseptics and throat lozenges. As a tyrosinase and PPO inhibitor, 4-HR is used as a food additive to prevent browning in fruits and shrimp melanosis (Arias et al. 2007). Its antioxidant properties have also led to its use in anti-aging creams and other cosmetic products (Chaudhuri 2015). When combined with cisplatin, 4-HR demonstrates an anticancer effect by inhibiting the proliferation of squamous carcinoma cells and tumor growth in xenograft models (Kim and Choi 2013; Kim and Seok 2020). Recently, 4-HR has been integrated into various biomaterials, such as bone substitutes, silk, dental implants, and polymers (Kim and Seok 2020). It has shown beneficial biological and pharmacological properties for the regeneration of bone, blood vessels, and soft tissue (Kim et al. 2018). When used with biomaterials, it provides antimicrobial and anti-immunologic benefits (Kweon et al. 2014) and it supports new bone formation when incorporated into bone substitute materials. Additionally, 4-HR promotes vascular endothelial growth factor production and endothelium regeneration. By inhibiting the foreign-body response, it can also improve the success rate of biomaterial implants in the body (Kim and Seok 2020). 4-HR is known to have antiparasitic and antimicrobial activities (Kozubek and Tyman 1999), has angiogenic effect by increasing vascular endothelial growth factor (Katagiri et al. 2017). While 4-HR is known to exhibit some actions similar to the female hormone estrogen, recent research suggests that it does not alter estrogen receptor expression or exert an estrogen-like effect in a rat model (Kim and Seok 2020).

Consumers often favor natural antioxidants, as they are perceived to be safer and tend to gain regulatory approval more readily than synthetic additives. However, the presence of a compound in food does not inherently guarantee its complete safety (Matsuo and Kaneko 1999). While synthetic antioxidants undergo extensive testing for carcinogenic and mutagenic effects, many natural food compounds have not been subjected to similar scrutiny. The pros and cons of synthetic and natural antioxidants are outlined in Table 2 (Ames 1996; Gülçin 2012; Esazadeh et al. 2024). Despite potential risks, the advantages of incorporating antioxidants outweigh their drawbacks. In the absence of antioxidants, oxidation products form in foods, posing a greater threat to health than the potential adverse effects of antioxidants themselves (Gülçin 2020).

**Table 2:** Advantages and disadvantages of natural and synthetic antioxidants commonly used for food protections

Synthetic antioxidants	Natural antioxidants
• Antioxidant capacity ranges from moderate to high	• Diverse and extensive antioxidant effects
• Don't offer a variety of products	• Offer a variety of products
• Don’t exhibit antioxidant effects on human tissues	• Exhibit antioxidant effects on human tissues
• They can be used as coloring or flavoring agents	• They are obtained from natural sources
• Limited solubility in water	• Diverse solubility profiles
• Inexpensive	• Expensive
• Widely applied	• Usage of some products restricted
• Medium to high antioxidant activity	• Wide ranging antioxidant activity
• Increasing safety concern	• Perceived as innocuous substances
• Usage of some of them banned	• Increasing usage and expanding applications
• Low water-solubility	• Broad range of solubility
• Decreasing interest	• Increasing interest
• Some of them stored in adipose tissues	• Completely metabolized

### Prooxidants

A prooxidant is a substance that promotes oxidative stress within cells and tissues. There is no doubt that these molecules play a vital role in metabolic pathways and protect cells from ROS and oxidative stress. However, recent studies have forced the academic community to examine and investigate the roles of antioxidants and prooxidants more deeply. In recent years, antioxidants and prooxidants have been studied extensively, and it appears that most dietary antioxidants can act as prooxidants; this depends on their concentration and the nature of the surrounding molecules (Puglia and Powell 1984; Carocho and Ferreira 2013). Free radicals are considered prooxidants, but surprisingly, antioxidants can also exhibit prooxidant effects under different conditions and at high concentrations. It usually does this by generating ROS or RNS, which can damage cellular components like proteins, lipids, and DNA. This oxidative stress can lead to cell and tissue damage if not properly regulated. Prooxidants can come from both external sources, such as pollutants, radiation, and certain drugs, and internal sources, like metabolic byproducts. Interestingly, some molecules known for their antioxidant properties, like vitamin C and flavonoids, can act as prooxidants under certain conditions, especially at high concentrations or in the presence of transition metals like iron or copper. While prooxidants are generally associated with cell damage, they can also play a beneficial role. In controlled amounts, prooxidants can signal cells to activate defense mechanisms and repair processes, contributing to cellular resilience and adaptation (Puglia and Powell 1984). Vitamin C is widely regarded as a potent antioxidant, involved in numerous physiological processes, but under certain conditions, it can also act as a prooxidant. This occurs when it interacts with iron or copper, reducing Fe^3+^ to Fe^2+^ or Cu^2+^ to Cu^+^, which then facilitates the reduction of H_2_O_2_ to reactive hydroxyl radicals (Duarte and Lunec 2005). Similarly, α-tocopherol is recognized as a powerful antioxidant, but at high concentrations, it may also act as a prooxidant due to its antioxidant mechanism. When it reacts with a free radical, it becomes a radical itself. If not enough ascorbic acid is available to regenerate it, the tocopherol radical remains in this highly reactive form, which can lead to the autoxidation of linoleic acid (Cillard et al. 1980). When reviewing the literature on antioxidants, it has been stated that antioxidants have evolved from “Miracle Molecules” to “Marvelous Molecules” and finally to “Physiological Molecules” (Singh et al. 2010a, b). There is no doubt that these molecules are essential in metabolic pathways and protect cells; however, recent contradictory evidence has led the academic community to investigate the roles of antioxidants and prooxidants more closely. Prooxidants are defined as substances that induce oxidative stress, often by generating reactive species or by inhibiting antioxidant systems (Puglia and Powell 1984). Free radicals are considered prooxidants, but interestingly, antioxidants themselves can sometimes exhibit prooxidant behavior.

Although evidence is limited, carotenoids are also believed to have prooxidant properties, particularly through autoxidation when exposed to high oxygen concentrations, generating hydroxyl radicals (Young and Lowe 2001). Even flavonoids can act as prooxidants, though their behavior depends on the specific environmental conditions. Additionally, dietary phenolics can become prooxidants in systems containing redox-active metals. The presence of oxygen and transition metals, such as iron and copper, can catalyze the redox cycling of phenolics, potentially leading to the formation of ROS and phenoxyl radicals, which can damage DNA, lipids, and other biological molecules (Galati and O’Brien 2004). Also, Yordi et al. (2012) compiled a list of 14 phenolic acids, which, although generally considered antioxidants, may act as prooxidants under certain circumstances (Carocho and Ferreira 2013).

In addition to their pro-oxidant effects, antioxidants have their own range of efficacy at different doses. However, some may cause adverse effects beyond a certain level of intake. Therefore, the selection of the type and dose of antioxidants becomes an important criterion. Both in vivo and in vitro studies suggest that excessive intake of natural antioxidants and their supplements may have negative effects on women's reproductive health. The safety of high doses of vitamins has always been a subject of concern (Bhardwaj et al. 2021). From this perspective, the uncontrolled and unconscious use of antioxidants can lead to undesirable outcomes, similar to pro-oxidants. Therefore, the safety of high doses of antioxidants has always been questioned.

#### Natural antioxidants

Recently, concerns have arisen regarding the potential toxicity and undesirable effects of synthetic antioxidants. In response, interest has grown in exploring plant-derived products as natural antioxidants that can protect against ROS and diseases caused by free radicals (Hou et al. 2003). This trend has encouraged food manufacturers to increasingly consider replacing synthetic antioxidants with natural ingredients that contain antioxidant compounds. Consequently, research into natural food additives has accelerated, as they are generally regarded as safe for consumers (Shahidi & Wanasundara 1995; Shahidi & Ambigaipalan 2015). Natural antioxidant sources are predominantly plant phenolics, which are found throughout plant structures such as fruits, vegetables, seeds, nuts, leaves, roots, flours, and bark. Plants naturally produce a wide array of secondary metabolites, including flavonoids, essential oils, alkaloids, lignans, terpenes, tocopherols, phenolic acids, and more, as part of their metabolic processes. These compounds play a crucial role in protecting plants from harmful effects (Shahidi & Ambigaipalan 2015; Martelli and Giacomini 2018).

The human diet is also rich in various antioxidant compounds capable of scavenging ROS, thanks to their structural characteristics. Notable examples of dietary antioxidants include vitamin C, tocopherols, carotenoids, and flavonoids. Apart from vitamin C, each of these antioxidant groups includes numerous structurally distinct compounds. For instance, over 600 carotenoids have been identified, with about 50 commonly occurring in human diets (Sies and Stahl 1995; Rice-Evans and Miller, 1996). The interplay of these compounds in the diet can produce synergistic effects that are not easily measured, suggesting that diet operates like an orchestra, where interactions between components lead to effects not attributable to individual elements alone (Diplock et al. 1998).

### Phenolic compounds

**Fig. 7:**
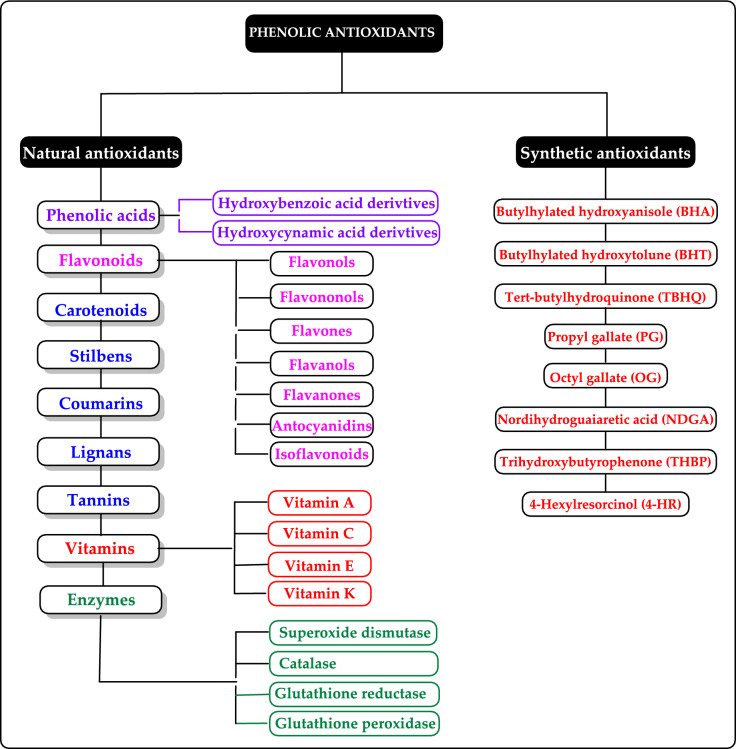
Classification of phenolic antioxidant compounds

Phenolic compounds, as natural antioxidants, exhibit considerable structural diversity and chemical composition variations across plant-derived substances. These compounds are ubiquitous, present in nearly all plants, microorganisms, fungi, and even animal tissues (Pokorny 1999; Kassa et al. 2022). Recognized for their multifunctional bioactivity, phenolic compounds are extensively distributed throughout the plant kingdom. Many of these compounds are integral to the human diet and are also used in medicinal applications. The health benefits of phenolic compounds are often attributed to their antioxidant, anticarcinogenic, antimutagenic, antimicrobial, anti-inflammatory, and various other biological properties. Common plant-based phenolic compounds include phenolic acids, flavonoids, tannins, lignans, and terpenes (Nazck and Shahidi 2006; Shahidi and Ambigaipalan 2015). As secondary metabolites, phenolic compounds are naturally found in nearly all plant materials, including food products of plant origin, making them essential components of both human and animal diets (Gülçin, 2006b; Taslimi and Gulçin 2017). Among natural antioxidants, phenolic compounds are predominant, with the most significant groups being tocopherols, flavonoids, and phenolic acids (Fig. 7).

### Phenolic acids

Phenolic acids are abundant in nearly all plants and plant-based foods, making up a significant part of the human diet. Average daily intake of phenolic acids through diet is estimated to be around 200 mg, though this can vary based on individual dietary preferences and habits (Clifford and Scalbert 2000). Interest in phenolic acids has recently increased due to their potential protective effects, achieved through the consumption of fruits and vegetables, against oxidative damage-related diseases such as coronary heart disease, stroke, and certain cancers (Gülçin et al. 2010a), as well as their antiglaucoma (Çoban et al. 2007a; Öztürk Sarıkaya et al. 2010; 2011; Innocenti et al. 2010a, 2010b; Şenturk et al. 2011) anticholinergic (Gül et al. 2019; Taslimi et al. 2019; Tohma et al. 2019) and antidiabetic (Demir et al. 2019; Çağlayan et al. 2019) properties. Phenolic acids make up approximately 30% of the dietary phenolics in plants, found in both free and bound forms (Robbins 2003). The bound form is more common, typically occurring as esters, glycosides, and insoluble-bound complexes (Ross et al. 2009). They are hydroxy derivatives of aromatic carboxylic acids derived either from benzoic acid or cinnamic acid groups. Widely distributed in the plant kingdom, common phenolic acids include p-hydroxybenzoic acid, 3,4-dihydroxybenzoic acid, vanillic acid, syringic acid, p-coumaric acid, caffeic acid, ferulic acid, sinapic acid, chlorogenic acid, and rosmarinic acid (Oztürk Sarikaya et al. 2010). Rosmarinic acid is an ester of caffeic acid and 3,4-dihydroxyphenyl lactic acid. It has a potent antioxidant and a powerful inhibitor of certain metabolic enzymes such as carbonic anhydrase, glutathione S-transferase, lactoperoxidase, acetylcholinesterase, and butyrylcholinesterase, (Topal and Gülçin 2014; Gulcin et al. 2016). Cinnamic acid derivatives are generally more potent antioxidants compared to benzoic acid derivatives (Chen and Ho 1997).

**Fig. 8:**
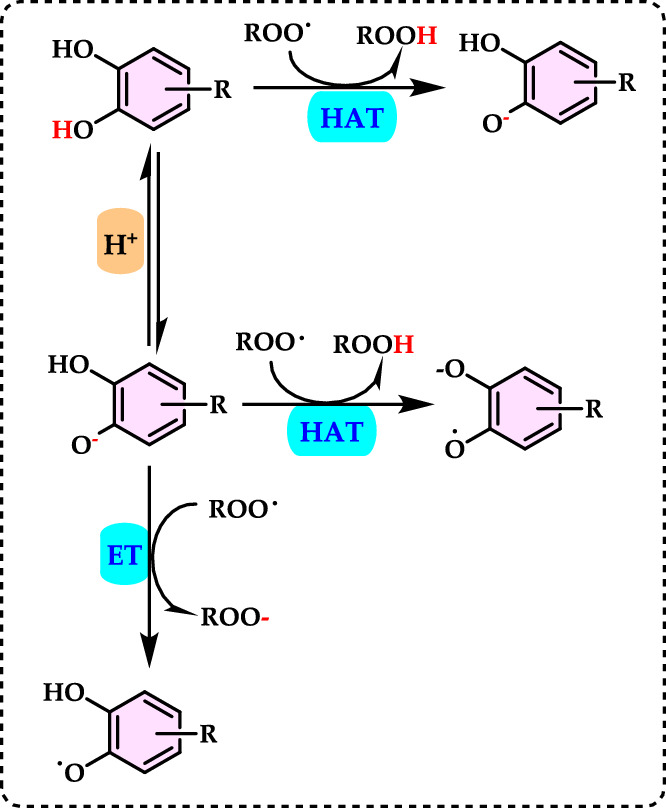
The interaction between catechol (1,2-dihydroxybenzene) and peroxyl radicals (ROO·) unfolds through a sequence of electron transfer (ET) and hydrogen atom transfer (HAT) steps

The effectiveness of antioxidant compounds like phenolics is influenced by various factors, including their structural properties, temperature, the characteristics of the oxidizable substrate, concentration, and the presence of synergistic or pro-oxidant compounds, as well as the physical state of the system (Huyut et al. 2017b). The chemical structure of an antioxidant plays a crucial role in determining its reactivity with free radicals and other ROS, thereby impacting its antioxidant performance. Additionally, the efficiency of antioxidants is affected by their concentration and distribution within the system, such as their localization at interfaces (Shahidi and Zhong 2011). Also, the antioxidant activity of phenolic acids and derivatives is influenced by several structural factors: the number and position of hydroxyl groups on the aromatic ring, the specific binding site and relative positions of these hydroxyl groups, as well as the type of substituents present (Rice-Evans et al. 1996; Sroka and Cisowski 2003; Gulcin 2012). They are typically divided into two main groups: benzoic acids, which contain seven carbon atoms (C6–C1), and cinnamic acids, which consist of nine carbon atoms (C6–C3). Both phenolic acids and derivatives (Fig. 8) are naturally occurring bioactive compounds that are synthesized in plants through the shikimate pathway, with phenylalanine and tyrosine serving as the precursor molecules. Among various biological activities, cinnamic acid and its derivatives are associated with a beneficial influence on diabetes and its complications (Adisakwattana 2017). As shown in Table 3, phenolic acids are primarily divided into two groups: hydroxybenzoic acids and hydroxycinnamic acids. Hydroxycinnamic acids have been observed to exhibit significantly higher antioxidant activity compared to hydroxybenzoic acids. This is attributed to the conjugated structure in hydroxycinnamic acids, where double bonds in the aromatic ring extend through the −CH=CH–COOH group in the cinnamic acid structure, thus enhancing free radical stabilization. The presence of a –CH_2_=CH–COOH group in cinnamic acids provides a greater antioxidant capacity than the -COOH group found in benzoic acids (Cuppett et al. 1997; Gülçin 2020).

**Table 3:**
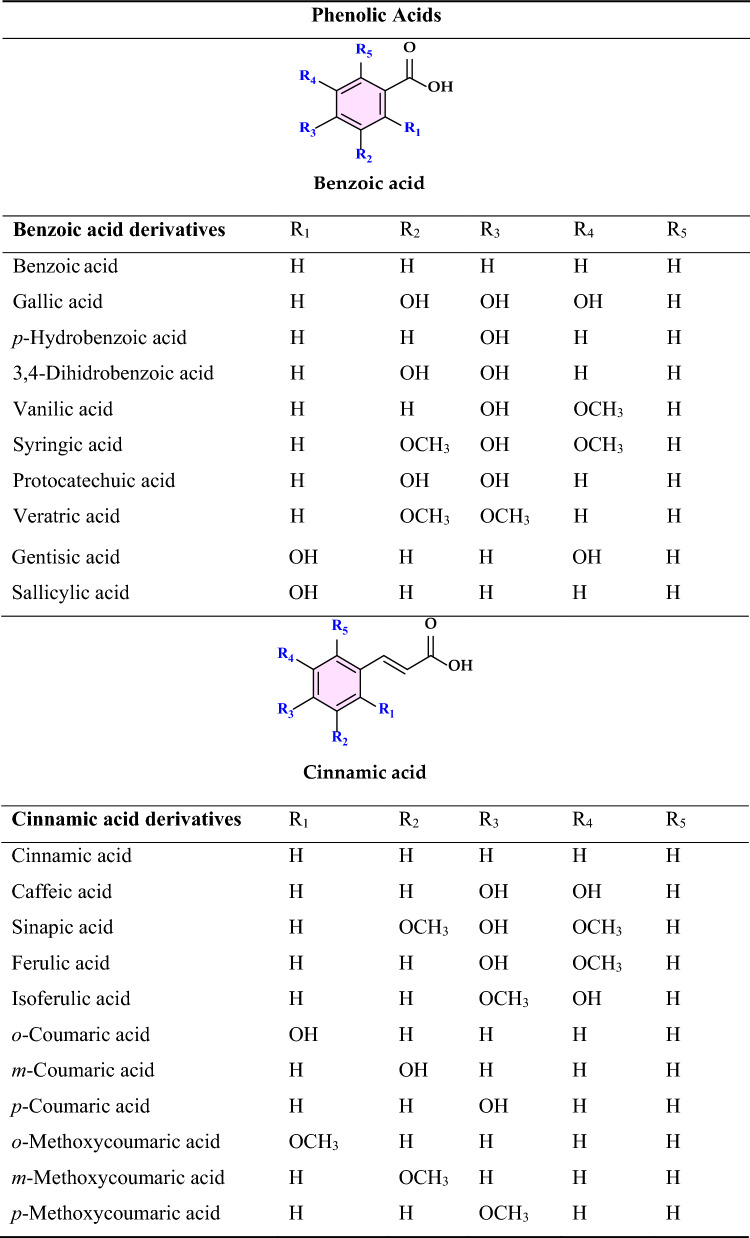
Chemical structures of naturally occurring phenolic acids and related compounds as benzoic acid and cinnamic acid derivatives

Substituents on the phenolic backbone also play a crucial role in modulating antioxidant properties, especially their ability to donate hydrogen. Generally, unsubstituted phenol is inactive as a hydrogen donor, and monophenols are less efficient antioxidants than polyphenols. Adding electron-donating groups, such as hydroxyl groups in the ortho- or para- positions, increases the antioxidant activity of phenols or phenolic acids (Chimi et al. 1991). Despite having the same number of –OH groups, catechin shows significantly lower antioxidant activity than quercetin. This is because catechin lacks unsaturated bonds at the C2–C3 position and the oxo (−C=O) group on the C ring, which contributes to quercetin’s comparatively higher antioxidant activity. When an –OH group is added to the B ring of catechin, forming epigallocatechin, the antioxidant activity increases, though it still does not reach quercetin’s level (Rice-Evans et al. 1996).

Furthermore, the presence of carbonyl groups, such as aromatic acids, esters, or lactones, enhances antioxidant activity, especially when the carbonyl group is separated from the aromatic ring. This structural feature is one reason why cinnamic acids exhibit stronger antioxidant activity than their corresponding benzoic acids. Additionally, steric hindrance caused by neighboring inert groups, like methoxy groups, can increase the antioxidant activity of phenolic hydroxyl groups (Dziedzic and Hudson 1984; Gocer and Gülçin 2011). The antioxidant activity of phenolic compounds is also largely determined by their structure, particularly the position and number of -OH groups and the type of substitutions on the aromatic rings (Cosme et al. 2018). It is worth noting that gallic acid exhibits stronger antioxidant activity than catechin, even though catechin contains five −OH groups (Rice-Evans et al. 1996).

Cinnamic acids are known for a variety of biological effects, including free radical scavenging, antioxidant activity, UV protection, antiviral, and antibacterial properties. These compounds and derivatives are active components in many plant-based foods, with broad availability in grains, legumes, oils, seeds, fruits, and vegetables (Martelli and Giacomini 2018). The primary hydroxycinnamic acids, such as coumaric, caffeic, ferulic, and sinapic acids, demonstrate antioxidant potential in vitro, suggesting possible health benefits when functioning in vivo (Kroon and Williamson 1999; Gulcin 2012). Interestingly, the antioxidant effectiveness of phenolic acids is inversely related to their O–H bond dissociation enthalpy values, which indicates that in low-polarity organic environments, the key antioxidant mechanism involves hydrogen atom transfer (HAT) from the phenolic OH to ROO· radicals (Fig. 8).

The position and degree of hydroxylation are crucial factors that determine the antioxidant activity of compounds (Dziedzic and Hudson 1984; Chen and Ho 1997). Numerous studies have demonstrated a clear relationship between the structure of phenolic acids and their antioxidant potential (Chen and Ho 1997; Goçer and Gülçin 2011). Monophenols are generally less effective than diphenols or polyphenols in terms of antioxidant activity. Recent studies have shown that l-Dopa exhibits higher antioxidant effect and radical scavenging capabilities compared to l-Tyrosine. This suggests that the antioxidant activity of these compounds is strongly influenced by the number of hydroxyl groups present (Gülçin 2007). The addition of a second hydroxyl group in the ortho- or para-position significantly enhances antioxidant activity. Furthermore, monophenols with one or two methoxy substitutions show increased inhibitory effectiveness. The combination of two phenolic acids also boosts antioxidant activity; for example, rosmarinic acid is a more potent antioxidant than caffeic acid. Caffeic acid often occurs as esters, with chlorogenic acid being the most common form. The antioxidant properties of caffeic acid have been well established, and its antioxidant mechanism has been clarified (Gülçin 2006b).

Esterification of caffeic acid with a sugar moiety, as seen in chlorogenic acid, reduces its activity (Chen and Ho 1997). Caffeic acid is one of the major hydroxycinnamic acids. Pekkarinen et al. (1999b) studied the impact of hydroxybenzoic and hydroxycinnamic acids on the oxidation of methyl linoleate, both in bulk and emulsified forms, and found that interactions between antioxidants and other compounds, such as emulsifiers, as well as intramolecular hydrogen bonding, are crucial in determining antioxidant efficiency. Oleuropein, tyrosol, and hydroxytyrosol are significant phenolic compounds found in olive oil (Gülçin et al. 2006a; Gülçin 2012), and it has been shown that hydroxytyrosol and caffeic acid provide greater antioxidant protection than BHT (Papadopoulos and Boskou 1991; Gülçin 2006b). Sesame oil, a popular edible oil derived from sesame seeds, also contains several natural antioxidants, including sesamol (Budowski et al. 1950). Sesame oil is also employed in alternative medicine for massages and various treatments (Gülçin 2012).

Antioxidants can behave differently depending on the type of radical or oxidant they are exposed to. For instance, while carotenoids are not especially effective at scavenging ROO· radicals compared to phenolics and other antioxidants, they are highly efficient at quenching singlet oxygen-something that many other phenolic compounds and antioxidants struggle with (Prior et al. 2005; Gülçin et al. 2012a; 2012b).

### Flavonoids

Flavonoids are a group of cyclized diphenyl propanes and widely present in plants, especially in plant-based foods. These compounds are polyphenolic in nature and serve as highly potent antioxidants, playing a significant role in the prevention of chronic diseases. More than 8000 polyphenolic compounds have been identified, with over 4000 classified as flavonoids, found across various plant parts including leaves, stems, roots, fruits, and seeds (Harborne et al. 1999). Flavonoid biosynthesis in plants begins with the aromatic amino acids phenylalanine and tyrosine, along with malonate. Flavonoid biosynthesis is a complex biochemical process in plants, leading to the production of a diverse group of compounds that serve important roles in plant defense, pigmentation, and signaling. This pathway, part of the larger phenylpropanoid pathway, primarily uses the amino acids phenylalanine and tyrosine, along with malonyl-CoA, as starting materials (Harborne 1986). This process produces a diverse group of flavonoids, such as flavones, flavanols, isoflavones, flavanones, and chalcones, which are prevalent in many plant tissues. Among these, flavanones undergo transformations that alter the structure of the central heterocyclic ring, eventually leading to the formation of compounds like anthocyanins and catechins. Flavonoid structures exhibit variation primarily around the oxygen-containing heterocyclic ring but share a common C6–C3–C6 carbon skeleton (Table 4). The fundamental structure of flavonoids is based on the 2-phenylchromone core, which contains three rings (A, B, and C) with variable methoxylation and hydroxylation patterns (Shahidi and Ambigaipalan 2015). The flavonoid core consists of 15 carbon atoms arranged in a C6–C3–C6 pattern, forming a structure known as the flavan nucleus. The presence of double bonds, a carbonyl group, and hydroxyl groups within the C ring serves as the basis for their classification into various subclasses. The A and B rings often contain hydroxyl substituents, which further differentiate individual members within each flavonoid class (Musialik et al. 2009). All flavonoids share this diphenylpropane (C6–C3–C6) backbone, although their chemical properties and activities vary depending on their specific structures and the spatial arrangement of functional groups (Shahidi and Ambigaipalan 2015). The oxidation level and substitution patterns on the C ring determine the flavonoid classes, while individual compounds within each class differ based on the substitution pattern of the A and B rings. In general, the flavonoid structure comprises two aromatic rings connected by a three-carbon bridge, typically forming a condensed pyran ring, though occasionally a furan ring may form instead (Harborne 1986; Gulcin 2012).

**Table 4:**
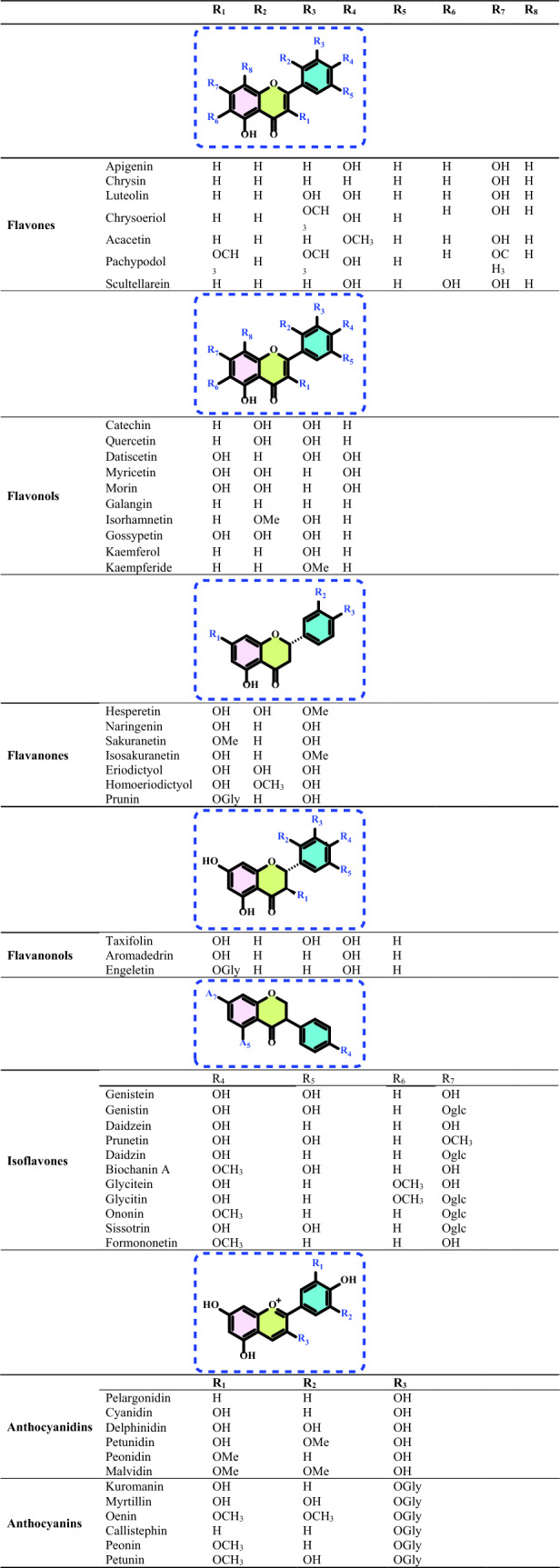
Chemical structure of naturally occurring flavonoids

Flavonoids are a diverse group of polyphenolic natural compounds abundantly found in plant-based foods. They exhibit various substitution patterns on their two benzene rings (A and B), contributing to their structural diversity in nature (Ghosh et al. 2015). Renowned for their potent antioxidant properties, flavonoids are thought to protect against cardiovascular diseases by inhibiting the oxidation of LDL. As significant dietary antioxidants, flavonoids are primarily obtained from fruits, vegetables, and plant-derived beverages like tea. The average daily intake of flavonoids is estimated to be several hundred milligrams. To date, more than 4000 naturally occurring flavonoids have been identified (Ghosh et al. 2015). Despite their abundance in food, flavonoids are generally poorly absorbed in the human digestive system, and their overall impact on plasma antioxidant capacity is not yet fully understood (Formica and Regelson 1995). Nonetheless, flavonoids are associated with numerous health benefits, including protection against oxidative stress-related diseases, the ability to modulate enzyme activity, and interactions with specific cellular receptors (Williams and Spencer 2004). The antioxidant effectiveness of flavonoids is influenced by several factors: their metal-chelating capacity, which is determined by the arrangement of hydroxyl and carbonyl groups within the molecule; the presence of hydrogen- or electron-donating groups that neutralize free radicals; and the ability to stabilize unpaired electrons through the formation of stable phenoxy radicals. These properties collectively underscore the critical role flavonoids play in maintaining health and preventing oxidative damage (Gulcin et al. 2011a; Gulcin 2012).

**Fig. 9:**
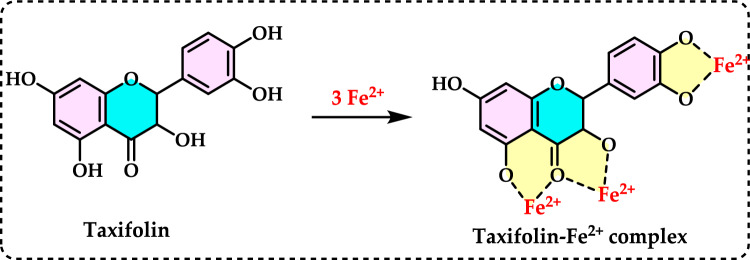
Schematic representation of metal binding sites for taxifolin as flavonoids

Metal chelation is a significant mechanism through which flavonoids, including flavonols, exert their antioxidant effects. The interaction of metal ions with flavonoids can alter their antioxidant capabilities and biological activities. Studies suggest that when flavonoids coordinate with suitable metal ions, their biological activity can be enhanced due to their ability to act as free radical acceptors (Gulcin et al. 2010c; Ghosh et al. 2015). For example, taxifolin, a type of flavanonol, demonstrates strong metal-binding capabilities. As shown in Fig. 9, taxifolin binds metal ions, such as Fe^2^⁺, at specific sites: the catechol group on ring B, the 3-hydroxyl and 4-oxo groups on the heterocyclic ring, and the 4-oxo and 5-hydroxyl groups between the heterocyclic and A rings. Its ability to inhibit lipid peroxidation is primarily linked to its Fe^2^⁺-binding activity. Taxifolin prevents the formation of the ferrous ion-ferrozine complex, effectively sequestering Fe^2^⁺ ions before ferrozine can bind. This activity may involve forming insoluble metal complexes or creating steric hindrance that inhibits interactions between metal ions and lipid intermediates. It is proposed that taxifolin can chelate up to three ferrous ions through its hydroxyl and carbonyl groups (Topal et al. 2016b). Metal stabilization of semiquinone-metal complexes further supports this mechanism. The catechol group, as seen in quercetin, plays a dominant role in metal chelation, with stronger effects compared to other flavonols like kaempferol, as evidenced by a more pronounced bathochromic shift upon copper binding (van Acker et al. 1996). Flavonoids also play a crucial role in regulating the bioavailability of trace metals like aluminum and assist in detoxifying heavy metals such as chromium, cadmium, tin, and lead. By forming stable complexes with toxic metal ions, chelating agents facilitate their elimination from the body (Dehghan and Khoshkam 2012; Ghosh et al. 2015).

The stability of radicals generated from flavonoid structures is primarily influenced by hydrogen bonding between hydroxyl groups and oxygen atoms containing unpaired electrons. The presence of a C=C double bond in the C ring, conjugated with a 4-oxo group, plays a critical role in the delocalization of unpaired electrons. Additionally, the presence of hydroxyl groups at the 3- and 5-positions enhances antioxidant activity. These positions are kinetically equivalent due to an internal hydrogen bond with the 4-oxo group (Bors et al. 1990). To analyze the impact of the number of hydroxyl groups on activity, a comparison was conducted among compounds with one or more hydroxyl groups substituted on a single aromatic ring. Compounds with two or more hydroxyl groups in the same orientation showed higher TEAC values compared to those with only one. The hydroxyl group is an electron-donating group, which might be the primary factor in facilitating the reaction. However, hydroxylated compounds demonstrated higher TEAC values than those with a methoxy group (considered a stronger electron donor) in the same position (Yamauchi et al. 2024). Several methods exist for assessing antioxidant activity, broadly divided into two categories. The first involves evaluating the antioxidant’s ability to inhibit oxidation in a model system, with changes monitored through physical, chemical, or instrumental techniques. Radical scavenging assays are commonly used, relying on hydrogen atom transfer (HAT) or single electron transfer (SET) mechanisms (Shahidi and Ambigaipalan 2015). Phenolic antioxidants (commonly Ar-OH) typically act via two main mechanisms: HAT or SET followed by proton transfer (SET-PT) (Wright et al. 2001). However, these mechanisms sometimes overlap and are not always distinctly separate (Apak et al. 2022). HAT-based methods evaluate an antioxidant’s ability to neutralize free radicals by donating hydrogen atoms. Conversely, SET-based methods focus on the antioxidant’s capacity to transfer a single electron, reducing compounds such as metals, carbonyls, or radicals (Shahidi and Ambigaipalan 2015).

$${\text{ArOH}} \to {\text{ ArO}}^{ \bullet } + {\text{H}}^{ \bullet } \left( {{\text{HAT}}} \right)$$

$${\text{ArOH}} \to {\text{ ArO}}^{ \bullet + } + {\text{e}}^{ - } \left( {{\text{SET}} - {\text{PT}}} \right)$$

$${\text{ArO}}^{ \bullet + } \to {\text{ ArO}}^{ \bullet } + {\text{H}}^{ + }$$

Recently, a new mechanism has been proposed, known as the sequential proton loss electron transfer (SPLET) mechanism.

$${\text{ArOH}} \to {\text{ ArO}}^{ - } + {\text{H}}^{ + }$$

$${\text{ArO}}^{ - } + {\text{ ROO}}^{ \bullet } \to {\text{ArO}}^{ \bullet } + {\text{e}}^{ - }$$

The 7-OH group in flavonoids is crucial as a site for ionization and electron transfer within the framework of the sequential proton loss electron transfer (SPLET) mechanism. This mechanism, which was identified more recently (Litwinienko and Ingold 2004; Foti et al. 2004a, b), occurs in two distinct steps:

$${\text{ArOH}} \to {\text{ArO}}^{ - } + {\text{H}}^{ + }$$

$${\text{ArOH}} + {\text{ ROO}}^{ \bullet } \to {\text{ArO}}^{ \bullet } + {\text{ROO}}^{ - }$$

**Fig. 10:**
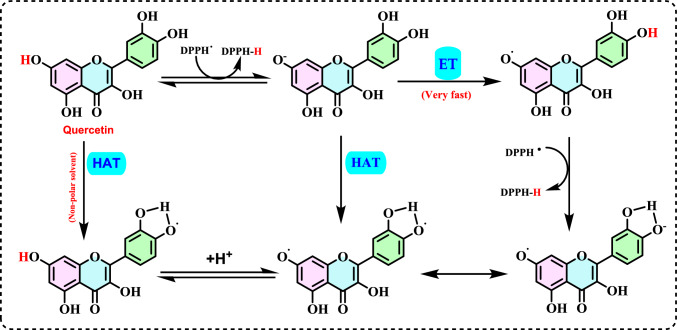
Potential mechanisms for the reaction of quercetin, as a flavonoid, with DPPH radicals

**Fig. 11:**
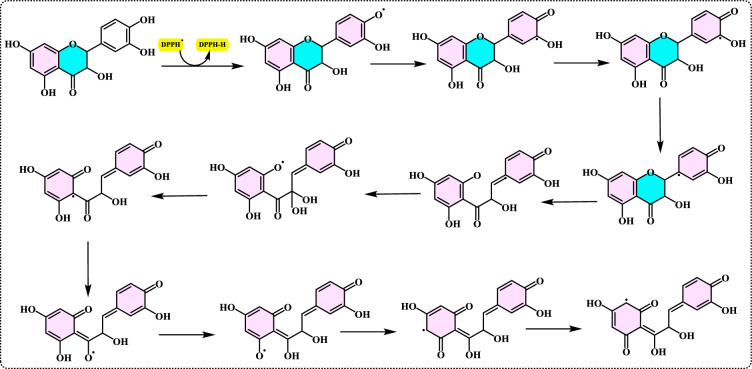
The suggested reaction pathway between DPPH free radicals and taxifolin

The reaction enthalpy of the first step in the SPLET mechanism corresponds to the proton affinity of the phenoxide anion (ArO⁻) (Vianello and Maksic 2006). In the second step, an electron transfer occurs from the phenoxide anion to the ROO· radical, resulting in the formation of a phenoxy radical. The reaction enthalpy of this step is referred to as the electron transfer enthalpy. From the perspective of antioxidant activity, the overall effect of the SPLET mechanism is equivalent to that of the HAT mechanism in neutralizing free radicals. For instance, quercetin, a well-known bioflavonoid, exhibits significant antioxidant and radical-scavenging properties. The proposed mechanisms for the reactions of quercetin and taxifolin with DPPH· radicals are illustrated in Figs. 10 and 11. Both flavonoids effectively reduce the stable DPPH radical to its yellow-colored DPPH-H form. The molecule of DPPH is classified as a stable free radical due to the delocalization of its unpaired electron across the entire molecule. This delocalization prevents the molecule from dimerizing, which is typical behavior for most other free radicals. The delocalization of the electron also results in the molecule's deep violet color, characterized by an absorption band in ethanol solution at approximately 517 nm. When a DPPH solution is combined with a substrate capable of donating a hydrogen atom, the molecule is reduced, leading to the disappearance of its violet color. To assess the antioxidant potential of the test samples through free radical scavenging, the change in the optical density of DPPH radicals is measured (Alam et al. 2013a, b). Furthermore, taxifolin, a naturally bioactive flavonoid, has been reported to exhibit notable inhibitory effects on certain metabolic enzymes, including human carbonic anhydrase I and II isoenzymes, as well as the acetylcholinesterase enzyme (Gocer et al. 2016).

**Fig. 12:**
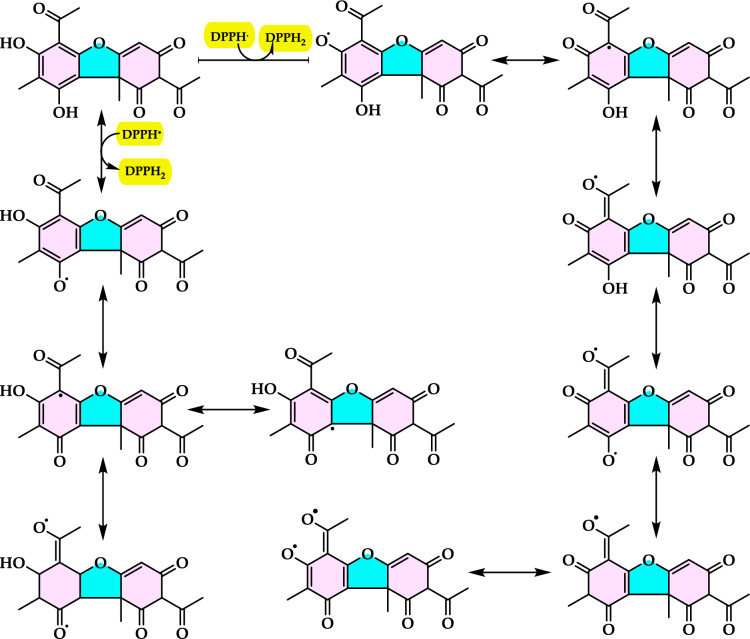
Purposed radical scavenging mechanism between usnic acid and DPPH radicals

Similarly, the structure of usnic acid allows it to interact with DPPH· radicals. Upon interaction, DPPH· is reduced to DPPH₂ after accepting an electron or hydrogen atom from usnic acid. The specific reaction between DPPH· and usnic acid is depicted in Fig. 12. In the usnic acid molecule, the phenolic group contains two hydroxyl units, which facilitates the removal of hydrogen atoms from these phenolic hydroxyl groups. Usnic acid can adopt triradical structures by neutralizing three DPPH molecules through resonance stabilization (Cetin Cakmak and Gulcin 2019).

These reactions are significantly accelerated by an increase in electron density in rings A and C. Additionally, an alternative pathway involves a rapid electron transfer (ET) from the phenolate anion to DPPH radicals. Ring A exhibits strong electron-withdrawing properties, and due to conjugation effects, the catechol moiety in ring B is the most likely site for deprotonation. Owing to the presence of –OH groups, many flavonoids are primarily located in the aqueous phase of biological systems. Flavonoids' reactions with electron-deficient radicals can be enhanced through the SPLET mechanism, effectively reducing the accumulation of reactive oxygen species in cells (Musialik et al. 2009). Since SET-PT and SPLET mechanisms are crucial in solvated environments, it is essential to investigate how water influences the reaction enthalpies of individual steps in these mechanisms (Klein et al. 2007). Moreover, Litwinienko and Ingold proposed a modified SPLET mechanism in 2004, particularly relevant in solvents that facilitate ionization, such as methanol among organic solvents (Litwinienko and Ingold 2003). This mechanism is more favorable for reactions involving phenols with low pKa values and electron-deficient radicals with relatively low HAT activity, producing products with similarly low pKa values (Litwinienko and Ingold 2004). The occurrence of SPLET in methanol and ethanol was further demonstrated by Foti et al. (2004a, b). Their study highlighted the reactions of DPPH· with phenolic acids such as caffeic acid, p-coumaric acid, ferulic acid, and sinapic acid. They observed that the reaction rate constants for the methyl esters of these acids were significantly higher than those for the free acids. This difference was attributed to the suppression of ionization of the phenolic –OH group by the free carboxylic acid group. These findings underscore the importance of phenol ionization in the reactions of phenols with DPPH· in solvents that promote ionization.

Flavonoids, including flavones, flavonols, isoflavones, flavanones, and chalcones, are found in various types of higher plant tissues (White and Xing 1997). Flavones and flavonols are present in nearly all plants, especially in the leaves and petals, with flavonols being more common than flavones (Table 4). Some common flavonoids include apigenin, chrysin, luteolin, datiscetin, myricetin, quercetin, kaempferol, and morin. Additionally, most flavonoids in plants occur as glycosides (Macheix et al. 1990). The ability of flavonoids to inhibit lipid peroxidation is well established, both for natural lipid products and model lipids (Bors et al. 1996). Flavonoids are known to act as antioxidants by scavenging radicals such as lipid peroxyl radicals (Takahama 1985), superoxide anion radicals (Hu et al. 1995), hydroxyl radicals (Husain et al. 1987), singlet oxygen (Takahama 1984), and by chelating metal ions (Ramanathan and Das 1993). For maximal radical scavenging activity, a flavonoid molecule must meet several structural criteria: a 3',4'-dihydroxy structure in the B-ring, a 2,3-double bond along with a 4-oxo group in the C-ring, and hydroxyl groups at the 3 and 5 positions on the A-ring (Hu and Ding 1996). Flavonoids with free hydroxyl groups act as free-radical scavengers, and multiple hydroxyl groups, particularly in the B-ring, enhance their antioxidant activity. The hydroxyl groups in the B-ring are the primary active sites in interrupting the oxidation chain (Jovanovic et al. 1994).

Flavonoids are among the most common and popular polyphenolic compounds in the human diet and are widespread in plants. They can help prevent coronary heart disease and possess antioxidant properties (Gülçin et al. 2011a, b; Sehitoglu et al. 2015). The antioxidant and biological activities of flavonoids depend on their chemical structure. Three key structural features determine their free radical scavenging and antioxidant activities: the catechol group in the B-ring, the 2,3-double bond conjugated with a 4-oxo group in the C-ring, and the presence of hydroxyl groups at the 3 and 5 positions. Quercetin contains all three of these features, which typically gives it higher antioxidant potential compared to kaempferol, which lacks the catechol group in the B-ring. The presence of a hydroxyl group at the 5' position in the B-ring further enhances the antioxidant potential significantly. Quercetin and kaempferol are flavonoids that are widely found in nature. It has been reported that quercetin is significantly more effective than kaempferol in slowing down the autoxidation of lard (Yanishlieva et al. 1984) and methyl linoleate (Pekkarinen et al. 1999a). The autoxidation of oils is a critical factor determining their shelf-life, leading to the formation of free fatty radicals, peroxides and hydroperoxides. These reactions result in decreased oil quality and the development of off-flavors and off-odors. Autoxidation is affected by the oil's composition and storage conditions, including free fatty acid content, light exposure, and temperature. It can be slowed down or even completely prevented by the addition of antioxidants (Platzer et al. 2022). The relative reactivities and stoichiometric coefficients of various flavonoids, catechols, and standard phenolic antioxidants have been determined (Roginsky et al. 1996). Furthermore, the kinetics of oxygen consumption in both organic and micellar systems, with peroxidation initiated by lipid or water-soluble initiators, were analyzed. Roginsky et al. (1996) showed that flavonoids do not act like traditional phenolic antioxidants, such as α-tocopherol, but exhibit only moderate chain-breaking activity. The antioxidant activity of flavonoids is highly influenced by the substrate system and the conditions used to catalyze oxidation. The structure–activity relationship for the antioxidant activities of flavonoids has been extensively studied and is well established (Foti et al. 1996; Chen et al. 1996).

*Flavones* constitute one of the largest subcategories within the flavonoid family. Two distinct enzymes, which evolved independently and function through different mechanisms, are capable of converting flavanones, their precursors, into flavones. The diverse biological activities of flavones in plants, as well as their significance in human nutrition and health, make them important targets for metabolic engineering. Structurally, flavones resemble the flavonols but differ by the less hydroxyl group at C3 of the pyran ring (De Souza Farias et al. 2021). Flavones are synthesized at a branching point of the anthocyanidin/proanthocyanidin pathway, with flavanones serving as their direct biosynthetic precursors. Flavone formation in various tissues of a wide range of higher and lower plant species is primarily catalyzed by flavonoid glycosyltransferase. Flavones are present in all parts of plants, both above and below ground, including vegetative and reproductive organs such as stems, leaves, buds, bark, heartwood, thorns, roots, rhizomes, flowers, farina, fruits, seeds, as well as in root and leaf exudates or resin (Martens and Mithöfer 2005). In addition to their vital roles in the biochemistry, physiology, and ecology of plants, flavones are significant compounds for human nutrition and health. Growing evidence supports the health-protecting functions of flavonoid compounds, including antioxidative and antitumor effects in various cell lines, as well as anti-inflammatory, antibacterial, antiviral, and anti-atherosclerotic activities (Middleton et al. 2000). Flavones and their synthetic derivatives demonstrate various biological activities, including antioxidant, antitumor, antiallergic, anti-inflammatory, cardioprotective, and antimicrobial effects (Sing et al. 2014).

*Flavonols*, a subgroup of flavonoids, are polyphenolic natural compounds recognized for their strong antioxidant properties, largely attributed to their hydroxyl substitution patterns. These compounds are widespread throughout the plant kingdom and are abundant in fruits, vegetables, and other plant-derived foods (Samsonowicz and Regulska 2017). Flavonols are widely present in plant-based foods and contribute to a variety of colors, ranging from white to yellow (Rakha et al. 2022). To date, around 200 flavonols have been identified in plants and vegetables. They are predominantly found in the outer and aerial parts of plants, such as the skin and leaves, as their biosynthesis is stimulated by exposure to light. Structurally, flavonols share the characteristic flavonoid framework, consisting of two aromatic rings (A and B) connected by a three-carbon chain that forms an oxygen-containing heterocyclic ring (C ring). A defining feature of flavonols is the hydroxyl group at the 3-position. Compared to flavones, flavonols have a hydroxyl group at the 3-position of the C-ring, which can be glycosylated. The most commonly studied flavonols include quercetin, fisetin, kaempferol, myricetin, isorhamnetin, rhamnetin, azaleatin, and their respective glycosyl derivatives (Rakha et al. 2022). Additionally, various functional groups, primarily hydroxyl groups, can attach to the ring structures, enhancing their chemical reactivity. The presence of carbonyl (–C=O) and –OH groups allows flavonols to coordinate metal ions and form complexes (Samsonowicz and Regulska 2017). Among flavonols, quercetin is the most prevalent flavonol in plant-based foods. Rich sources of flavonols include kale, onions, strawberries, spinach, cauliflower, apples, grapes, tomatoes, and various berries. In addition to fruits and vegetables, herbal teas and red wine are also excellent sources of flavonols (Rakha et al. 2022). Flavonols exhibit numerous health-promoting effects, such as antioxidant, anti-cancer, anti-inflammatory, antibacterial, and antiviral properties. Evidence from animal models, human clinical trials, and cellular studies suggests that the mechanisms behind these benefits are complex and extend beyond simple free radical scavenging activity. This supports growing scientific evidence on the preventive effects of flavonols against various chronic diseases. Most research focuses on metabolic disorders and cardiovascular diseases (Barreca et al. 2021).

*Flavanones*, a subgroup of flavonoids, are polyphenolic natural compounds recognized for their strong antioxidant properties. Flavanones are a significant component of the human diet, primarily found in citrus fruits. Studies on flavanone consumption in human diets show substantial variability, with reported intakes ranging from a few milligrams to as much as 700 mg per day. Among the various flavonoids consumed, flavanols constitute the majority, followed by flavonols and flavanones, which are generally present in comparable amounts in typical diets (Zamora-Ros et al. 2016; Najmanova et al. 2020). The metabolic processes and absorption kinetics of flavanones are intricate and less comprehensively studied compared to the broader scope of flavonoid kinetics, which has been the subject of detailed reviews (Williamson et al. 2018; Najmanova et al. 2020). Flavanones are primarily present in citrus fruits from the Rutaceae family, including oranges, grapefruits, lemons, and limes, which is why they are often referred to as citroflavonoids or bioflavonoids. Additionally, they are found in smaller quantities in certain aromatic herbs such as oregano, peppermint, spearmint, and rosemary. The most abundant flavanone aglycones are naringenin, hesperetin, and eriodictyol (Khan et al. 2011; Najmanova et al. 2020).

*Anthocyanins* are water-soluble flavonoids predominantly found in flowers and fruits, responsible for producing a wide spectrum of red, purple, and blue hues. Anthocyanin is in the form of glycoside while anthocyanidin is known as the aglycone. While numerous anthocyanidins exist in nature, delphinidin, malvidin, cyanidin, peonidin, and pelargonidin are the most widely distributed and extensively studied (Rakha et al. 2022). Their stable coloring properties, combined with associated health benefits, make anthocyanins desirable for use in the food industry. The color of anthocyanins is influenced by two main factors: pH and the methylation of hydroxyl groups on the A and B rings (Khoo et al. 2017). Variability among anthocyanins arises from the number and position of –OCH_3_ and -OH groups on their basic structure and the carbon position to which carbohydrates are attached (Gülçin 2020). They are abundant in berries, where they contribute vibrant colors to flowers and fruits. For instance, blackberry, cranberry, and elderberry primarily contain a single type of anthocyanin, cyanidin. Additionally, compounds such as cyanidin-3-O-rutinoside, peonidin-3-O-rutinoside, and cyanidin-3-O-glucoside have been identified in both sour and sweet cherries. Peaches and plums are also rich sources of cyanidin-3-O-rutinoside and cyanidin-3-O-glucoside (Rakha et al. 2022). Anthocyanin aglycones exhibit greater solubility in alcohol compared to their glucoside counterparts, while glycosylated anthocyanins are highly soluble in water. The polyphenolic structure of anthocyanins imparts a hydrophobic characteristic, making them soluble in organic solvents like ethanol and methanol (Khoo et al. 2017).

*Chalcones* are naturally occurring aromatic ketones that serve as key intermediates in the biosynthesis of flavonoids and isoflavonoids. They belong to the group of polyphenolic compounds and are characterized by their chemical structure, 1,3-diphenyl-2-propen-1-one. This structure consists of two aromatic rings (A and B) connected by a three-carbon α, β-unsaturated carbonyl system. A putative chalkone has a ketone functional group (C=O), an alkene group (C=C) conjugated with the ketone and two phenyl (aromatic) rings attached to the α and β carbons. Chemically, it is named 1,3-diphenyl-2-propen-1-one. Chalcones and their derivatives are widely studied due to their structural simplicity and had a broad range of biological activities, biological activities (Yamali et al. 2020; Burmaoğlu et al. 2021; Tuğrak et al. 2021). Chalcones are naturally occurring in various plant species, where they serve as precursors in the biosynthesis of flavonoids and isoflavonoids. They contribute to the plant’s pigmentation, defense mechanisms, and growth regulation. They exhibit a wide range of biological activities, including antioxidant, anti-inflammatory, antimicrobial, antitumor, antidiabetic and enzyme inhibition properties (Akıncıoğlu et al. 2021; Aktas Anıl et al. 2022; Celik Onaret al. 2023). These compounds are also of significant interest in pharmacology due to their therapeutic potential and synthetic versatility. Chalcone is usually prepared by an aldol condensation between benzaldehyde and acetophenone (Burmaoğlu et al. 2022; Farzaliyev et al. 2025). Chalcones and their derivatives are secondary metabolites formed within the flavonoid biosynthesis pathway. Chalcones and their derivatives demonstrate a wide range of biological activities including anti-inflammation (Mahapatra et al. 2017; Burmaoğlu et al. 2020; Bilginer et al. 2020). Chemically, they are referred to as 1,3-diaryl-2-propen-1-one, characterized by two aromatic rings connected by a three-carbon chain. Most naturally occurring chalcones are polyhydroxylated aromatic compounds (Elkanzi et al. 2022; Koçyiğit et al. 2017, 2018; Burmaoğlu et al. 2019).

**Fig. 13:**
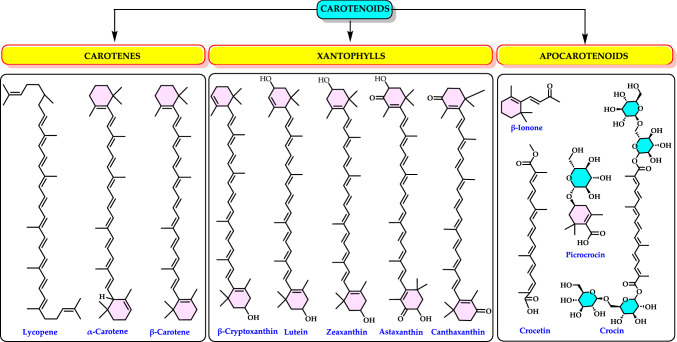
The classification and chemical structures of major carotenoids

*Carotenoids* are fat-soluble pigments naturally present in red, dark green, and yellow fruits and vegetables. They are highly effective natural antioxidants, known for their strong ability to quench singlet oxygen and for their role in scavenging other ROS (Bhardwaj et al. 2021). Carotenoids are tetraterpene antioxidants located in the plastids of both photosynthetic and non-photosynthetic plant tissues. Carotenoids in plant tissues are classified into two main types: those containing only hydrocarbons, such as carotene, and those containing one or more oxygen atoms, such as xanthophyll. If they contain oxygen due to oxidation or enzymatic addition, they are referred to as xanthophylls. Carotenoids are found not only in plants but also in algae and photosynthetic microorganisms. Carotenoids are a class of C40 liposoluble terpenoids, encompassing over 700 pigments produced by various plants and approximately 46 types of microorganisms. These compounds are stored in the plastids of fruits and vegetables, contributing to their vibrant red, orange, and yellow hues. It has been reported that human blood contains approximately 40 of these carotenoids (Tufail et al. 2024). Carotenoids are natural colorants and lipid-soluble plant pigments that are widely found in nature and have significant antioxidant activity. They are a group of natural pigments with a basic structure consisting of at least 40 carbon atoms and an extensive conjugated double bond system. Carotenoids are synthesized by plants and microorganisms, but not by animals. In plants, they can be either esterified with fatty acids or unesterified (Ribeiro et al. 2018). Carotenoids are further classified according to their structure into xanthophylls and carotenes. Carotenes contain only carbon and hydrogens and lack oxygen (Tufail et al. 2024). In the human diet, carotenoids and their derivatives, such as retinol, have a wide range of biological effects, including the modulation of gap junction communication, regulation of growth factors, provitamin A effects, stimulation of the immune response, cell differentiation, regulation of the cell cycle, and playing an important role as antioxidants. Due to their antioxidant properties, a diet rich in carotenoids is associated with a reduced risk of developing various disorders caused by oxidative stress, such as certain types of cancer, ophthalmologic, and cardiovascular diseases (Monego et al. 2017). It has been reported that there are over 700 carotenoids, with 40 of them being consumed in the human diet, mainly sourced from vegetables and fruits. Their chemical properties are largely determined by the presence of an extended conjugated double bond system, which is substituted with various end groups. Carotenoids are among the major food micronutrients in the human diet. In plants, they exhibit potential antioxidant properties due to their chemical structure (Gulcin 2012). In the human body, carotenoids also play a role in the antioxidant defense system. They can be divided into two broad categories: carotenoid hydrocarbons, also known as carotenes, which contain specific end groups, such as lycopene and β-carotene; and oxygenated carotenoids, or xanthophylls, such as zeaxanthin and lutein (Carocho and Ferreira 2013). Based on their chemical structure, carotenoids can be classified into carotenes and xanthophylls. Examples of carotenes include α-carotene, β-carotene, and lycopene, which are non-oxygenated carotenoids. Lutein, zeaxanthin, astaxanthin, and canthaxanthin are examples of xanthophylls, the oxygenated derivatives of carotenes (Ribeiro et al. 2018). The most significant carotenoids in the human diet include β-carotene, lycopene, lutein, β-cryptoxanthin, zeaxanthin, and astaxanthin (Riccioni 2009). Numerous observational studies have supported the idea that antioxidants like carotenoids and vitamin E, or their metabolites, are associated with a lower risk of cardiovascular diseases (Lichtenstein 2009). Carotenoids may serve as an affordable means of prevention and potentially as a treatment, although human intervention trials have yielded mixed results, with some positive findings, many null findings, and some indications of harm in high-risk populations. Carotenoids are the main pigments responsible for the color of vegetables and fruits, including β-carotene, lutein, zeaxanthin, and lycopene, which contribute to the red color of tomatoes and other fruits. The molecular structure of carotenoids is highly sensitive and particularly vulnerable to photodegradation and degradation under exposure to light (especially UV), high temperatures, and oxygen. Consequently, carotenoids degrade easily when exposed to light, heat, or oxidizing agents. Proper handling is crucial to avoid structural changes that may render them inactive or unstable (Rodriguez-Bonilla et al. 2017). The color of these pigments is attributed to their many conjugated carbon double bonds. Each double bond reduces the energy required for electrons to transition to higher energy states, allowing the molecule to absorb light at progressively longer wavelengths (Riccioni 2009). Because of their antioxidant potential, carotenoids are widely used in the food industry for their strong coloration. Recent smaller interventional studies with carefully selected populations, such as those under high levels of oxidative stress, have shown mostly positive results (Lichtenstein 2009). The main antioxidant effect of carotenoids is due to their ability to quench singlet oxygen (^1^O_2_). When carotenoids absorb energy, they dissipate it through rotational and vibrational interactions with the solvent, returning to their unexcited state and enabling them to neutralize more radical species. This process occurs when carotenoids have conjugated double bonds. The only free radicals that can completely destroy these pigments are peroxyl radicals (ROO·). Carotenoids are relatively unreactive but can decay and form non-radical compounds, which may help terminate free radical attacks by binding to these radicals (Carocho and Ferreira 2013). Carotenoids are natural fat-soluble compounds with chemical properties closely linked to their extended conjugated double bond systems. In the human body, carotenoids like β-carotene, lycopene, lutein, β-cryptoxanthin, zeaxanthin, and astaxanthin play a significant role in mitigating cardiovascular diseases (Lichtenstein 2009). Carotenoids are classified into two groups based on their chemical structure: (1) carotenes, such as lycopene, β-carotene, and α-carotene, which are composed exclusively of carbon and hydrogen, and (2) xanthophylls, including zeaxanthin, meso-zeaxanthin, and lutein, which function as macular pigment carotenoids (Rodriguez-Bonilla et al. 2017). The classification and chemical structures of carotenoids are illustrated in Fig. 13.

*Carotene* is a natural pigment belonging to the class of carotenoids, specifically the hydrocarbon carotenoids composed solely of carbon and hydrogen. Carotenes are tetraterpenes, composed of 40 carbon atoms arranged in a conjugated polyene chain. This structure allows them to absorb light and exhibit their characteristic colors. These compounds are synthesized by plants, algae, and certain bacteria, and they are responsible for the orange, red, and yellow coloration in many fruits and vegetables (Mascio et al. 1989). Carotenes play essential roles in plants as antioxidants and in the process of photosynthesis by protecting against photooxidative damage. In humans, some carotenes serve as precursors to vitamin A, a vital nutrient. Carotene can be converted into retinol (vitamin A) in the human body, which is crucial for vision, immune function, and skin health (Britton et al. 2004). They neutralize free radicals, protecting cells from oxidative damage (Di Mascio et al. 1989). Carotenes protect chlorophyll from damage by excessive light and act as accessory pigments in photosynthesis in plants. They were abundantly found in orange and yellow vegetables including carrots, pumpkins, sweet potatoes, green leafy vegetables including spinach, kale and fruits including mangoes, papayas, and apricots (Stahl and Sies 2005). The most abundant types of carotene are lycopene, α-carotene, and β-carotene.

*Lycopene* is a lipid-soluble tetraterpenoid composed of eight isoprene units. Discovered by Ernest in 1959, it was initially named “lycopin” by Schnuck, while Escher later proposed its chemical structure. Its name originates from the botanical classification of tomatoes, *Solanum lycopersicum*. Today, lycopene is a common component of human diets worldwide. Since humans cannot synthesize lycopene, it must be obtained through dietary sources. It belongs to the carotene subgroup of carotenoids. It is an acyclic polyunsaturated hydrocarbon with no vitamin A activity. Classified as a natural pigment, lycopene is responsible for the distinct red coloration in various fruits and vegetables, particularly tomatoes, watermelon, and pink grapefruit. Among its dietary sources, tomatoes and tomato-based products are the most abundant, reportedly supplying 80% of the total lycopene intake in the human diet (Tufail et al. 2024). Also, this carotenoid pigment was found other red fruits such as watermelon, papaya, pink grapefruit, and pink guava. Lycopene absorbs most of the visible spectrum, giving it its characteristic red color. In the small intestine, lycopene is integrated into lipid micelles and absorbed by enterocytes, either through passive diffusion or with the assistance of scavenger receptor class B type 1. These receptors also aid in the absorption of other carotenoids, such as lutein and β-carotene. A fraction of the lycopene undergoes cleavage by β-carotene oxygenase in the intestine. Nevertheless, the majority remains unchanged, becomes incorporated into chylomicrons, and is transported through the lymphatic system (Kulawik et al. 2024).

*α-Carotene* is a naturally occurring carotenoid and a type of provitamin A found in many fruits and vegetables. Structurally, it is a C_40_ tetraterpene hydrocarbon and an isomer of β-carotene, with a single β-ionone ring at one end and an ε-ionone ring at the other. This asymmetry distinguishes it from β-carotene, which has two β-ionone rings. It is known that carotenoids are categorized as pro-vitamin A comprised of the unsubstituted β-ionone ring (γ-carotene, α-carotene, β-carotene, and β-cryptoxanthin) which can be converted into retinal. α-Carotene is predominantly found in carrots, pumpkins, sweet potatoes, leafy greens such as spinach, fruits like mandarins and oranges (Krinsky and Johnson 2005). As a provitamin A, α-carotene can be converted into retinol in the body, although it is less efficient in this process compared to β-carotene. It has strong antioxidant properties, helping to neutralize free radicals and protect cells from oxidative stress, which is associated with aging and chronic diseases (Stahl and Sies 2005). It was reported that studies have suggested high dietary intake of α-carotene is linked to a reduced risk of certain chronic diseases, including cardiovascular diseases and some cancer (Cheng et al. 2009). Cooking can increase the bioavailability of α-carotene by breaking down cell walls in plant tissues (Van het Hof et al. 2000a, b). It was found that higher blood concentrations of α-carotene were associated with lower mortality from all causes, including cardiovascular disease (Li et al. 2011a, b).

*β-Carotene* is a vividly red–orange pigment widely found in plants and fruits. It is an organic compound classified chemically as a hydrocarbon and, more specifically, as an isoprenoid due to its derivation from isoprene units. β-Carotene is biosynthesized from geranylgeranyl pyrophosphate and belongs to the carotene group, which are tetraterpenes synthesized biochemically from eight isoprene units, resulting in a 40-carbon structure. Within this group of carotenes, β-carotene is distinguished by the presence of beta-rings at both ends of its molecule (Susan and Van Arnum 1998). Carotenes are structurally defined by a C_40_H_56_ conjugated polyene backbone, which enables the electrons in their double bonds to easily become delocalized.

*Xanthophylls* are oxygen-containing carotenoids that are responsible for the yellow, orange, and red pigmentation observed in many plants, algae, and certain animal tissues. They belong to the carotenoid family and differ from carotenes by the presence of oxygen atoms, typically in the form of hydroxyl (-OH) or keto (=O) groups (Krinsky and Johnson 2005). They are derived from carotenes by oxidation. Their structure is characterized by a C40 tetraterpene backbone, with oxygen-containing functional groups that make them more polar compared to carotenes. The most abundant of xanthophylls are lutein, which is essential for eye health and is a major component of the macula in the human retina, zeaxanthin as a structural lutein isomer, plays a role in protecting the eyes from blue light damage and astaxanthin, which exhibits strong antioxidant activity and found in marine organisms like shrimp and salmon (Park et al. 2010). The plentiful xanthophylls are cryptoxanthin, lutein, zeaxanthin, astaxanthin and canthaxanthin.

*β-Cryptoxanthin* is an oxygenated carotenoid with a chemical structure similar to β-carotene, but it is more polar. β-cryptoxanthin, a main kind of xanthophyll, is abundant in Satsuma mandarin orange (*Citrus unshiu*). While β-carotene is abundantly present in various fruits and vegetables, β-cryptoxanthin is concentrated in only a limited number of foods including butternut and hubbard squashes, persimmons and hot chili peppers (Maiani et al. 2009). The content of β-cryptoxanthin in fruits and vegetables, like other carotenoids, depends on factors such as cultivar, maturity stage, growing conditions, storage methods, and seasonality. Many of the richest sources of β-cryptoxanthin are citrus fruits, and its concentrations in these fruits and in human plasma peak during the ripening season, typically in late autumn and winter (Burri et al. 2024). Foods rich in β-cryptoxanthin, such as tangerines and peaches, are often consumed raw or as juices, while others like pumpkin and butternut squash are cooked or used in mixed dishes. Processing reduces the levels of β-cryptoxanthin in foods. However, cooking and food processing can either enhance or reduce the bioaccessibility of β-cryptoxanthin (i.e., the amount available for absorption by the body). Also, β-cryptoxanthin is a common carotenoid that is found in human blood and tissues. It has several important functions for human health including antioxidant defense and cell-to-cell communication roles. It has been found to have some potential-anabolic effects on bone due to stimulating osteoblastic bone formation and inhibiting osteoclastic bone resorption (Yamaguchi and Uchiyama 2003; 2004). Mild, shorter cooking methods typically improve bioaccessibility by softening cell walls and denaturing proteins that bind to β-cryptoxanthin. Conversely, harsher or prolonged processes, such as refining, drying, or extended boiling, can isomerize or destroy carotenoids. Despite its presence in a limited range of non-staple foods, β-cryptoxanthin is a common carotenoid in human blood. In the United States, it is generally the fourth most abundant carotenoid (Olmedilla et al. 2001).

*Lutein* is the most abundant xanthophyll in higher plants. Lutein, chemically known as 3,3′-dihydroxy-α-carotene, is a carotenoid compound that is recognized for its yellow or orange pigmentation. This pigment is widely used as a natural food coloring and as an ingredient in animal feed (Chandra et al. 2019). Lutein’s remarkable antioxidant properties play a role in supporting the immune system, inhibiting cancer, reducing age-related cardiovascular sclerosis, and preventing coronary heart disease (Starska-Kowarska 2022). Additionally, it serves as a “blue light filter,” which helps decrease the risk of cataracts and age-related macular degeneration (Peng et al. 2016), making it increasingly popular among consumers. Despite its functional advantages, lutein’s complex structure requires natural extraction from plants, as it cannot be synthetically produced. Previous studies in China have mainly focused on extracting lutein from marigolds (*Tagetes erecta*), but due to the high cost and limited availability of marigold resources, alternative sources are being sought (Zhang et al. 2025).

*Zeaxanthin* is one of the two main xanthophyll carotenoids found in the retina of the eye. Of the more than 700 carotenoids found in nature, only 40–50 are present in the human diet. Among these, only about 14 carotenoids are found in human circulation. Lutein and zeaxanthin are unique among the carotenoids present in circulation, as they are the only carotenoid pair found in significant amounts in the macula of the human eye. In the macula, lutein and zeaxanthin act as filters for harmful blue light wavelengths and function as antioxidants to prevent free radical damage. These carotenoids are also found in significant amounts in human skin (Hata et al. 2000). In the central macula, zeaxanthin is the dominant component, while lutein is more prevalent in the peripheral retina. The name zeaxanthin originates from *Zea mays*, where it serves as the primary yellow pigment (Krishnadev et al. 2010). Zeaxanthin, present in the human diet and various dietary supplements, particularly those aimed at supporting eye health, may also enhance the skin's antioxidant capacity (Roberts 2013). In cells, lutein and zeaxanthin are predominantly located within membranes. Due to their molecular dimensions and the presence of -OH groups at each end of their structures, these carotenoids can interact with membrane structures in a way that allows them to span from the inner to the outer membrane surface. Furthermore, the positioning of the double bonds within lutein's structure enables it to align parallel to the membrane surface (Sujeck et al. 1999; Roberts 2013).

*Astaxanthin* is a vibrant, fat-soluble pigment found in microalgae, yeast, salmon, trout, krill, shrimp, crayfish, crustaceans, and the feathers of certain birds. It is responsible for the red coloration of salmon flesh and cooked shellfish. Unlike some carotenoids, astaxanthin is not converted into retinol (vitamin A) in the human body. While excessive vitamin A intake can be toxic to humans, astaxanthin has a much lower toxicity level. It functions as an antioxidant, though its antioxidant activity is slightly lower compared to other carotenoids (Mortensen and Skibsted 1997; Gulcin 2012). It exhibits antioxidant, antibacterial, and anti-apoptotic properties, and it is also effective in scavenging free radicals (Ekpe et al. 2018). Unlike other carotenoids, which are less abundant in animals or humans for extraction, astaxanthin can be sourced from animal products. Unlike lycopene and β-carotene, which are composed only of carbon and hydrogen, astaxanthin contains oxygenated functional groups (Mori et al. 2013). Its chemical formula is C_40_H_52_O_4_, and its molecular structure includes a conjugated double-bond chain, an unsaturated ketone, and a hydroxyl group at the end of the chain. These functional groups can either attract or donate unpaired electrons to free radicals, helping to scavenge them and thus exhibiting antioxidant properties (Nishida et al. 2023). Like other carotenoids, astaxanthin is particularly sensitive to light, heat, and oxygen. It is insoluble in water but soluble in fats, chloroform, acetone, benzene, and most other organic solvents. It was reported that astaxanthin was first isolated in 1949 in small quantities from the shells of marine crustaceans, marking its initial discovery (Nair et al. 2023). Astaxanthin is predominantly found in *Haematococcus pluvialis*, *Phaffia rhodozyma* and *Cladophora aegagropila* (Ambati et al. 2014). It has a wide range of applications, including in aquaculture (Stachowiak and Szulc 2021) and the production of antimicrobial drugs (Weintraub et al. 2017). However, due to its limited availability and high cost, extracting and purifying natural astaxanthin can be difficult, which restricts its use (Jiang et al. 2024).

*Canthaxanthin* is a naturally occurring carotenoid pigment belonging to the xanthophyll class of carotenoids, known for its reddish-orange color. It is found in various natural sources, including fungi, algae, certain fish species like salmon and trout, crustaceans, and some birds. Its role as a pigment contributes to the coloration of animal tissues and eggs in some species. It is a tetraterpenoid composed of 40 carbon atoms with conjugated double bonds, which are responsible for its vibrant color and antioxidant properties. Canthaxanthin dissolves in fats and oils but not in water. It enhances animals' resistance to hypoxic stress and supports reproductive development by boosting the secretion of reproductive hormones in hens. Additionally, it protects ram sperm from oxidative stress (Zhao et al. 2023; Jiang et al. 2024). It is a carotenoid with a structure similar to β-carotene but with a hydroxyl group (–OH) attached at one of the end rings. It has a characteristic conjugated polyene structure, which gives it its antioxidant properties and color. It is lipid-soluble and can be absorbed in the body along with dietary fats. This naturally occurring carotenoid pigment belongs to the xanthophylls family, which are oxygenated derivatives of carotenes. It is a precursor (provitamin) to vitamin A, meaning the body can convert it into retinol, the active form of vitamin A, which plays a crucial role in vision, immune function, and skin health (Krinsky and Johnson 2005). β-Cryptoxanthin is found in various fruits like oranges, tangerines, and papayas, vegetables like red peppers, pumpkins, and some leafy greens, and plant-based foods (Burri et al. 2024). Canthaxanthin can enhance animals' resistance to hypoxic stress and support their reproductive development by stimulating the secretion of reproductive hormones in hens (Chung and Sontag 2020).

### Antioxidant vitamins

*Vitamin A* (*Retinol*) is a fat-soluble vitamin and an essential nutrient made up of unsaturated organic compounds, such as retinal, retinol, retinoic acids, and several provitamin A carotenoids, which must be sufficiently obtained through the diet. It plays a crucial role in numerous life processes, including reproduction, immune system functionality, and cellular differentiation (Bhardwaj et al. 2021). It is a carotenoid derivative, which produced in the liver and results from the breakdown of β-carotene. It plays a vital role in cell growth, metabolism, immunity, vision, and reproduction (Wiseman et al. 2017). Retinol deficiency is a widespread global health issue, particularly prevalent among young children in low-income countries, leading to high rates of mortality and disability. Poor absorption of retinol results in its deficiency, disrupting essential physiological processes. Retinol naturally occurs in dark leafy green vegetables, milk, liver, fish, and other dairy products (Hombali et al. 2019). It is absorbed in the duodenum after being hydrolyzed by pancreatic enzymes and emulsified with fats and bile. While the majority of vitamin A is stored in liver cells, a significant amount is also retained in adipose tissue and the pancreas (Saeed et al. 2017; Patil et al. 2023) It has beneficial effects on the skin, eyes, and internal organs. Its antioxidant activity stems from its ability to react with ROO· radicals, preventing them from propagating lipid peroxidation (Jee et al. 2006; Carocho and Ferreira 2013). Additionally, it plays a protective role in plants against photooxidative damage. These compounds are highly effective antioxidants, particularly in scavenging singlet oxygen (Di Mascio et al. 1989) and ROO· radicals (Stahl and Sies 2003).

**Fig. 14:**
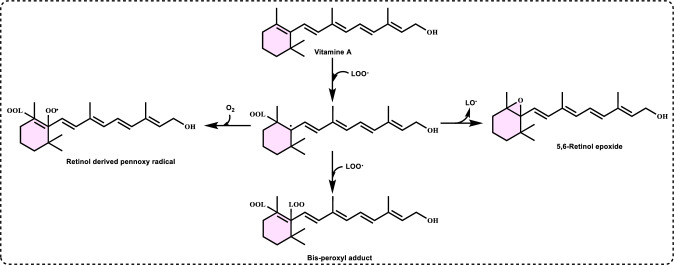
The purposed mechanism of radical scavenging ability of vitamin A

The antioxidant properties of vitamin A were first identified by Monaghan and Schmitt (1932), who demonstrated its ability to protect lipids from rancidity. Several reviews have since been published, summarizing the fundamental structural and metabolic characteristics of vitamin A, as well as its potential role as an antioxidant in relation to heart diseases. Vitamin A plays a crucial role as an antioxidant in safeguarding human LDL from copper-induced oxidation (Fig. 14) (Nimse and Pal 2015).

**Fig. 15:**
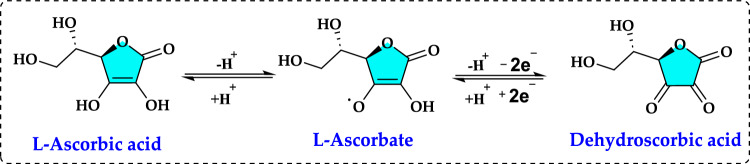
Mechanistic steps on antioxidant action of ascorbic acid

*Ascorbic acid* (*Vitamin C*) exists in two forms with antioxidant activity: l-ascorbic acid and l-dehydroascorbic acid. Both forms are absorbed through the gastrointestinal tract and can be enzymatically converted into each other in vivo. Studies have shown that ascorbic acid effectively scavenges O_2_·^−^, H_2_O_2_, OH, ^1^O_2_, and reactive NO_2_·radicals (Barros et al. 2011). It is regarded as one of the most potent and least toxic natural antioxidants (Weber et al. 1996). The beneficial biological effects of ascorbic acid are widely acknowledged. However, ascorbic acid primarily serves three natural functions of particular relevance to reproduction, all of which rely on its reducing properties: it is essential for collagen synthesis, the production of peptide and steroid hormones, and the prevention of oxidation in biological molecules. Interestingly, the concentration of ascorbic acid in human follicular fluid is significantly higher than in blood serum, indicating active transport against a concentration gradient (Bhardwaj et al. 2021). As a water-soluble vitamin, it is found in high concentrations in many foods and plants. Ascorbic acid typically reacts with oxidants, terminating chain radical reactions via electron transfer. Its unique ability to transfer a single electron makes it particularly effective. Ascorbic acid is a powerful reductone, functioning as a vinylogous carboxylic acid, where the electrons from the double bond, hydroxyl group lone pair, and carbonyl double bond form a conjugated system. The two primary resonance structures stabilize the deprotonated conjugate base of ascorbic acid, rendering the hydroxyl group significantly more acidic than typical hydroxyl groups. In other words, ascorbic acid can be classified as an enol, where the deprotonated form is a stabilized enolate (Fig. 15).

**Fig. 16:**
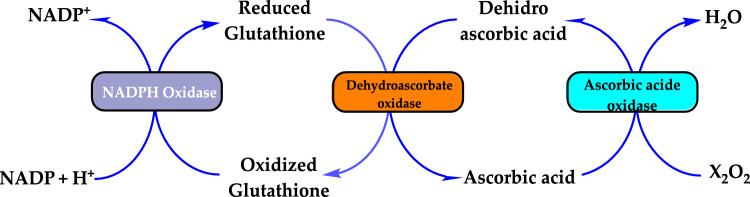
The enzymatic conversion of dehydroascorbate to ascorbic acid by dehydroascorbate oxidase

Ascorbic acid has been observed to regenerate α-tocopherol from its tocopheroxyl radical both in vivo and in vitro, thereby restoring its antioxidant activity (Kamal-Eldin and Budilarto 2015). Additionally, human plasma contains approximately 60 µmol of ascorbate. Upon interaction with ROS, ascorbic acid is oxidized to dehydroascorbate through the intermediate ascorbyl free radical. As shown in Fig. 16, dehydroascorbate is converted back to ascorbic acid by the enzyme dehydroascorbate reductase. Consequently, dehydroascorbate is present in significantly lower levels compared to ascorbate.

Ascorbic acid as a ROS scavenger, has been demonstrated to effectively neutralize O_2_^–^, H_2_O_2_, OH, and ^1^O_2_. In aqueous environments, ascorbic acid also efficiently scavenges reactive nitrogen oxide species. The primary dietary sources of ascorbic acid include fruits particularly citrus fruits, kiwi, cherries, melons and vegetables such as tomatoes, leafy greens, broccoli, cauliflower, *Brussels sprouts*, and cabbage, where its content can exceed 1 g of ascorbate per kg of fresh weight. Also, Bursal and Gülçin (2011) reported approximately 1 g of vitamin C per kg of lyophilized kiwi fruit (*Actinidia deliciosa*) extract. At lower doses (100 mg), the bioavailability of vitamin C from synthetic and natural sources is nearly identical (Mangels et al. 1993). However, absorption efficiency decreases as the dose increases. Studies conducted in vitro indicate that vitamin C can regenerate tocopherol from the tocopheroxyl radical, which is formed during the inhibition of lipid peroxidation by vitamin E (Niki et al. 1985). Ascorbic acid is recognized as a highly effective antioxidant, participating in numerous physiological and biochemical processes. However, under certain conditions, it can act as a prooxidant. This occurs when it interacts with iron (Fe) and copper (Cu), reducing Fe^3+^ to Fe^2+^ or Cu^3+^ to Cu^2+^, which subsequently reduce H_2_O_2_ to OH· (Duarte and Lunec 2005). Additionally, it has been reported that some phenolic antioxidants lose their activity at higher concentrations and can behave as prooxidants by initiating oxidation reactions (Gulcin 2020).

$${\text{ROO}}^{ \bullet } + {\text{ Toc}} - {\text{OH}} \to {\text{ROOH}} + {\text{Toc}} - {\text{O}}^{ \bullet }$$

$${\text{Toc}} - {\text{O}}^{ \bullet } + {\text{ Ascorbic acid}} \to {\text{Toc}} - {\text{OH}} + {\text{Ascorbate}}$$

This process enables the transfer of a radical load from a lipophilic compartment to an aqueous compartment, where it is managed by efficient enzymatic defense mechanisms. However, it should be noted that ascorbate may also act as a prooxidant in vivo. In the presence of free transition metal ions, ascorbate can generate OH·, leading to the initiation of lipid peroxidation. Nonetheless, the levels of free transition metals in vivo are minimal, as they are effectively bound to proteins. Additionally, ascorbic acid has other well-established biological roles, including serving as a cofactor for several essential enzymes, such as hydroxylases (Levine et al. 1996).

*Vitamin E* (*Tocopherols*), which a highly potent lipid-soluble, chain-breaking antioxidant, plays a crucial role in neutralizing lipid peroxyl radicals (LOO·) during the lipid peroxidation process, effectively halting the cascade of damaging reactions within cellular membranes (Nimse and Pal 2015). Tocopherols are fat-soluble vitamins with antioxidant properties that protect living organisms, particularly body cells, from damage caused by free radicals and ROS. Vitamin E is a collective term for tocopherols, which belong to a class of chemical phenolic compounds (ArasHisar et al. 2004; Gülçin et al. 2005a; b; c). These are among the most widely recognized and used antioxidants (Pokorny 1987; Koksal et al. 2011; Cetinkaya et al. 2012). Tocopherols naturally occur in a variety of foods, including green leafy vegetables and fatty foods like vegetable oils, seeds, nuts, and egg yolks. Tocopherols consist of eight isoforms, divided into four tocopherols and four tocotrienols, collectively referred to as tocols. These monophenolic compounds include eight chromanol homologues with vitamin E activity in the diet. Tocopherols and tocotrienols are further classified into four isomers within each group: α-, β-, γ-, and δ-tocopherols, resulting in a total of eight tocopherol isomers (Table 5). Among these, α-tocopherol is the most abundant and biologically potent isoform.

**Table 5:**
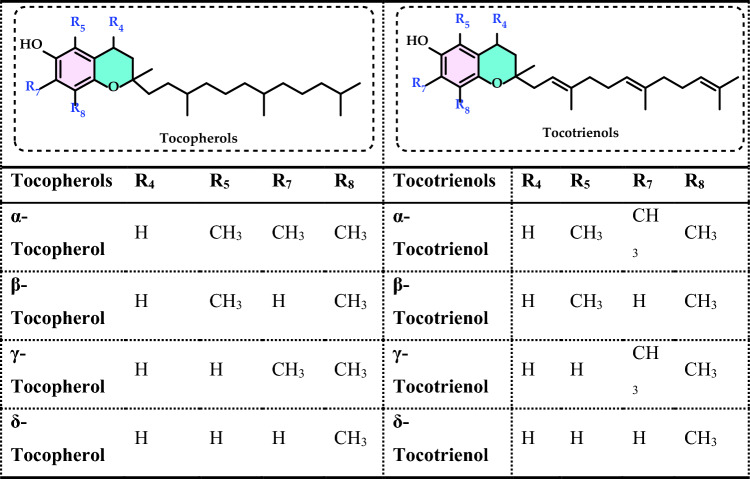
The classification of tocopherols and tocotrienols

Tocopherols are characterized by a 6-chromanol group and an apolar phytal chain. The prefix α-, β-, γ-, or δ- indicates the number and position of methyl groups (–CH_3_) attached to the chroman ring (Shahidi and Ambigaipalan 2015). While the chroman head group provides antioxidant activity, the phytyl tail has no significant impact. These molecules feature a chromanol ring with a hydroxyl (–OH) group that donates a hydrogen atom to neutralize free radicals, as well as a hydrophobic side chain that facilitates penetration into biological membranes (Burton and Ingold 1981).

Vitamins E and C work synergistically, with ascorbic acid regenerates Vitamin E by reducing the tocopheroxyl radical to an intermediate form, thereby restoring its antioxidant potential (Carocho and Ferreira 2013). α-Tocopherol with its three methyl substituents is known as one of the most biologically reactive antioxidants among the four well-known lipoperophilic isomers of tocopherol. Regardless of the existing tocopherol groups, they are responsible for scavenging lipid peroxy radicals, oxygen free radicals, and singlet oxygen (Zandi and Schnug 2022). α-Tocopherol plays a crucial role in halting lipid peroxidation by donating a phenolic hydrogen to lipid peroxyl radicals (ROO·), forming unreactive tocopheroxyl radicals that cannot propagate oxidative chain reactions. It is the primary lipid-soluble, chain-breaking antioxidant found in plasma, red blood cells, and tissues, protecting lipid structures, particularly membranes (Carocho and Ferreira 2013). Being a hydrophobic antioxidant, α-tocopherol is soluble only in organic solvents and membranes, which poses challenges for experimental studies in aqueous systems. This limitation has led to the use of α-tocopherol analogues, which are more suitable for homogeneous, aqueous environments while maintaining significant antioxidant activity. Among these, Trolox as a derivative of α-tocopherol where the polyisoprenoid tail is replaced by a carboxyl group, stands out due to its moderate water solubility. In Trolox, the carboxyl group confers water solubility, while the chromanol moiety ensures its antioxidant properties (Aksu et al. 2015; Celik et al. 2018).

Tocopherols are found, at least in trace amounts, in nearly all food sources. Generally, foods with the highest concentrations of vitamin E include vegetable oils, followed by nuts, seeds, and whole grains. The most significant antioxidant in this group is α-tocopherol, which exhibits lower antioxidant activity in edible oils compared to other tocopherols. α-Tocopherol is a lipid-soluble antioxidant that not only reduces lipid peroxidation but may also have intracellular effects. It resides in the outer membranes of cells and cell organelles, disrupting oxidation chains and protecting membranes from further damage (Godbout et al. 2004). Among the vitamin E isoforms, α- and γ-tocopherols are the most abundant in diets and tissues. However, α-tocopherol is the most biologically active and clinically significant. Similar to α-tocopherol, the γ-tocopherol isoform reacts with ROS and RNS, offering potential benefits against inflammation. While α-tocopherol is the predominant form found in supplements and European diets, γ-tocopherol is more common in the American diet (Jiang et al. 2001).

**Fig. 17:**
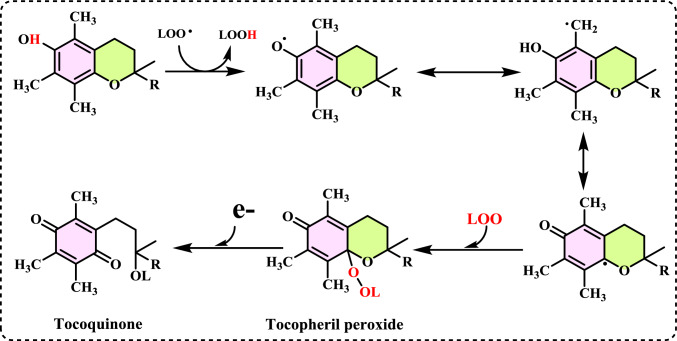
Radical scavenging mechanism of α-tocopherol

The compound α-tocopherol is a frequently added form of tocopherol in food products. Tocopherols act as antioxidants by donating a hydrogen atom from their hydroxyl group to lipid peroxyl radicals (ROO·). The formation of a tocopherol radical is stabilized by delocalizing the unpaired electron across the aromatic ring structure (Fig. 17). These compounds are highly lipophilic, functioning in cell membranes or lipoproteins. Their primary antioxidant role is to inhibit lipid peroxidation by scavenging lipid peroxyl radicals, resulting in the production of lipid hydroperoxides and tocopheroxyl radicals (Diplock et al. 1998).

This radical forms of non-radical products, including stable peroxides, which can be further reduced to tocoquinones or tocopherol dimers. α-Tocopherol has also been linked to slowing the decomposition of hydroperoxides (Frankel 1996). According to Etminan et al. (2005), α-tocopherol has a protective effect against Parkinson’s disease. A deficiency of tocopherols can lead to neurological issues, such as spinocerebellar ataxia and myopathies (Brigelius-Flohé and Traber 1999). The hydrogen-donating ability of tocopherols in fats, oils, and lipoproteins follows the order δ-tocopherol > β-tocopherol ≈ γ-tocopherol > α-tocopherol. It has been observed that the antioxidant potency of α-tocopherol in butter oil triacylglycerols is lower than that of γ-tocopherol (Lampi and Piironen 1998).

*Trolox* (6-hydroxy-2,5,7,8-tetramethylchroman-2-carboxylic acid) is a water-soluble analog of α-tocopherol. It is a synthetic compound derived by modifying the structure of α-tocopherol, replacing its hydrophobic polyisoprenoid tail with a hydrophilic carboxyl group. This structural modification makes Trolox soluble in aqueous solutions, unlike α-tocopherol, which is only soluble in fats and organic solvents. Trolox does not occur naturally and is synthesized in laboratory conditions for research and experimental purposes. It is widely used in the fields of biochemistry and food science to evaluate antioxidant properties of natural and synthetic compounds. Unlike α-tocopherol, Trolox is soluble in water, which makes it suitable for studying antioxidant activity in aqueous environments. Unlike tocopherols, it is not a naturally occurring antioxidant (Bursal et al. 2019; Gulcin et al. 2019a). Its antioxidant activity is derived from the chromanol moiety, which donates hydrogen atoms to neutralize free radicals, breaking oxidative chain reactions. Trolox serves as a standard to optimize the antioxidant activity found in biological fluids, cells, tissues, and natural extracts. It exhibits similar antioxidant properties and widely used as a model compound for α-tocopherol in biological, biochemical, and pharmaceutical studies to neutralize free radicals and mitigate oxidative stress damage (Garibov et al. 2016; Çelik et al. 2018). Trolox was used as a standard in assays measuring antioxidant capacity, such as the Trolox Equivalent Antioxidant Capacity (TEAC) assay. TEAC method evaluates the antioxidant capacity of a given sample by comparing it to a standard solution of Trolox under the same conditions (Shivakumar and Kumar 2018; Aras et al. 2019; Gulcin et al. 2019b; 2020).

**Fig. 18:**
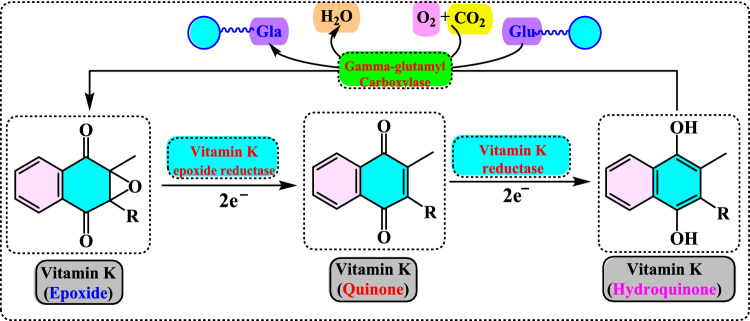
The regeneration of vitamin K cycle

*Vitamin K* is a group of fat-soluble compounds crucial for the posttranslational modification of protein-bound glutamate residues into carboxyglutamates in several target proteins. The antioxidant protection provided by these vitamins is due to their 1,4-naphthoquinone structure (Fig. 18). The two natural forms of this vitamin are K1 and K2 (Vervoort et al. 1997; Carocho and Ferreira 2013). The human body needs vitamin K for the post-synthesis modification of specific proteins essential for blood coagulation and for regulating calcium binding in bones and other tissues. This process is completed through the modification of “Gla proteins” by the enzyme gamma-glutamyl carboxylase, which relies on vitamin K as a cofactor. Without vitamin K, blood clotting is severely compromised, leading to uncontrolled bleeding. Studies indicate that a deficiency in vitamin K may also weaken bones, potentially increasing the risk of osteoporosis, and could encourage the calcification of arteries and other soft tissues (Marriott et al. 2020).

Vitamin K encompasses structurally similar, fat-soluble vitamers that are present in foods and available as dietary supplements. The term “Vitamin K” refers to several chemical compounds that share a common quinone ring structure but differ in the length and saturation level of their carbon tail and the number of repeating isoprene units in their side chain (Chemically, the vitamin K family consists of derivatives of 2-methyl-1,4-naphthoquinone. Vitamin K includes two natural forms including vitamin K_1_ (phylloquinone) and vitamin K_2_ (menaquinone). Vitamin K_2_, in turn, comprises various related chemical subtypes that differ in the lengths of their carbon side chains, composed of isoprenoid units. Among these, the most extensively studied are menaquinone-4 and menaquinone-7 (Marriott et al. 2020). Vitamin K hydroquinone (reduced form) is oxidized to vitamin K epoxide (oxidized form). The recycling of vitamin K epoxide (oxidized form) to hydroquinone (reduced form) is carried out by two reactions that reduce vitamin K epoxide to vitamin K quinone and then to vitamin K hydroquinone. proteins result in the oxygenation of vitamin K hydroquinone to vitamin K epoxide. The vitamin K epoxide product is then recycled to vitamin K hydroquinone by two reactions. The vitamin K oxidoreductase is known to perform the first reaction, i.e. vitamin K epoxide to vitamin K quinone, and this study tests whether it can perform both reactions to fully reduce vitamin K epoxide (Rishavy et al. 2013). Vitamin K1 or phylloquinone is synthesized by plants and is the predominant form in the diet. Vitamin K_2_ includes a range of vitamin K forms collectively referred to as menaquinones. Most menaquinones are synthesized by human intestinal microbiota and found in fermented foods and in animal products. Menaquinones differ in length from 1 to 13 repeats of 5-carbon units in the side chain of the molecules. These forms of vitamin K are designated menaquinone-n, where n stands for the number of 5-carbon units. Widely used in animal husbandry, the synthetic compound known as menadione (vitamin K_3_) is a provitamin that needs to be converted to menaquinone-4 to be active (Table 6) (Rishavy et al. 2013).

**Table 6:**
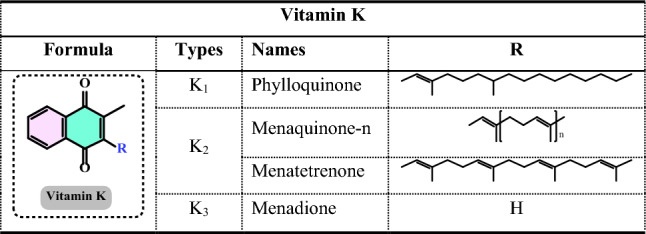
Chemical structures of vitamin K

Vitamin K oxidoreductase plays a key role in recycling vitamin K to facilitate the activation of vitamin K-dependent proteins, which are involved in various processes such as blood clotting and calcification. These proteins are activated through the carboxylation of glutamate, a process reliant on the oxygenation of vitamin K hydroquinone (Fasco et al. 1983). The resulting vitamin K epoxide is recycled in two steps: first, it is reduced to vitamin K quinone, followed by its reduction to vitamin K hydroquinone. Recent research has raised questions about whether vitamin K oxidoreductase is directly responsible for the reduction of vitamin K quinone to vitamin K hydroquinone (Rishavy et al. 2013). Recently, vitamin K hydroquinone has been identified as a powerful biological antioxidant (Li et al. 2003). However, no regenerative enzymatic mechanisms for this essential trace nutrient have been discovered yet. The antioxidant property of Vitamin K is known to arise from its hydroquinone form, which is generated through the conversion of its enol-keto forms, thereby exhibiting antioxidant activity (Westhofen et al. 2011).

*Stilbenes* are a group of naturally occurring organic compounds classified as polyphenols. Stilbenes are a small class of phenylpropanoids characterized by a 1,2-diphenylethylene backbone. These compounds belong to a family of secondary metabolites produced via the phenylpropanoid or polyketide pathways. They are composed of a cis- or trans-ethene double bond with each carbon atom of the double bond bonded to a phenyl group. Naturally occurring nucleosides modified by stilbene derivatives may form part of such a system. They are characterized by a central ethene bridge (C=C) connecting two benzene rings, forming a general structure of C6–C2–C6. Trans-stilbenes are more stable and have a linear structure, whereas cis-stilbenes are less stable and exhibit a bent configuration due to steric hindrance. (Table 7).

**Table 7:**
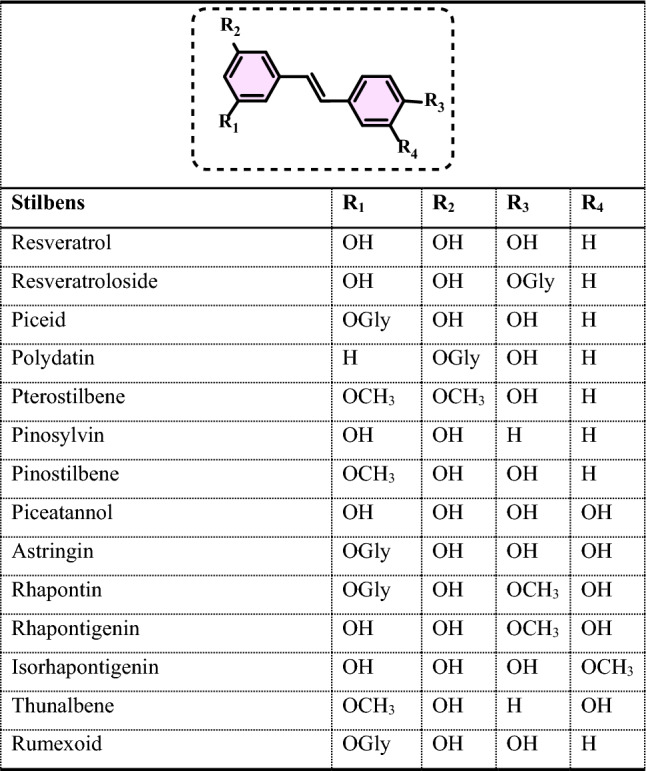
The chemical structure of common plant *trans*-stilbenes (O-Gly: O-β-d-glycopyranoside)

They are synthesized in plants as a defense mechanism against external stresses including pathogenic attack, infection and UV radiation (Teka et al. 2022). From a biochemical perspective, stilbenes encompass basic stilbenes, dihydrostilbenes, (bis)bibenzyls, phenanthrenes, 9,10-dihydrophenanthrenes, and related compounds derived from the general phenylpropanoid pathway (Jeandet et al. 2010). Stilbenes, particularly *trans*-resveratrol and its glycoside, offer significant health benefits due to their antioxidant, anticancer, and antitumor properties (Krawczyk 2019). Most stilbenes and their derivatives are predominantly found in specific plant families (Table 7). Common sources include grape, pine, peanut, and sorghum. In recent years, stilbenes derived from plants have garnered considerable attention for their biological activities and potential pharmacological uses. Among these, resveratrol has been suggested to play a role in health benefits and remains one of the most extensively researched natural products (Chong et al. 2009). Stilbenes are characterized by their strong absorption and fluorescence properties. Various plant families are known to produce stilbenes and their derivatives. For instance, resveratrol has been predominantly identified in 34 plant families, encompassing over 100 species, including peanuts, grapes, bilberries, blueberries, purple grapes, and cranberries (Teka et al. 2022).

*Resveratrol* has garnered considerable global interest due to its capacity to prevent or slow the progression of numerous animal diseases, including cardiovascular diseases and cancer (Crozier, Jaganath and Clifford 2009). It is suggested that resveratrol acts as an antioxidant, inhibits platelet aggregation, promotes nitric oxide production, and enhances high-density lipoprotein cholesterol levels, thereby functioning as a cardioprotective agent. Moreover, resveratrol has been identified as a chemopreventive agent, with extensive experimental efforts focused on understanding this effect. Additionally, resveratrol demonstrates anti-inflammatory, neuroprotective, and antiviral properties (Wolter and Stein 2002; Gülçin 2010). The inhibitory effects of resveratrol on certain metabolic enzymes, including human carbonic anhydrase associated with various diseases, are well documented and thoroughly studied (Innocenti et al. 2010b; Şentürk et al. 2011; Köksal et al. 2020). Furthermore, resveratrol has been shown to possess significant antioxidant activity in various in vitro assays, including total antioxidant activity, reducing power, DPPH^•^, ABTS^•+^, DMPD^•+^, and O_2_^•−^ scavenging, H_2_O_2_ scavenging, and metal chelating activities. When compared to standard antioxidants such as BHA, BHT, and α-tocopherol, resveratrol’s effectiveness is notable (Gülçin 2010b). Additionally, it was observed that resveratrol strongly inhibits carbonic anhydrase I–XV isoforms (Innocenti et al. 2010b; Şentürk et al. 2011) as well as the lactoperoxidase enzyme (Köksal et al. 2020).

### Coumarins

**Fig. 19:**
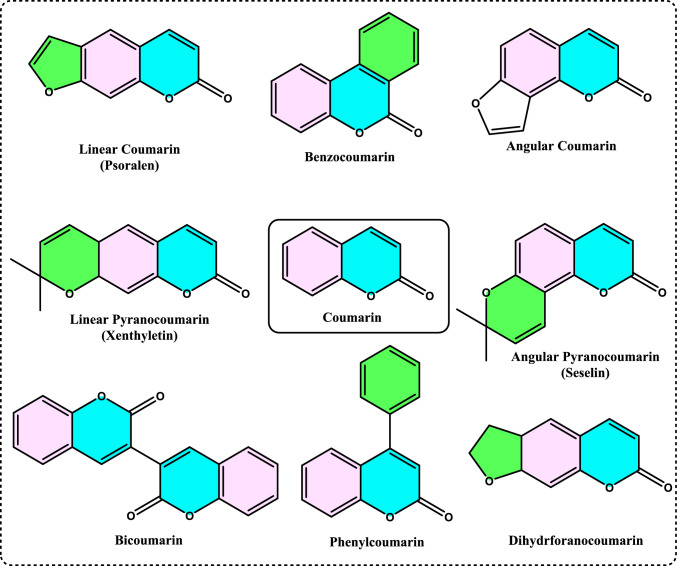
The primary classifications of naturally occurring coumarins

Coumarins (2H-chromen-2-one or benzopyran-2-one) are fragrant and heterocyclic compounds classified under the benzopyrone group, commonly found in various plants, including the seeds of the tonka bean (Garg et al. 2020). Coumarin was first isolated from tonka beans in 1820 by Vogel, who initially misidentified it as benzoic acid. In the same year, 1820, Guibourt also independently extracted coumarin. Unlike Vogel, he correctly identified that the compound was not benzoic acid. It was first synthesized in 1868 by the Perkin. Coumarin is found naturally in many plants including strawberries, apricots, black currants, and cherries. Examples of oxygenated heterocyclic molecules include furan derivatives with four carbon atoms and pyran derivatives with five carbon atoms. While pyran derivatives, which form the backbone of many compounds, are more frequently encountered, furan derivatives are rarely found in plants. Pyran derivatives are ketonic compounds existing as α- or γ-pyrones. The condensation of pyrone derivatives with benzene in plants leads to the formation of secondary metabolites known as coumarin and chromone (Şeker Karatoprak et al. 2024). Coumarins are secondary metabolites found abundantly in higher plants, fungi, bacteria, and sponges (Hussain et al. 2017). Based on the substitutions present on the 1-benzopyran-2-one core structure, coumarins are categorized into various types: simple coumarins, furanocoumarins, pyranocoumarins, and other related compounds (Rastija et al. 2023) (Fig. 19).

The significant structural diversity of these natural compounds and their synthetic derivatives facilitates a broad spectrum of pharmacological activities, including antioxidant properties (Al-Majedy et al. 2016), antibacterial effects, antifungal actions, anti-tubercular effects, anti-HIV activity, cytotoxic properties, and anti-cancer potential (Rastija et al. 2023). Although coumarins do not directly participate in primary physiological processes or regulate growth and reproduction, they play a crucial role in plant defense. The chemical structure of common and simple coumarines are given in Table 8.

**Table 8:**
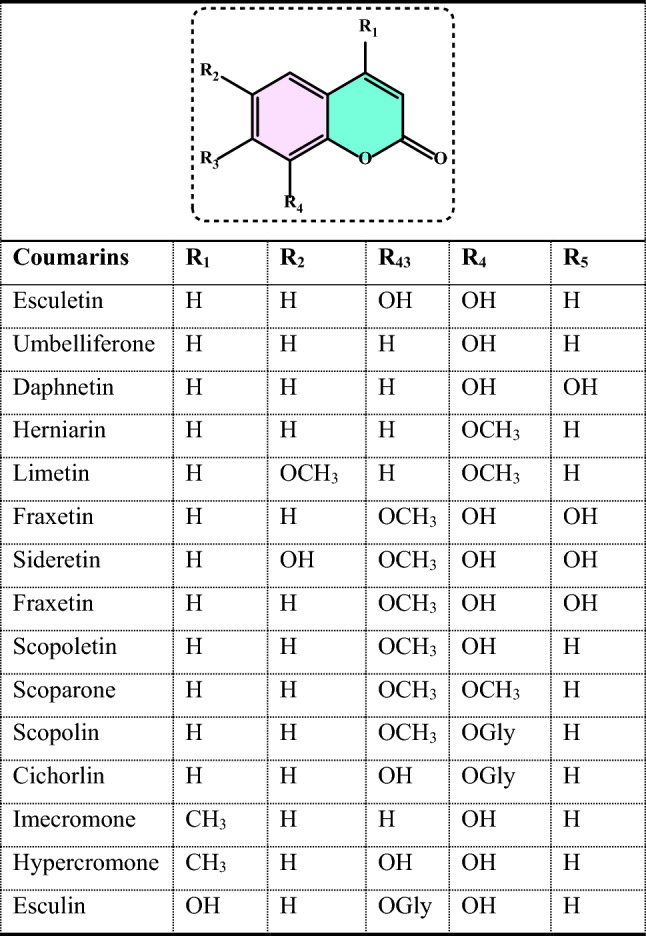
The chemical structure of common and simple coumarines and derivatives

Coumarins and their related analogs naturally occur as secondary metabolites in higher plants and microorganisms (Jayashree et al. 2014). They play a significant role in the proper functioning of various plant components. This group of compounds, derived from both synthetic and natural sources, is valuable in multiple fields. Structurally, coumarins can be described as benzene molecules in which two adjacent hydrogen atoms are replaced by a lactone-like chain, forming a second six-membered heterocyclic ring fused with the benzene ring. Coumarin derivatives from natural and synthetic origins are widely utilized, particularly in medicine, often in the form of glycosides. As an important O-heterocyclic compound, coumarin consists of fused benzene and α-pyrone rings and belongs to the flavonoid family, known for its low toxicity. Recent studies indicate that the biochemical and pharmacological properties of coumarins depend on their substitution patterns, which influence therapeutic applications and can positively affect toxicity. The presence of hydroxyl and amine groups is crucial for the biological activity of many coumarins. The chemical structures of the most common coumarins and derivatives is presented in Table 8 (Skalicka-Woźniak et al. 2016).

Coumarin-based compounds have been found to act through a series of complex pathways to target a wide range of diseases. Their anti-Alzheimer's disease effects are achieved through cholinesterase enzyme inhibition, antihyperglycemic effects through the inhibition of α-glucosidase and α-amylase enzymes, neuroprotective effects by inhibiting monoamine oxidase type B, and anti-inflammatory effects via the inhibition of cyclooxygenase and lipoxygenase enzymes (Seker et al. 2024). Coumarins combat cancer by targeting various enzymes, including protein kinases, sulfatases, aromatases, caspases, and heat shock proteins. This action disrupts processes such as tubulin polymerization, mitosis, and DNA replication, either directly or indirectly (Singh et al. 2018).

Recently, the synthesis of a broad range of coumarins and their derivatives has garnered significant attention from medicinal and organic chemists due to their diverse biological activities, including antitumor, antimicrobial, anti-HIV, serine protease inhibition, anticancer, antioxidant properties, and vasorelaxant effects (Koleva et al. 2019). The pharmacological activities of coumarins largely depend on the specific type of coumarin nucleus. One of the most notable groups of drugs derived from coumarin compounds is vitamin K antagonists, such as warfarin, phenprocoumon, and acenocoumarol, which are used as anticoagulants (Salvo et al. 2017). Coumarins have already achieved clinical importance. Their medical potential was first recognized with the discovery of the anticoagulant activity of dicumarol, which led to the development of synthetic coumarin Warfarin (Coumadin), introduced as a commercial drug in 1954. Warfarin remains the most commonly prescribed oral anticoagulant, with proven efficacy in preventing thromboembolic events (Orhan and Gulcan 2015; Skalicka-Woźniak et al. 2016). Also, it was reported that coumarin frameworks exhibit diverse biological effects such as antimicrobial, antifungal, antibacterial, antiviral, anti-inflammatory, antioxidant, antitumor, antidiabetic, anti-HIV, anticoagulant, anticancer, antiangiogenic, anti-tyrosinase, antituberculosis, antibiotic, anti-allergic, estrogenic, and vasodilatory abilities (Dwivedi et al. 2024). O-glycosides are abundantly found in nature and have diverse applications. Notably, rutin and its derivatives function as vitamins and display various medicinal properties. Linking such sugars with coumarin significantly enhances their distinctive features, resulting in a more intriguing biological profile. Numerous naturally occurring O-coumarin glycosides exist, such as dauroside A, gumoside A, eleutheroside B, and diosfeboside (Mandal 2014; Dwivedi et al. 2024).

### Lignans

**Fig. 20:**
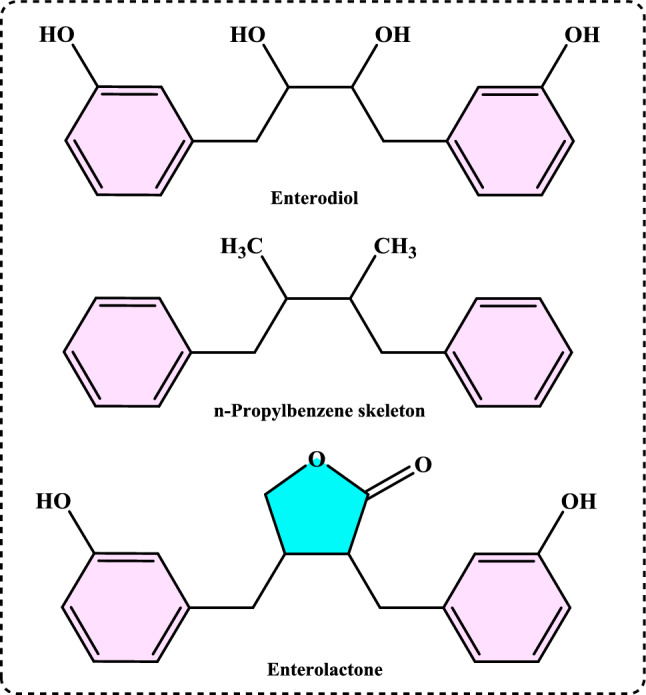
Some mammalian lignans including enterodiol and enterolactone

Lignans are a class of plant-derived polyphenolic compounds found in a variety of foods, including seeds, grains, fruits, and vegetables. They are formed by the dimerization of two phenylpropanoid units and are considered secondary metabolites in plants. Lignans often play roles in plant defense mechanisms against pathogens and environmental stress (Adlercreutz 2007). Lignans are a large group of naturally occurring polyphenols that are derived from the shikimic acid biosynthetic pathway with a wide spectrum of biological functions found abundantly in the plant kingdom and human food sources. Lignans are widely distributed in the plant kingdom, and they exist in plant roots, stems, rhizomes, leaves, fruits, flowers, seeds, resins, and xylem. Until now, lignans are found in over 70 plant families, and more than 100 neolignans and 200 classical lignans have been characterized (Cui et al. 2020). Structurally, they contain a basic scaffold of two or more phenylpropanoid units and the monomers forming lignans are cinnamic acid, propenyl benzene, allyl benzene and cinnamyl alcohol. Lignans are structurally a very diverse group. Up to now, almost two thousands of lignans have been described (Markulin et al. 2019). They are polyphenolic substances derived from phenylalanine via dimerization of substituted cinnamic alcohols to a dibenzylbutane skeleton (2,3-dimethylbutane-1,4-diyl)dibenzene) catalyzed by oxidative enzymes and is often controlled by dirigent proteins. Neolignan is formed by joining the two-propylbenzene residues at other than the β-carbon atom of the propyl side chain. When a part of the human diet, some plant lignans are metabolized by intestinal bacteria to mammalian lignans enterodiol (1,4-bis(3-hydroxyphenyl)butane-2,3-diol) and enterolactone (3,4-bis(3-hydroxybenzyl)dihydrofuran-2(3H)-one) (Fig. 20). In mammals, lignans can easily metabolized to mammalian lignans including pinoresinol, secoisolariciresinol, lariciresinol, matairesinol, syringaresinol, hydroxymatairesinol, and sesamin (Heinonen, et al. 2001). Lignans are generally found as dimers, but some of were found as trimers or tetramers. In plants, most of the lignans are in a free state, while some of them can combine with glycon and form glycosides and other derivatives (Cui et al. 2020).

**Fig. 21:**
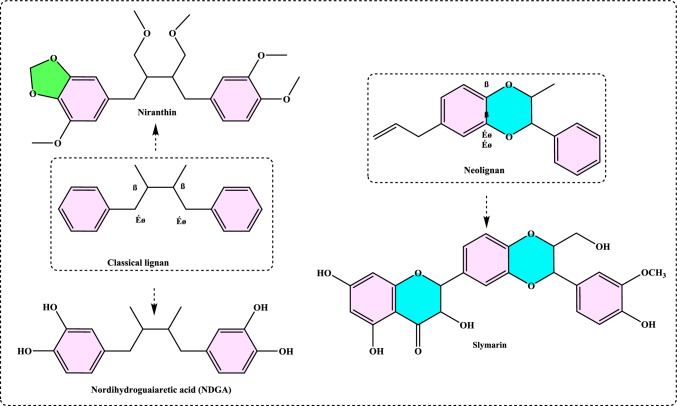
The classification and chemical structures of lignans

Lignans consist of two phenylpropanoid C6-C3 units linked by additional ether, lactone, or carbon bonds at the β and β′ carbons (Davin and Lewis 2000). Lignans are classified as diphenolic compounds and are derived from the biosynthetic pathway of shikimic acid (Hassanein et al. 2024). There are two types of lignans: lignans and neolignans. When the molecular linkage of monomers occurs at the β-β′ positions, the compounds are referred to as “classical lignans”. However, when the main structural units are linked in a different manner, without the β-β′ linkage, they are classified as “neolignans”. Figure 21 illustrates the monomers and the classification of lignans. As shown in the Fig. 21, neolignans have more varied structures than classical lignans (Cui et al. 2020). A prime example of a neolignan is silymarine, which has therapeutic effects such as protection against methotrexate-induced nephrotoxicity, anti-inflammatory, anti-apoptotic, and anti-autophagic properties (Kandemir et al. 2017a, b). Silymarin is a plant-derived flavonoid classified as a benzopyranone. It is isolated from the fruits and seeds of the milk thistle (*Silybum marianum*) and is widely used for the treatment of several diseases. This potential lignan also exhibits antioxidant properties and the ability to inhibit mammalian carbonic anhydrase isoforms (Koksal et al. 2009; Innocenti et al. 2010b).

Lignans are biologically active compounds that are commonly found, recognized, and identified in seeds, fruits, and vegetables and are present in over 70 plant groups. They are dimers of two phenylpropanoid units with varying degrees of oxidation and different substitution patterns on their aromatic structures. They exhibit phytoestrogenic effects. Phytoestrogens are plant-derived compounds that possess estrogen-like properties. The term “phytoestrogens” encompasses two main groups: isoflavones and lignans, both of which are found in significant amounts in the human. Plant lignans encompass various biphenolic compounds, including secoisolariciresinol diglucoside, secoisolariciresinol, pinoresinol, matairesinol, lariciresinol, medioresinol, syringaresinol, sesamin, 7′-hydroxymatairesinol, and isolariciresinol (Smeds et al. 2007). It has been reported that lignans exhibit potent biological activities, including antimalarial, antiparasitic, antioxidant, antibacterial, immunosuppressive, antifungal, and antiasthmatic effects (Neuhaus et al. 2019; Cui et al. 2020), as well as influencing enzymes like carbonic anhydrase, acetylcholinesterase, and butyrylcholinesterase (Polat Köse and Gulcin 2017). There is growing interest in promoting the consumption of lignan-rich foods in human diets. Foods rich in lignans offer health benefits, as breast cancer patients with higher lignan intake through their diet have shown improved survival rates and reduced tumor growth (Ezzat et al. 2018).

### Tannins

**Fig. 22:**
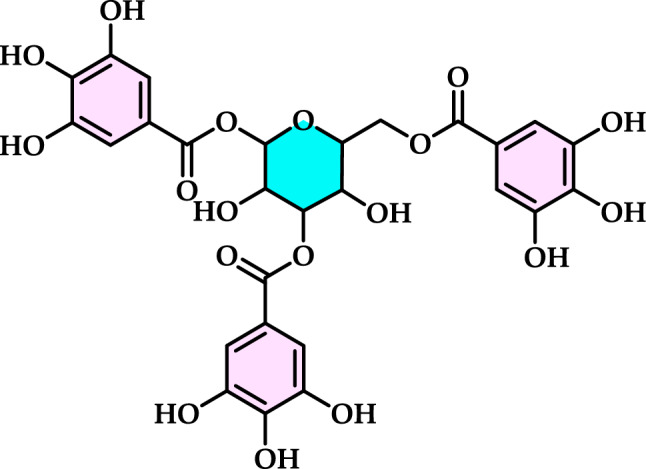
Chemical structure of tannic acid

Tannins are classified as either condensed or hydrolysable proanthocyanidins, based on their chemical structures (Fig. 22). Tannins as naturally occurring water soluble polyphenol compound are high molecular weight molecules (500–3000 Da) that are water-soluble at temperatures between 20 and 35 ℃, except for some complex high molecular weight structures (Marsh et al. 2020). Tannins are defined as polyphenolic compounds that bind to proteins, forming tannin-protein complexes. Additionally, tannins can also bind to saponins, nucleic acids, alkaloids, and polysaccharides such as cellulose, hemicellulose, and pectin. They are localized in plant vacuoles, preventing their inhibitory effects on cellular metabolism (Chaichi Semsari et al. 2011). Additionally, the degradation or modification of tannins over time is important when determining the optimal harvest time. Among phenolic compounds, tannins are essential in regulating plant life during interactions between plants and their environment (Ucar et al. 2013). Tannins are oligomers and polymers of flavonoids, particularly flavan-3-ols, while hydrolysable tannins are glycosylated gallic acid. The phenolic groups in tannins bind tightly to the –NH groups of peptides and proteins, preventing their hydrolysis and digestion in the stomach, and are thus considered anti-nutritional. Proanthocyanidins are mainly found in fruits, especially cocoa and tea, while major sources of hydrolysable tannins include legumes, berries, and leafy vegetables (Shahidi and Naczk 2004; Shahidi and Ambigaipalan 2015).

Tannic acid is a type of polyphenolic compound, specifically a tannin, that is naturally found in various plant sources such as the bark, fruits, and leaves of certain plants. It is a water-soluble molecule consisting of a glucose core with attached galloyl groups. Tannic acid is most commonly found in plants like bananas, raisins, grapes, sorghum, spinach, coffee, persimmons, chocolate, and tea (Gülçin et al. 2010c). Tannic acid is the most notable type of tannins. Its structure consists of a central glucose molecule with ten galloyl groups (Fig. 22). It is a water-soluble polyphenol found in the bark and fruits of various plants, including bananas, raisins, grapes, sorghum, spinach, coffee, persimmons, chocolate, and tea. Tannic acid is commonly used as a food additive, with safe dosage ranges from 10 to 400 μg, depending on the type of food it is added to. It has been shown to have antimutagenic and anticarcinogenic properties. Moreover, it inhibits skin, lung, and forestomach tumors induced by polycyclic aromatic hydrocarbon carcinogens and N-methyl-N-nitrosourea in mice (Gulcin et al. 2010c). Tannic acid has several beneficial properties. It is known for its antioxidant, anti-inflammatory, and potential anticancer effects. It has been shown to inhibit the formation of tumors and can scavenge free radicals such as superoxide, DPPH, and ABTS, helping to reduce oxidative stress. Additionally, tannic acid can chelate metal ions and possess antimicrobial properties. It is often used as a food additive in small quantities and has a wide range of applications due to its biological activity (Gulcin et al. 2020).

## Antioxidant effects

In recent years, the critical role of antioxidants in protecting organisms, tissues, or even nonliving systems from oxidative stress has become increasingly clear. Evidence for this is provided by numerous studies across diverse fields, including physiology, pharmacology, nutrition, and food processing (Magalhaes et al. 2009). In the context of food, antioxidants can be defined as substances capable of slowing, delaying, or preventing the onset of rancidity or other flavor degradation caused by oxidation. They achieve this by extending the induction period, thereby delaying the development of undesirable flavors. However, adding antioxidants after the induction period has ended is generally ineffective in preventing rancidity (Taslimi et al. 2018a).

Antioxidants combat oxidation in two primary ways: either by neutralizing free radicals, in which case they are referred to as primary antioxidants, or through mechanisms that do not involve direct free radical scavenging. Primary antioxidants include phenolic compounds like α-tocopherol (Altay et al. 2019), which are depleted during the induction period. Secondary antioxidants work through various mechanisms, such as chelating metal ions, scavenging reactive oxygen species, converting hydroperoxides into non-radical species, absorbing UV radiation, or quenching singlet oxygen. Secondary antioxidants typically exhibit activity only in the presence of additional minor components. For example, chelating agents like citric acid are effective only when metal ions are present, while reducing agents such as ascorbic acid require primary antioxidants like tocopherols to function effectively (Gulcin et al. 2008a).

**Fig. 23:**
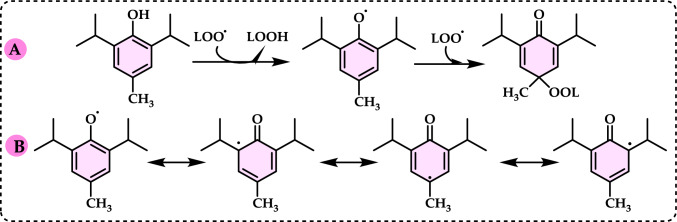
Reaction products of a hindered phenol (BHT) during autoxidation **(A)**, resonance stabilization of phenoxy radical **(B)**

The effectiveness of antioxidants is influenced not only by their structural characteristics, such as their chemical reactivity towards peroxyl and other reactive species, but also by numerous other factors. These include concentration, temperature, light exposure, the type of substrate, the physical state of the system, and the presence of various microcomponents that may act as prooxidants or synergists. It has been observed that the involvement of an antioxidant in side reactions during chain initiation and propagation can reduce its effectiveness. For instance, peroxydienones (Fig. 23A), formed as reaction by-products, limit the utility of BHT at high temperatures or under UV light because they generate new free radicals that perpetuate the kinetic chain reaction (Gülçin 2012). Additionally, the resonance stabilization of the phenoxy radical is illustrated in Fig. 23B.

**Fig. 24:**
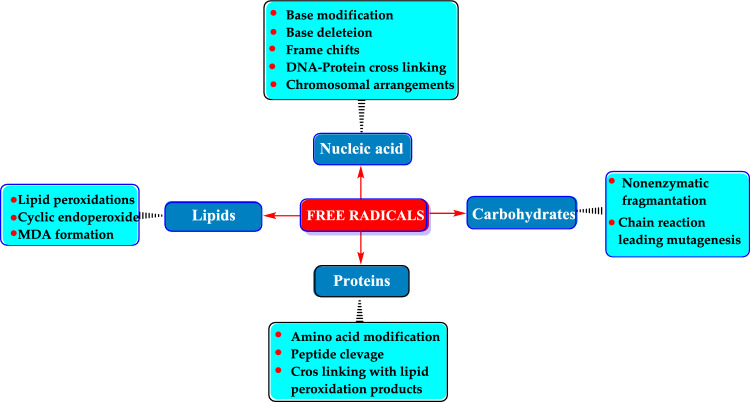
The biomolecules targeted by free radicals and the molecular damage caused by free radicals

To the best of our knowledge, ROS and RNS, such as superoxide, hydrogen peroxide, singlet oxygen, and other free radicals like nitrogen radicals, are generated in the human body due to various internal sources or external factors. Accumulating evidence suggests that ROS, including ROO·, HO·, O_2_·^−^, and ^1^O_2_, are involved in the pathophysiology of aging and numerous diseases such as cancer, Alzheimer's disease, and Parkinson’s disease (Finkel and Holbrook 2000; Turkan et al. 2020). ROS and other free radicals are natural by-products of cellular metabolism in aerobic organisms, where molecular oxygen is prevalent. They are generated through processes such as UV light irradiation, exposure to X-rays and gamma rays, metal-catalyzed reactions, atmospheric pollutants, inflammatory responses by neutrophils and macrophages, and as by-products of mitochondrial electron transport reactions and other mechanisms (Cadenas 1989). It has been reported that ROS can cause oxidative damage to lipids, proteins, nucleic acids, and polyunsaturated fatty acids in living cells. It is well known that free radicals or ROS play critical roles in the development of various chronic diseases, including aging, heart disease, and cancer (Gülçin et al. 2006a). Over the past years, the role of ROS and RNS in the oxidative deterioration of food products and the pathogenesis of several human diseases such as atherosclerosis, diabetes mellitus, chronic inflammation, neurodegenerative disorders, and certain types of cancer has been well established (Valko et al. 2006). It is crucial for health to find effective ways to reduce or suppress the formation of free radicals in the body. Oxidative stress is defined as an imbalance between the production of ROS and the body's antioxidant defenses (Buldurun et al. 2020). When the balance of prooxidant and antioxidant reactions is disrupted, ROS are overproduced, leading to oxidative stress that impairs the normal functions of cellular lipids, proteins, DNA, and RNA. As a result, there has been increasing interest in discovering natural antioxidant compounds from herbal medicine that can effectively neutralize free radicals. The protective effects of antioxidants against oxidative damage have garnered significant attention in fields such as biology, medicine, nutrition, and agrochemistry (Magalhaes et al. 2008). While oxidants and reductants are chemical terms, in biological systems, they are often referred to as prooxidants and antioxidants, respectively (Cao and Prior 1998). Prooxidants are substances that can cause oxidative damage to various biological targets, including carbohydrates, nucleic acids, lipids, and proteins (Fig. 24).

On the other hand, antioxidants are substances that can efficiently reduce pro-oxidants, resulting in the formation of products with little or no toxicity. However, the best definition of an antioxidant was proposed by Halliwell and colleagues in 1995. According to them, an antioxidant is a substance that, when present in low concentrations compared to an oxidizable substrate, significantly delays or prevents the oxidation of that substrate. This protection can occur through various mechanisms, including: inhibition of generation and scavenging capacity against ROS or RNS; reducing capacity; metal chelation; singlet oxygen quenching activity; and hydrogen peroxide decomposition. Antioxidants serve as a defensive factor against the harmful effects of free radicals in the body. They not only eliminate ROS but also regulate the cellular redox state and enable redox signal transduction. They work by inhibiting the initiation and propagation steps of oxidation, leading to the termination of the reaction and slowing down the oxidation process (Gulcin 2006a). Currently, a variety of synthetic antioxidants are widely used. However, the use of these compounds has been restricted by regulations due to concerns about their toxicity and carcinogenic effects. Plant foods are potential sources of natural antioxidants, such as vitamin C, α-tocopherol, carotenoids, flavonoids, and phenolic acids, which help prevent free radical damage. They provide a phenolic hydroxyl group that reacts with free radicals, thereby inhibiting oxidative mechanisms that contribute to degenerative diseases. As a result, there is growing interest in natural and safer antioxidants for food applications, along with a rising consumer preference for natural antioxidants. This has led to increased efforts to explore natural sources of antioxidants (Gülçin 2006b; 2007; 2010). In recent years, a variety of spectrophotometric assays have been adopted to measure the antioxidant capacity of foods and pharmaceuticals. As a result, it is important to establish and standardize methods that can directly measure the antioxidant levels in plant-based materials, food extracts, and biological samples. The standardization of antioxidant methods requires agreement on several key points (Apak et al. 2022):

- The general framework for antioxidant assays should include test conditions, a detailed description of the procedure, the experimental apparatus, and the stability of the reagents used.
- All antioxidant methods must be validated within their specific framework, including validation parameters such as repeatability, reproducibility, recovery data, internal quality control, standardization, proficiency testing, and analytical quality assurance.
- The results should provide a reliable comparison of the antioxidant content in pharmaceuticals, foods, and other commercial products.
- The methods should be refined to meet quality standards for legal and health-related requirements.

Currently, the most commonly used methods for in vitro determination of antioxidant capacity in food components include the inhibition of lipid peroxidation in the linoleic acid system, total radical-trapping antioxidant parameters (TRAP assay), oxygen radical absorbance capacity (ORAC assay), ferric ion (Fe^3+^) reducing antioxidant power (FRAP), cupric ion (Cu^2+^) reducing antioxidant power (CUPRAC assay), Fe^3+^-Fe^2+^ transformation assay, Folin–Ciocalteu reducing capacity (FCR assay), DPPH^•^ radical scavenging, ABTS^•+^ radical scavenging, DMPD^•+^ radical scavenging, superoxide anion radical scavenging, and ferrous ion (Fe^2+^) chelation activities. Most of these methods are based on the same principle: a synthetic colored radical or redox-active compound is generated, and the ability of a biological sample to scavenge the radical or reduce the redox-active compound is measured using a spectrophotometer. An appropriate standard is applied to quantify the antioxidant capacity (Floegel et al. 2011). These methods are recommended to measure the antioxidant properties of food components, as they better reflect their potential protective effects.

The main objective of this overview is to review the chemical principles behind these methods, discuss some of their variations, explore recent applications, and highlight their advantages and shortcomings. Another aim of this review is to provide a comprehensive guide to the chemical investigation methods available for antioxidant assays. There is a substantial body of research on antioxidant assay methodology and the mechanistic steps involved. In this review, I have made an attempt to bridge the gap between the reaction mechanisms of antioxidants and the methodologies used in their analysis.

## Antioxidant methods

The various techniques and instruments used to measure antioxidant activity have significantly advanced over the past few decades. These methods have been developed to measure and investigate the antioxidant properties and capacities of commercial antioxidants, foods, medicinal products, pharmaceuticals, and biological samples. The concept of antioxidant capacity initially emerged in chemistry and was later applied to fields such as biology, medicine, epidemiology, and nutrition (Floegel et al. 2011). One of the goals of this review article is to compile all possible methods used to evaluate the antioxidant properties of different samples. In recent decades, there has been a growing interest in studying the antioxidant activity of foods and diets, due to the recognized role of oxygen free radicals in the progression of cardiovascular diseases, neurodegenerative disorders, aging, and cancer (Garcia-Parrilla 2008). To this end, various procedures have been developed to test the total antioxidant capacity of food products (Perez-Jimenez and Saura-Calixto 2005). It has been reported that reliable methods for antioxidant assessment are necessary (Magalhaes et al. 2008). A standardized method for evaluating the antioxidant activity of food components should meet the following ideal criteria (Prior et al. 2005):

- It measures the chemistry that actually occurs in potential applications.
- It uses a biologically relevant radical source.
- It should be simple.
- It should employ a method with a defined endpoint and chemical mechanism.
- The chemicals and instrumentation used should be easily accessible.
- It should show good reproducibility within a single run and between different days.
- It should be adaptable for assessing both hydrophilic and lipophilic antioxidants and for using different radical sources.
- It should be suitable for high-throughput analysis in routine quality control.

Moreover, antioxidant activity should not be concluded from a single antioxidant test model. Multiple in vitro antioxidant procedures should be performed to assess the antioxidant activities of the samples of interest (Alam et al. 2013a, b). Another important consideration is that antioxidant tests can differ in various ways. As a result, it can be challenging to compare one method to another. Researchers must critically evaluate the methods of analysis before selecting the most appropriate one for their research (Askin et al. 2018). The ideal method for determining antioxidant properties should assess the effect of food compounds under conditions that replicate those encountered when oxidative stress is induced in vivo by ROS and RNS. However, such assessments may be considered overemphasized for screening purposes, especially when testing against numerous ROS or RNS (Magalhaes et al. 2009). On the other hand, methods can be classified based on reaction mechanisms into hydrogen atom transfer (HAT) and single electron transfer (SET) methods.

Generally, methods for determining the antioxidant capacity of food components can deactivate radicals through two main mechanisms, and are classified into two major groups: assays based on the SET reaction and assays based on HAT. The final result is the same, regardless of the mechanism, but the kinetics and the potential for side reactions differ. SET-based methods measure the ability of a potential antioxidant to transfer a single electron, which reduces various compounds, including metals, carbonyls, and radicals. This SET reaction is observed through a color change as the oxidant is reduced (Huang et al. 2005; Apak et al. 2022). On the other hand, HAT-based methods assess the classic ability of an antioxidant to neutralize free radicals by donating a hydrogen atom. HAT reactions are independent of solvent and pH and are usually very fast, typically completed within seconds to minutes. The presence of reducing agents, including metals, can complicate HAT assays and lead to falsely high apparent reactivity (Prior et al. 2005). In HAT-based methods, antioxidants quench free radicals by donating hydrogen atoms.

$${\text{AH}} + {\text{ X}} \cdot { } \to {\text{A}} \cdot + {\text{ XH}}$$

The relative reactivity of these methods is determined by the bond dissociation energy of the H-donating group of the antioxidant, which is characteristic for compounds with a bond dissociation energy interval of approximately − 10 kcal/mol and an ionization potential of less than − 36 kcal/mol. These reactions are independent of pH and solvent, and are very fast, typically completed in seconds to minutes. The presence of reducing agents, such as metal ions, is not recommended in such methods as it can result in apparently higher reactivity (Prior et al. 2005). Methods based on the HAT reaction include the following (Huang et al. 2005):

- Oxygen radical absorbance capacity (ORAC)
- Total radical trapping antioxidant parameter (TRAP)
- Inhibition of induced LDL oxidation
- Total radical scavenging capacity assay (TOSCA)
- β-Carotene bleaching assays
- Chemiluminescent assay

SET-based methods measure the ability of a potential antioxidant to transfer one electron, reducing various compounds, including metal ions, carbonyl groups, and radicals (Wright et al. 2001). SET and HAT mechanisms almost always occur together in all samples, with the balance between them determined by the antioxidant structure and pH. SET-based methods detect the ability of an antioxidant to transfer one electron to reduce any compound, including metals, carbonyls, and radicals.

$${\text{AH}} + {\text{ X}} \cdot { } \to {\text{AH}}^{ \cdot + } + {\text{X}}^{ - }$$

$${\text{AH}}^{ \cdot + } + {\text{ H}}_{2} {\text{O}} \to {\text{ A}}^{ \cdot } + {\text{H}}_{3} {\text{O}}^{ + }$$

$${\text{X}}^{ - } + {\text{ H}}_{3} {\text{O}}^{ + } \to {\text{ XH}} + {\text{H}}_{2} {\text{O}}$$

$${\text{AH}} + {\text{ M}}^{3 + } \to {\text{ AH}}^{ + } + {\text{M}}^{2 + }$$

Relative reactivity in SET methods primarily depends on deprotonation (Lemanska et al. 2001) and the ionization potential of the reactive functional group (Wright et al. 2001). Therefore, SET reactions are pH-dependent. In general, ionization potential values decrease with increasing pH, reflecting an enhanced electron-donating capacity with deprotonation. A correlation between redox potential and SET methods has been suggested (Ou et al. 2005), though it has not been consistently demonstrated. SET reactions tend to be slow and may require a long time to reach completion, so antioxidant capacity calculations are typically based on the percentage decrease in product rather than kinetics. When AH + has a sufficiently long lifetime, secondary reactions may interfere significantly in assays, potentially leading to toxicity or mutagenicity in vivo (Sartor et al. 1999). SET methods are very sensitive to ascorbic acid and uric acid, which are important for maintaining plasma redox balance, and can also detect reducing polyphenols. Notably, trace components and contaminants, such as metals, can interfere with SET methods and contribute to high variability and poor reproducibility and consistency in results (Ou et al. 2005).

The relative reactivity of the SET method is determined by deprotonation and the ionization potential of the reactive functional group. As such, SET methods are pH-dependent. Generally, ionization potential decreases with increasing pH, which reflects a higher electron-donating capacity with deprotonation. The antioxidant mechanism is predominantly of the electron transfer type when ionization potential values are greater than − 45 kcal/mol. Reactions based on electron transfer are usually slow, and antioxidant capacity calculations are based more on the percentage decrease in the product than on kinetic parameters (Prior et al. 2005). The following assays are SET-based methods:

- Total phenolics assay using Folin–Ciocalteu reagent
- Trolox equivalent antioxidant capacity assay (TEAC)
- Ferric ion reducing antioxidant power assay (FRAP)
- Total antioxidant potential assay using a Cu^2+^ complex as an oxidant
- 2,2-Diphenyl-1-picrylhydrazyl radical scavenging assay (DPPH·)
- 2,2-Azinobis 3-ethylbenzthiazoline-6-sulfonic acid radical scavenging assay (ABTS^•+^)
- N,N-Dimethyl-p-phenylenediamine radical scavenging assay (DMPD^•+^)
- Cupric ion (Cu^2+^) reducing antioxidant power assay (CUPRAC)

It has been reported that ABTS methods involve both HAT and SET mechanisms (Prior et al. 2005). Other assays that measure a sample’s scavenging ability against oxidants, which interact with and damage major macromolecules in biological systems or foodstuffs, include (Huang et al. 2005; MacDonald-Wicks et al. 2006; Miguel 2010):

- Superoxide anion radical (O_2_·^−^) scavenging assays
- Hydroxyl radical (HO·) scavenging assays
- Singlet oxygen (^1^O_2_) quenching assays
- Peroxynitrite (ONOO^−^) scavenging assays
- Hydrogen peroxide (H_2_O_2_) scavenging assays

### Total antioxidant capacities

#### Thiocyanate methods

Total antioxidant capacity is commonly employed as a measure to evaluate the bioactivity of food and medicinal components. This assay refers to a compound’s capability to prevent oxidative damage, such as lipid peroxidation (Roginsky and Lissi 2005). Lipid peroxidation involves the oxidative breakdown of lipids containing carbon–carbon double bonds. Typically, to assess the potential impact of a bioactive compound, its ability to inhibit the peroxidation of a linoleic acid emulsion is tested. During linoleic acid oxidation, linoleic acid hydroperoxides are formed, which subsequently degrade into secondary oxidation products. The thiocyanate method is an analytical technique used to measure antioxidant activity by detecting the ability of antioxidants to scavenge free radicals. This method typically involves the reaction of antioxidants with specific radicals, such as peroxyl or hydroxyl radicals, resulting in the formation of stable products that can be measured spectrophotometrically (Gülçin 2020). This antioxidant method is employed to quantify peroxides at the early stage of lipid peroxidation. During the oxidation of linoleic acid, peroxides are generated, which react with Fe^2^⁺ to produce Fe^3^⁺. The Fe^3^⁺ ions then form a complex with thiocyanate (SCN⁻), exhibiting maximum absorbance at 500 nm. The formation of Fe^3+^ and thiocyanate complex is measured by spectrophotometry, providing a quantifiable indicator of antioxidant capacity. When antioxidants are present, the oxidation of linoleic acid proceeds at a slower rate. Consequently, the color development resulting from thiocyanate formation also occurs more slowly (Gülçin 2002). In particular, the thiocyanate method often focuses on assessing the ability of antioxidants to measurement of formation of Fe^3+^ and thiocyanate complex, which is indicative of the antioxidant’s effectiveness. This method is used to assess the antioxidant capacity of various substances, including plant extracts, foods, and pharmaceuticals. By measuring the concentration of thiocyanate or other related products, researchers can quantify the antioxidant potential of the sample being studied (Huang et al. 2005).

Lipid peroxidation begins when a radical targets the side chain of a fatty acid, abstracting a hydrogen atom from a methylene carbon. Fatty acids with a higher number of double bonds are more susceptible to this process, as the removal of hydrogen atoms becomes easier with increased unsaturation. As a result, polyunsaturated fatty acids are more prone to radical-induced oxidation compared to monounsaturated and saturated fatty acids, which demonstrate greater resistance to such attacks (Carocho and Ferreira 2013). Lipid peroxidation refers to a series of biochemical events triggered by the action of ROS on the unsaturated fatty acids in cellular and subcellular membranes. This process disrupts membrane integrity, fluidity, and selectivity, while also generating various lipid radicals that further exacerbate oxidative damage (Gill and Tuteja 2010; Sharma et al. 2012; Anjum et al. 2015; Halliwell and Gutteridge 2015; Singh et al. 2018). The occurrence of lipid peroxidation is closely associated with oxidative stress, as the lipid-derived free radicals formed during this process can also interact with other macromolecules, such as DNA and proteins (Soares et al. 2019).

**Fig. 25:**
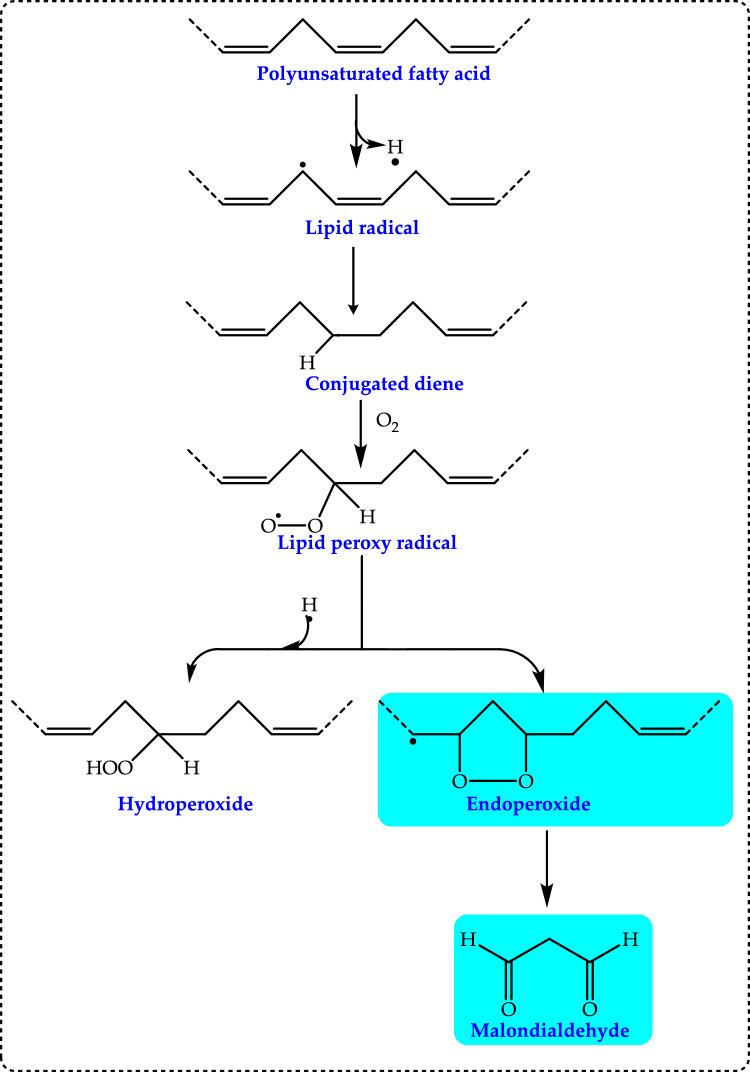
Reaction of 12-hydroperoxide from α-linolenic acid to form 9-hydroperoxy endoperoxide

Lipid peroxidation is recognized as a primary degradation process that significantly impacts both the sensory attributes and nutritional value of foods, particularly lipid-rich products (Yanishlieva and Marinova 2001). In biological systems, it is considered a harmful process associated with various pathological outcomes (Hochstein and Atallah 1988). Lipid peroxidation leads to the formation of lipid hydroperoxides, which can disrupt membrane fluidity and impair the functionality of membrane proteins. Moreover, lipid hydroperoxides can undergo one-electron reduction mediated by iron, followed by oxygenation, resulting in epoxyallylic ROO· radicals. These radicals initiate a chain reaction of lipid peroxidation, driven by free radicals. The process produces reactive aldehydes, such as 4-hydroxy-2-nonenal and malondialdehyde, which are highly cytotoxic (Yu and Yang 1996). These reactive aldehydes can further damage other cellular components, including proteins and DNA, propagating the oxidative harm initiated within cellular membranes to other critical macromolecules. Since lipid hydroperoxides in membranes are key contributors to reactive oxygen species (ROS) production in vivo, their detoxification is essential for cellular survival under oxidative stress conditions (Dargel 1992). Antioxidants play a crucial role in mitigating lipid peroxidation and protecting cells from free radical-induced damage. To evaluate antioxidant activity, various assay methods have been developed for use in both food systems and biological samples. Polyunsaturated fatty acids, such as linoleic acid, can undergo further oxidative reactions to produce dihydroperoxides and oxygen-containing cyclic structures, including hydroperoxy epidioxides and bicycloendoperoxides. The autoxidation mechanism of a polyunsaturated fatty acid is depicted in Fig. 25.

Lipid peroxidation involves a series of chain reactions mediated by free radicals and is linked to various types of biological damage. The thiocyanate method quantifies the peroxides formed during the early stages of oxidation, which are the primary products of lipid oxidation. This assay indirectly measures the hydroperoxides generated from the auto-oxidation of a linoleic acid emulsion over the experimental period. Ferrous chloride reacts with thiocyanate in the presence of hydroperoxides to produce ferrous thiocyanate (Inatani et al. 1983; Gulcin 2012). Lipid peroxidation occurs during the harvesting, storage, and processing of foods, leading to chemical spoilage, which manifests as rancidity and degradation in the nutritional value, flavor, color, safety, and texture of food and pharmaceutical products. It also contributes to the quality deterioration of dairy products, meats, vegetables, and fruits. Moreover, it damages cellular components such as membrane lipids, proteins, enzymes, and DNA, thereby disrupting cellular integrity. To counteract lipid oxidation, food manufacturers commonly use antioxidants as an effective strategy for stabilizing food lipids and preserving the quality of food products (Shahidi and Zhong 2015). Over the years, as molecular and biochemical studies on stress responses have advanced, lipid peroxidation has become widely recognized as a key indicator of oxidative damage. Typically, lipid peroxidation is evaluated by measuring membrane ion leakage or quantifying one of its byproducts, MDA. When MDA reacts with thiobarbituric acid, it forms a colored compound, the intensity of which can be measured at 532 nm (Soares et al. 2019).

#### Phosphomolybdenum method

The phosphomolybdenum method is a quantitative assay used to evaluate the antioxidant capacity of a sample. This method is based on the reduction of Mo^6+^ to Mo^5+^ by antioxidants under acidic conditions, forming a green-colored phosphate/Mo^5+^complex. The complex has a maximum absorbance at 695 nm, which can be measured using a spectrophotometer to determine the antioxidant activity. The assay is based on the reduction of Mo^6+^ to Mo^5+^ by the sample analyte and subsequent formation of a green phosphate Mo^5+^ complex at acidic pH (Alam et al. 2013a, b). This reduction results in the formation of a phosphomolybdenum complex, which gives a green color. The intensity of the green color is proportional to the total antioxidant capacity of the sample. In this method, the sample containing the antioxidant solution is mixed with the phosphomolybdenum reagent, which consists of 0.6 M sulfuric acid, 28 mM sodium phosphate, and 4 mM ammonium molybdate. The mixture is then capped and incubated in a boiling water bath at 95 °C for 90 min. Once the sample has cooled to room temperature, the absorbance of the aqueous solution is measured at 695 nm using a UV spectrophotometer, with a blank solution as a reference. The blank contains the reagent solution and the corresponding volume of the solvent used for the sample, and is incubated under the same conditions as the sample. For samples of unknown composition, the antioxidant capacity can be expressed as equivalents of α-tocopherol or trolox (Prieto et al. 2012).

#### β-Carotene bleaching assay

The β-carotene bleaching assay is a widely used method to evaluate the antioxidant activity of substances, primarily to measure their ability to inhibit lipid peroxidation. This assay is based on the principle that β-carotene, a carotenoid and natural antioxidant, undergoes oxidation (bleaching) when exposed to ROS, which are generated during the oxidation of unsaturated fatty acids like linoleic acid (Gulcin 2020). This method is one of the oldest and most widely used techniques for assessing lipid peroxidation in oil-in-water emulsions. This method is a rapid and straightforward approach for screening antioxidant effects, based on the principle that linoleic acid, an unsaturated fatty acid, undergoes oxidation due to ROS produced by oxygenated water. In the assay, β-carotene dissolved in chloroform is added to a linoleic acid emulsion. The chloroform is then evaporated at 40 ℃, and distilled water saturated with oxygen is slowly added to the residue, followed by vigorous agitation to form a stable emulsion. An aliquot of this mixture is then transferred into test tubes containing the sample dissolved in methanol at the desired final concentrations. β-carotene is dissolved in chloroform and added to a linoleic acid emulsion. The chloroform is then evaporated, and oxygenated water is added to form a stable emulsion. The oxidative degradation of the emulsion, which includes β-carotene, linoleic acid, and water, is monitored spectrophotometrically both in the presence and absence of the antioxidant samples. The oxidation process leads to the bleaching of β-carotene, and this discoloration is tracked by measuring absorbance at 470 nm. The tubes are incubated at 50 ℃ for two hours, allowing radicals generated by the spontaneous oxidation of linoleic acid to be enhanced by thermal induction. These radicals cause β-carotene bleaching, which is slowed down by antioxidants. The reaction is carried out in an aqueous emulsion of β-carotene and linoleic acid using Tween 40 as a surfactant. The presence of an antioxidant compound retards the bleaching of β-carotene. However, some limitations of this assay include potential inaccuracies in quantification, low reproducibility, reagent preparation complexity, and interference from factors like pH, temperature, and solvents (Alam et al. 2013a, b; Apak et al. 2022). The mechanism involves the formation of ROO·radicals from the thermal degradation of an initiator in the presence of oxygen. These ROO·radicals can bleach β-carotene either by abstracting a proton from radical scavengers or by directly reacting with the polyene system of β-carotene. Hydroxyl and azyl radicals are not typically assayed due to their high reactivity, and superoxide and alkyl radicals are unsuitable because of their low reactivity (Shivakumar and Kumar 2018). Antioxidants present in the sample can inhibit or slow down the oxidation process, thereby reducing the bleaching of β-carotene. This results in a slower decrease in absorbance and indicates antioxidant activity (Gulcin 2020).

### Oxygen radical absorbance capacity (ORAC assay)

The Oxygen Radical Absorbance Capacity (ORAC) is a groundbreaking test tube method designed to evaluate the antioxidant potential of foods and other substances. This assay measures the ability of antioxidants to break radical chains by observing the inhibition of oxidation induced by peroxyl radicals (ROO·). Unlike other methods that measure either inhibition time at a fixed degree or the inhibition degree at a fixed time, ORAC uses an area-under-curve approach, integrating both the time and degree of radical inhibition into a single measurement (Miller et al. 1993; Whitehead et al. 1992). ORAC reflects the classic radical chain-breaking activity of antioxidants through HAT by assessing their ability to inhibit ROO·-induced oxidation (Ou et al. 2001). In the basic assay, ROO· reacts with a fluorescent probe, producing a nonfluorescent product. The antioxidant capacity is determined by measuring the reduced rate and amount of product formation over time through fluorescence:

$${\text{R}} - {\text{N}} = {\text{N}} = {\text{R }}\to ^{{{\text{O}}_{2} }} {\text{ N}}_{2} + { }2{\text{ROO}}^{ \bullet }$$

$${\text{ROO}}^{ \bullet } { } + {\text{ Probe}}_{{\left( {{\text{Fluorescent}}} \right)}} \to {\text{ROOH }} + {\text{ Probe}}_{{\left( {{\text{Non}} - {\text{fluorescent}}} \right)}}$$

$${\text{AH}} + {\text{ ROO}} \cdot { } \to {\text{ROOH}} + {\text{A}} \cdot$$

$${\text{A}} \cdot + {\text{ ROO}} \cdot { } \to {\text{ROO}} - {\text{A}}$$

The principle of this assay relies on measuring the decrease in fluorescence intensity of a fluorescent molecule, such as β-phycoerythrin or fluorescein, over time under a constant and reproducible flux of ROO·, which is generated by the thermal decomposition of 2,2′-azobis(2-amidino-propane) dihydrochloride (AAPH) in an aqueous buffer. This assay involves generating free radicals using AAPH and monitoring the reduction in fluorescence in the presence of free radical scavengers (Alam et al. 2013a, b). AAPH is currently the only free-radical generator used in the ORAC assay. Various foods and food constituents have been tested using this method. When a sample containing chain-breaking antioxidants is present, the decay of fluorescence is inhibited. Initially, the fluorescent probe β-phycoerythrin, isolated from *Porphyridium cruentum*, was used as it reacts with ROO· to form a non-fluorescent product (Cao et al. 1993). However, more stable and less reactive fluorescent probes, such as fluorescein (Ou et al. 2001) or dichlorofluorescein, are now preferred. The use of β-phycoerythrin in antioxidant assays has some limitations for several reasons (Cao and Prior 1998).

- β-phycoerythrin exhibits significant variability in its reactivity to ROO·, which can lead to inconsistent results in the assay.
- β-phycoerythrin undergoes photobleaching after exposure to excitation light and interacts with polyphenols through non-specific protein binding.
- Proanthocyanidins, particularly, bind to β-phycoerythrin through non-specific protein interactions.

Both of these factors contribute to falsely low ORAC values. To address these issues, the synthetic, non-protein fluorescent probe fluorescein has been used instead of the original β-phycoerythrin. The oxidized fluorescein products induced by ROO· have been identified by LC–MS, and the reaction mechanism has been confirmed as a classic HAT mechanism (Ou et al. 2001). The probe’s reaction with ROO· results in a loss of fluorescence over time. Antioxidant analyses typically focus on extending the lag phase, although antioxidant effects often extend well beyond the initial stages of oxidation (Ou et al. 2005). While fluorescent markers are sensitive, they require detection with fluorometers, which may not be routinely available in analytical laboratories, though they are commonly used in many cell culture labs. The long analysis time has also been a major limitation, but this has been partially addressed with the development of high-throughput assays (Huang et al. 2002a).

The ORAC assay provides a controllable source of ROO·, modeling antioxidant reactions with lipids in both food and physiological systems. It can be adapted to detect both hydrophilic and hydrophobic antioxidants by modifying the radical source and solvent (Ou et al. 2002a, b). The assay for both lipophilic and hydrophilic chain-breaking antioxidants uses a mixture of acetone or water containing 7% randomly methylated β-cyclodextrin to enhance water solubility (Huang et al. 2002b). Lipophilic compounds are also quantified using ORAC with either organic media or liposomes (Naguib 1998). To enhance this assay, Huang et al. developed a high-throughput method using a multichannel liquid handling system coupled with a microplate fluorescence reader in a 96-well format (2002b). Small temperature differences in the outer wells of the microplate can reduce the assay’s reproducibility. This is not exclusive to the ORAC assay but applies to any assay that is highly sensitive to temperature and uses microplates and microplate readers (Ou et al. 2001).

Additionally, one advantage of using the ORAC method to evaluate antioxidant capacity in food substances is that it accounts for samples with or without lag phases in their antioxidant effects. This is particularly useful for measuring foods and supplements containing complex ingredients with various slow and fast-acting antioxidants, as well as ingredients with combined effects that cannot be pre-calculated. Several modified ORAC methods have been proposed. Most employ the same principle, but the electron paramagnetic resonance-based ORAC (ORAC-EPR) directly measures the decrease in AAPH radical levels due to antioxidant scavenging activity (Kohri et al. 2009). In conclusion, this assay measures the oxidative degradation of a fluorescent molecule, such as β-cyclodextrin or fluorescein, after it has been mixed with free radical generators, such as azo-initiator compounds. Azo-initiators generate ROO· by heating, which damages the fluorescent molecule, causing a loss of fluorescence. The fluorescence intensity decreases as oxidative degeneration progresses, and this intensity is typically recorded for half an hour after adding the azo-initiator as the free radical generator. Antioxidants help protect the fluorescent molecule from oxidative degeneration, and the degree of protection is quantified using a fluorometer. Fluorescein is currently the most commonly used fluorescent probe, and commercially available equipment can automatically measure and calculate antioxidant capacity.

### Total radical-trapping antioxidant parameter (TRAP assay)

The TRAP assay is based on the protection provided by antioxidants against the fluorescence decay of R-phycoerythrin during a controlled peroxidation reaction. The fluorescence of R-phycoerythrin is quenched by AAPH, which acts as a radical generator. This quenching reaction is measured in the presence of antioxidants. The antioxidative potential is evaluated by measuring the decay in decoloration. TRAP values are calculated based on the length of the lag phase, which is influenced by the sample compared to a standard. This method monitors the ability of antioxidant compounds to interfere with the reaction between ROO· generated by AAPH and a target probe (Prior et al. 2005). Various versions of the method have used oxygen uptake (Wayner et al. 1985), fluorescence of R-phycoerythrin (Ghiselli et al. 1995), or absorbance of ABTS as the reaction probe (Bartosz et al. 1998). The test measures oxygen consumption during a controlled lipid peroxidation reaction induced by the thermal decomposition of an azo-compound. It has been reported that this method was the most widely used for measuring the total antioxidant capacity of plasma or serum in the past decade (Leinonen et al. 1998).

The TRAP assay uses ROO· generated from AAPH and peroxidizable materials. After AAPH is added to the plasma, the oxidation of the peroxidizable materials is monitored by measuring the oxygen consumed during the reaction. During the induction period, this oxidation is inhibited by antioxidants in the plasma (Prior and Cao 1999). Ghiselli and colleagues proposed modifications to avoid interferences from plasma proteins, lipids, and metal ions (1995). Valkonen and Kuusi used dichlorofluorescein-diacetate as a fluorescent oxidizable substrate (1997). In both studies, they evaluated the contributions and synergistic effects of major antioxidant compounds and the impact of plasma storage on TRAP values. The oxidation of dichlorofluorescein-diacetate by ROO· forms a highly fluorescent dichlorofluorescein product. In this case, antioxidant compounds inhibit the increase in fluorescence signal competitively (Gulcin 2012).

The TRAP assay initiates lipid peroxidation by generating water-soluble ROO· and is sensitive to all known chain-breaking antioxidants. However, it is relatively complex and time-consuming to perform, requiring a high level of expertise and experience (Prior et al. 2005). This method also allows the quantification of the absorbance capacity of antioxidants specifically toward three potent oxidants: hydroxyl radicals, peroxyl radicals, and peroxynitrite (Regoli and Winston 1999). An earlier detection method used the principle that peroxyl radicals produced from AAPH oxidize luminol, leading to the formation of luminol radicals that emitted light. This emitted light was detected by a luminometer. Under normal conditions, this reaction produces low-intensity light emission that decays rapidly. However, the characteristics of the reaction can be significantly altered by adding the enhancer para-iodophenol, which produces a more intense, prolonged, and stable light emission. The light emission is sensitive to interference by antioxidants. The length of the induction period is compared to an internal standard, such as trolox, and quantitatively related to the antioxidant capacity of food constituents (Somogyi et al. 2007). One of the major problems with the original TRAP assay is the use of the oxygen electrode as a detector, as it may not maintain its stability over the required period (Rice-Evans and Miller 1994). This issue was addressed by improving the assay with β-phycoerythrin as the fluorescent probe. The ability of plasma to protect β-phycoerythrin from ROO· oxidation was monitored fluorimetrically (DeLange and Glazer 1989). Regardless of the variations discussed above, the quantification is based on the duration of the lag phase, in which oxidation is inhibited by antioxidants, compared to the lag phase of trolox. The antioxidant capacity was expressed as trolox equivalents (TE) is calculated as

$$\text{Trolox equivalents }(\text{TE})=({\text{A}}_{\text{Trolox}}/{\text{B}}_{\text{Trolox}})\text{ x }{\text{A}}_{\text{AO}}$$

where A_Trolox_ is the trolox concentration, whilst B_Trolox_ is the lag time of the kinetic curve of target oxidation in the presence of trolox. A_AO_ is the lag time of the kinetic curve of target oxidation in the presence of antioxidant molecules. Additionally, it was reported that the main drawback of the TRAP assay is the use of the lag phase for quantifying antioxidant capacity, as not every antioxidant has a clear lag phase, and the antioxidant capacity profile after the lag phase is completely overlooked (Somogyi et al. 2007). Furthermore, Mulholland and Strain (1991) reported that serum TRAP experimental values were significantly lower in patients with acute myocardial infarction compared to sex- and age-matched controls. Moreover, plasma TRAP experimental values were also found to decrease significantly by about 40% during chemotherapy in patients with various hematologic malignancies (Durken et al. 1995).

### Chemiluminescence assay

**Fig. 26:**
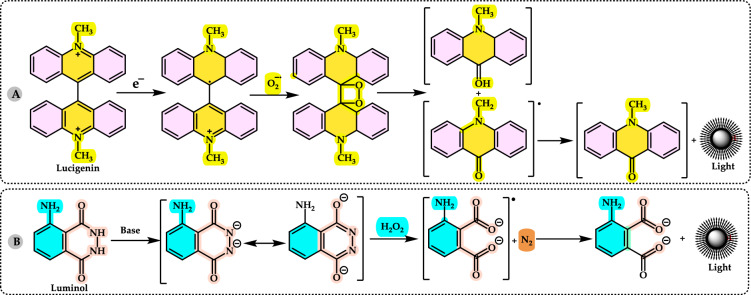
Chemiluminescent mechanism lucigenin in the assay of superoxide (**A**), and luminol **(B**)

Chemiluminescence is a phenomenon that occurs when a chemical reaction produces an electronically excited species that either emits light (directly chemiluminescence) or transfers energy to a fluorophore, which then emits light (indirect chemiluminescence). The main method for measuring antioxidant activity through chemiluminescence is direct chemiluminescence, which typically involves a chemiluminescent species, an oxidant (such as H_2_O_2_) with or without a metal or enzymatic catalyst, and an antioxidant molecule or plant extract. Various chemiluminescent reagents, including lucigenin (Fig. 26A), luminol (Fig. 26B), oxalates, Cypridina luciferin, and pyrogallol, are used in these assays. Among them, luminol is the most commonly used aqueous chemiluminescent reagent. Luminol reacts with the strong oxidizing agent H_2_O_2_ to yield 3-aminophthalate in an excited electronic state, which emits light. Antioxidant molecules can quench H_2_O_2_ by donating a hydrogen atom, inhibiting H_2_O_2_-induced chemiluminescence. In this assay, antioxidants compete with luminol for H_2_O_2_, and the presence of antioxidants results in a decrease in light emission intensity. Sometimes, a catalyst is added to enhance the chemiluminescence signal by converting the relatively weak oxidant H_2_O_2_ into stronger radicals, such as O_2_^•−^ and OH• (Shahidi and Zhong 2015).

In alkaline DMSO, luminol can be oxidized by various oxidants in the presence of O_2_, generating a luminol radical that reacts with O_2_^•−^, leading to the formation of an unstable endoperoxide. The chemiluminescence assay occurs when energy is transferred to chemicals with compatible energy states, making them emit light either directly or by a sensitized process. This assay can be used to quantify the concentration of antioxidants based on the measurement of light emission intensity. Chemiluminescence assays are based on the interaction of ROS with chemiluminescent compounds. ROS formation is detected by chemiluminescence using luminol, which is sensitive to hypochlorous acid (HOCl), while lucigenin is oxidized by superoxide. It is known that OH• or O_2_^•−^ can oxidize both luminol and lucigenin (Shivakumar and Kumar 2018). The mechanism of luminol chemiluminescence is shown in Fig. 26B. In this reaction, luminol is converted into a resonance-stabilized dianion (1) in a basic solution, which is then oxidized by H_2_O_2_ into a dicarboxylate ion (2), releasing molecular nitrogen. The dicarboxylate ion is in an excited state and emits a photon of light (hν) as it returns to its ground state (3) (White et al. 1964). Antioxidant properties of many biologically active compounds are evaluated by permanganometric chemiluminescence assays, where high luminescent O_2_^•−^ is formed from potassium superoxide (KO_2_) and 18-crown-6(1,4,7,10,13,16-hexaoxacyclooctadecane) in dimethyl sulfoxide (DMSO). Antioxidants have gained greater attention through permanganometric chemiluminescence assays (Shivakumar and Kumar 2018).

Potassium permanganate (KMnO₄) exhibits a characteristic red chemiluminescence emission at 689 nm, which results from the relaxation of Mn^2^⁺ species from an excited state (Adcock et al. 2007). A radical intermediate is generated by the oxidation of an antioxidant by Mn^4^⁺. This permanent radical species reacts with Mn^3^⁺, which is already present in the solution, to produce a Mn^2^⁺ emission source. The enhancement in chemiluminescence intensity is due to the prevention of disproportionation of the Mn^3^⁺ intermediate, where polyphosphates form a cage-like structure around Mn^2^⁺, limiting nonradiative pathways.

### Reducing antioxidant power

Reducing antioxidant power is based on the principle that the absorbance of reaction mixtures increases. An increase in absorbance indicates a rise in antioxidant activity. Reduction of a chemical is defined as the gain of electrons, while oxidation is the loss of electrons. A reductant or reducing agent is a substance that donates electrons, thereby causing another reactant to be reduced. An oxidant or oxidizing agent is a substance that accepts electrons and causes another reactant to be oxidized. Oxidation cannot occur without a corresponding reduction elsewhere in the system. When reduction and oxidation occur in a chemical reaction, it is called a redox reaction. Redox reactions are the primary reactions in biological oxidation, the chain of chemical reactions in which oxygen from the air is used to oxidize chemicals derived from food breakdown to provide energy for living systems (Gulcin 2012). While reductant and oxidant are chemical terms, antioxidant and pro-oxidant are terms used in the context of biological systems. Additionally, an antioxidant that can effectively reduce pro-oxidants may not be capable of efficiently reducing Fe^3^⁺. In this assay, the presence of reductants would result in the reduction of Fe^3+^ to Fe^2+^. The reducing capability of a bioactive compound can be calculated by means of the direct reduction of ${\text{Fe}(\text{CN})}_{6}^{3-}$ to ${\text{Fe}(\text{CN})}_{6}^{4-}$. Addition of free Fe^3+^ to the reduced product brings about the formation of the intensive Perl’s Prussian blue complex, Fe_4_[Fe(CN^−^)_6_]_3_, which has a strong absorbance at 700 nm. The Fe^3+^ reducing assay gets advantage of an electron chain reaction where a ferric salt is utilized as an oxidant.

$$\text{Reduced} {\text{antioxidant}+\text{ Fe}}^{3+}\to {\text{Oxidised antioxidant}+\text{ Fe}}^{2+}$$

$${\text{Fe}}^{2+ }+ {\text{Fe}(\text{CN})}_{6}^{3-}\to {\text{Fe}\left[{\text{Fe}(\text{CN})}_{6}\right]}^{-}$$

or

$$\text{Reduced} \text{antioxidant}+ {\text{Fe}(\text{CN})}_{6}^{3-}\to \text{Oxidized antioxidant}+ {\text{Fe}(\text{CN})}_{6}^{4-}$$

$${\text{Fe}}^{3+ }+ {\text{Fe}(\text{CN})}_{6}^{4-}\to {\text{Fe}\left[{\text{Fe}(\text{CN})}_{6}\right]}^{-}$$

An antioxidant is a reductant, but a reductant is not necessarily an antioxidant (Prior and Cao 1999). As reported in many studies, the activities of natural antioxidants in influencing diseases are closely related to their ability to reduce DNA damage, mutagenesis, carcinogenesis, and inhibition of pathogenic bacterial growth (Roginsky and Lissi 2005).

#### Ferric reducing antioxidant power (FRAP assay)

**Fig. 27:**
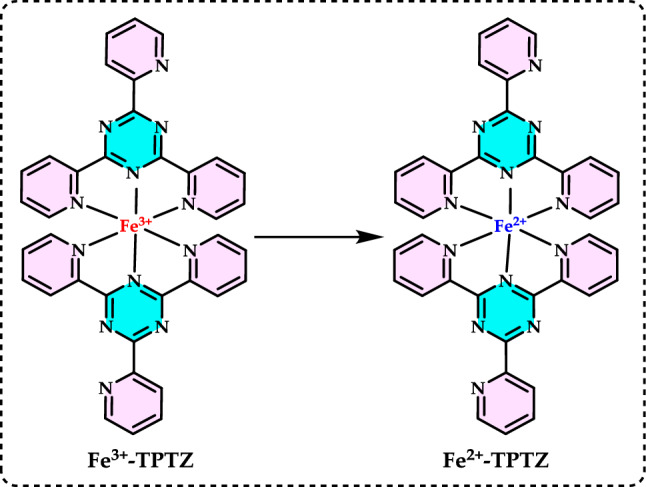
[Fe^3+^-(TPTZ)_2_]^3+^-[Fe^3+^-(TPTZ)_2_]^2+^ complex reduction reaction of FRAP assay

The FRAP assay is a typical ET-based method that measures the reduction of ferric ions (Fe^3^⁺)-ligand complex to the intensely blue-colored ferrous ions (Fe^2^⁺) complex by antioxidants in an acidic medium. In other words, this method is based on the ability of antioxidants to reduce the ferric 2,4,6-tripyridyl-s-triazine complex [Fe^3^⁺-(TPTZ)₂]^3^⁺ to the intensely blue-colored ferrous complex [Fe^2^⁺-(TPTZ)₂]^2^⁺ in an acidic medium. This reduction is monitored by measuring the increasing absorbance at 593 nm using a spectrophotometer, and the results are expressed as micromolar Fe^2^⁺ equivalents or relative to an antioxidant standard (Benzie and Strain 1999). Originally applied to plasma analysis, FRAP was later extended to other biological fluids, foods, plant extracts, beverages, teas, vegetables, juices (Gulcin 2012), spices (Gülçin et al. 2004a; 2005b), and fruits (Gülçin et al. 2005a; 2011b; 2011c; 2012c; Bursal and Gülçin 2011). As illustrated in Fig. 27, the reaction measures the reduction of ferric 2,4,6-tripyridyl-s-triazine (TPTZ) to a colored product (Gulcin 2012).

The FRAP assay is performed at an acidic pH of 3.6 to ensure the solubility of iron. Conducting the reaction at low pH reduces the ionization potential driving electron transfer and increases the redox potential, altering the dominant reaction mechanism (Hagerman et al. 1998). It has been suggested that the capacity to reduce iron is not strongly correlated with the radical-quenching processes (H-atom transfer, HAT) that most antioxidants mediate. However, the oxidation or reduction of radicals to ions still effectively terminates radical chain reactions, and reducing power provides insight into a compound's ability to influence redox balance in plasma and tissues. The FRAP assay operates purely on an ET mechanism, without involving mixed SET and HAT mechanisms. As such, it can be particularly valuable when used alongside other methods to differentiate the dominant mechanisms of action for various antioxidants (Prior et al. 2005). Furthermore, since reduced metals can actively propagate radical chains by reducing hydroperoxides to RO, it is worth investigating whether high FRAP values correlate with the potential of polyphenols to act as pro-oxidants under certain conditions. This pro-oxidant behavior has been demonstrated for certain flavones and flavanones, which also exhibit significant FRAP values (Cao et al. 1993). FRAP values are determined by measuring the increase in absorbance at 593 nm and comparing it to a ferrous ion standard solution or an antioxidant standard solution. The increase in absorbance is directly proportional to the cumulative FRAP value of the antioxidants present in the sample (Ou et al. 2002a, b). For polyphenols like caffeic acid, tannic acid, ferulic acid, ascorbic acid, and quercetin, the absorbance (λ_593_) continues to increase gradually, even hours after the reaction begins. Therefore, a single-point absorbance measurement may not accurately reflect the completion of the reaction (Prior et al. 2005). However, the FRAP assay has notable drawbacks. Since no free radicals are introduced into the system, it cannot compare antioxidant activity against different types of radicals. Moreover, it cannot accurately assess the antioxidant capacity of compounds that react with ferrous ions (Fe^2+^) or those containing SH groups (Somogyi et al. 2007). One FRAP unit is arbitrarily defined as the reduction of 1 M Fe^3+^ to Fe^2+^ (MacDonald-Wicks et al. 2006). This method also does not account for the reducing ability of thiols or carotenoids (Pulido et al. 2000; Ou et al. 2002a, b).

Another issue is the concomitant generation of Fe^2+^, a known prooxidant that can produce additional radicals, such as OH· from H_2_O_2_. Compounds that absorb light at 593 nm may also interfere with measurements, leading to an overestimation of FRAP values. For example, Benzie and Strain (1999) reported unusually high FRAP values for bilirubin because it is oxidized to biliverdin, which strongly absorbs at this wavelength. Furthermore, the acidic pH (3.6) used in the assay may cause protein precipitation, such as casein in milk samples (Chen et al. 2003a, b). Despite its low pH compared to physiological conditions (pH 7.4), the FRAP assay can indirectly reflect antioxidant capacity. However, its results should ideally be compared with those from HAT-based assays, as discrepancies may arise due to chemical variability among diverse sample types and differences in reactivity rates for various compounds in the assay.

FRAP assay has several limitations. Compounds with slower reaction rates may require extended time for accurate measurement. For instance, dietary polyphenols such as caffeic acid and quercetin react slowly, which may lead to underestimation when using short reaction times (Pulido et al. 2003). Any compound with a redox potential lower than the Fe^3^⁺/Fe^2^⁺ pair can contribute to FRAP values, potentially leading to overestimation. In this limitation, any compound with a redox potential lower than the Fe^3+^/Fe^2+^ redox pair can theoretically reduce Fe^3+^ to Fe^2+^, potentially leading to inflated FRAP values. Conversely, not all antioxidants reduce Fe^3+^ quickly enough to be measured within the observation period. The FRAP assay was developed based on the assumption that redox reactions proceed rapidly, completing within 4–6 min. However, this is not always the case, and FRAP results can vary significantly depending on the reaction time. Rapid-reacting phenols, which may bind iron or degrade into less reactive compounds, are best measured with shorter reaction times. In contrast, certain polyphenols, such as caffeic acid, ferulic acid, quercetin, and tannic acid, react more slowly and require extended reaction times for accurate quantification. Pulido et al. (2003) observed that dietary polyphenols exhibit slower reaction kinetics, with their reactivity order varying based on the reaction time. Additionally, the FRAP assay does not measure thiol-based antioxidants like glutathione and primarily evaluates reducing capacity based on the ferric ion, which is not directly linked to the mechanistic or physiological relevance of antioxidant activity. Also, FRAP does not measure thiol-containing antioxidants (e.g., glutathione) or antioxidants that interact poorly with ferric ions, such as SH-group antioxidants and carotenoids. Compounds absorbing at 593 nm may interfere with the assay, leading to inflated results. For example, bilirubin exhibits unusually high FRAP values because it oxidizes to biliverdin, which strongly absorbs at this wavelength. he low pH (3.6) required for the assay may cause protein precipitation (e.g., casein in milk samples) or other artifacts. The generation of Fe^2^⁺ during the assay may act as a prooxidant, potentially producing hydroxyl radicals (OH·) in the presence of H_2_O_2_ (Benzie and Strain 1999). Despite these drawbacks, the FRAP assay is straightforward, cost-effective, and does not require specialized equipment. Nonetheless, the FRAP assay remains simple, fast, cost-effective, and robust, with the capability to be conducted using manual, semi-automated, or fully automated methods (Prior et al. 2005).

In summary, the FRAP assay is a straightforward and affordable method that provides a general index of antioxidant capacity (Magalhaes et al. 2008). Ou et al. (2002a, b) analyzed numerous freeze-dried vegetable samples using both ORAC and FRAP methods. Their results indicated that antioxidant values depend not only on the vegetable species but also on geographical origin and harvest time. The two assays also showed differing antioxidant activity trends. They concluded that the ORAC assay is more chemically relevant for measuring chain-breaking antioxidant activity, while the FRAP assay has limitations such as potential interference, reaction kinetics, and quantification challenges. Unlike assays based on HAT, such as ORAC, FRAP does not account for chain-breaking antioxidant activity. Studies have shown that FRAP and ORAC yield different trends in antioxidant activity, depending on the species, geographical origin, and harvest time of the sample (Ou et al. 2002a, b). While FRAP provides a general index of reducing power, ORAC is more chemically relevant for evaluating chain-breaking antioxidant activity. In conclusion, while the FRAP assay is simple and economical, its results should be interpreted cautiously, particularly in comparison to other assays, due to its limitations in addressing reaction kinetics, specificity, and interferences (Magalhaes et al. 2008).

#### Cupric ions (Cu^2+^) reducing antioxidant power (CUPRAC assay)

**Fig. 28:**
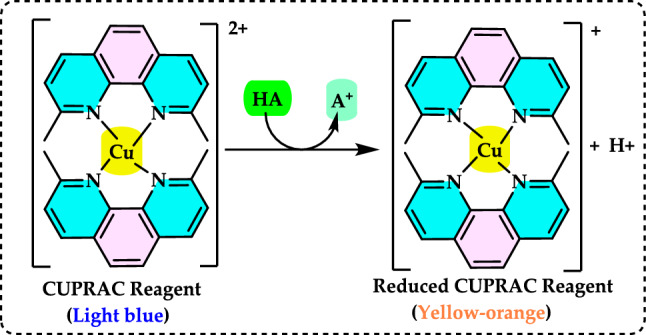
CUPRAC mechanism by an antioxidant (HA). The ammonium acetate buffer neutralizes protons liberated in the reaction

Cupric ions (Cu^2+^) reducing assay was first developed and utilized by Apak et al. (2022). It has since been applied to a variety of matrices containing both lipophilic and hydrophilic antioxidants. The principle of the assay is based on the reduction of Cu^2^⁺ to Cu⁺ by the collective action of all antioxidants or reducing agents present in an aqueous-ethanolic medium at pH 7.0. In the presence of neocuproine (2,9-dimethyl-1,10-phenanthroline), polyphenols reduce Cu^2^⁺ to form Cu⁺ complexes with a maximum absorption peak at 450 nm (Fig. 28) (Gülçin, 2008).

This method can be employed to determine the antioxidant capacity of food components using the Cu^2^⁺-neocuproine reagent as the chromogenic oxidizing agent. The reduction of Cu^2^⁺ in the presence of neocuproine by a reducing agent produces a Cu⁺ complex, which exhibits a maximum absorption peak at 450 nm (Tutem et al. 1991). The CUPRAC reagent is more stable and readily available compared to chromogenic radical reagents. It produces perfectly linear absorbance-concentration curves. In this reducing assay, any liberated protons can be buffered using a relatively concentrated acetate buffer. During the reaction, the reactive Ar–OH groups of polyphenols and other antioxidants are oxidized to their corresponding quinones, while Cu^2^⁺-neocuproine is reduced to the highly colored Cu⁺-neocuproine complex. This complex, with its characteristic absorption peak at 450 nm, can be measured spectrophotometrically against a reagent blank. Neocuproine is a heterocyclic organic compound and a chelating agent.

The CUPRAC assay, which measures the reduction of Cu^2^⁺ to Cu⁺ by antioxidants, is a cost-effective, rapid, stable, and selective method. It is suitable for evaluating a wide range of antioxidants, irrespective of their chemical nature or hydrophobicity. Furthermore, it has been suggested that the results from in vitro cupric ion (Cu^2^⁺) reducing measurements may better correlate with potential in vivo antioxidant reactions. The CUPRAC assay is conducted at a pH (7.0) that is close to physiological pH. Unlike the widely used FRAP assay, which is insensitive to antioxidants with –SH groups, the CUPRAC method can measure thiol-type antioxidants such as glutathione and non-protein thiols (Gülçin and Daştan 2007; Karaman et al. 2009).

The CUPRAC reagent is highly selective due to its lower redox potential compared to Folin or ferric ion-based oxidative reagents and is similar to the redox potential of the ABTS⁺/ABTS redox couple. Furthermore, substances like simple sugars and citric acid, which are not considered true antioxidants, are not oxidized by the CUPRAC reagent. In contrast, most phenolic antioxidants are readily oxidized owing to their favorable redox potentials. Certain antioxidants that remain unreactive with Fe^3^⁺-based reagents, such as FRAP, or Fe^3^⁺ to Fe^2^⁺ transformation methods (e.g., thiols), are effectively oxidized by the CUPRAC reagent. The assay’s optimal pH is 7.0, which is close to physiological pH (7.4), thereby simulating antioxidant activity under realistic conditions. Additionally, this method can measure both hydrophilic and lipophilic antioxidants (Apak et al. 2022).

#### Ferric ions (Fe^3+^) reducing antioxidant power

The Fe^3^⁺ reducing assay is a method used to measure the electron-donating capacity of a compound or sample, which reflects its antioxidant power. This assay evaluates the ability of bioactive molecules or antioxidants to reduce Fe^3^⁺ to Fe^2^⁺, a process indicative of their potential to neutralize oxidative agents. The reducing power of bioactive compounds or food components indicates their electron-donating ability and is closely related to their antioxidant activity. Bioactive compounds with antioxidant properties can act as reductants, neutralizing oxidants in the process (Koksal and Gulcin 2008). The reducing capacity of a bioactive compound can be evaluated by its ability to directly reduce Fe[(CN)₆]^3^⁻ to Fe[(CN)₆]^2^⁻. When free Fe^3^⁺ is added to the reduced product, it reacts to form the intense blue-colored Perl's Prussian blue complex, Fe₄[Fe(CN)₆]₃, which exhibits strong absorbance at 700 nm (Bursal and Gülçin 2011).

$${\text{Fe}}\left( {{\text{CN}}} \right)_{6}^{3 - } + {\text{AH}} \to {\text{Fe}}\left( {{\text{CN}}} \right)_{6}^{4 - } + {\text{A}}^{.} + {\text{H}}^{ + }$$

$${\text{Fe}}\left( {{\text{CN}}} \right)_{6}^{4 - } + {\text{Fe}}^{3 + } \to {\text{Fe}}_{4} [{\text{Fe}}\left( {{\text{CN}}} \right)_{6}^{4 - } ]_{3} + {\text{A}}^{.} + {\text{H}}^{ + }$$

An increase in the absorbance of the reaction mixture signifies an enhancement in reducing capacity, resulting from greater complex formation. Several assays have been developed to assess overall antioxidant activity or reducing potential as an indicator of an organism’s ability to resist free radical-induced stress. The Fe^3^⁺ reducing antioxidant power assay is based on a SET mechanism, where a ferric salt serves as the oxidant (Gulcin 2009). In this assay, the test solution’s yellow hue shifts to varying shades of green and blue, depending on the reducing strength of the antioxidant samples. The reducing capacity of a compound can be a vital indicator of its potential antioxidant activity (Gülçin et al. 2010c).

Antioxidant compounds reduce Fe^3^⁺-ferricyanide complexes to their Fe^2^⁺ form. The addition of FeCl₃ to the ferrous form results in the formation of the Prussian blue complex. Consequently, the degree of reduction can be determined by measuring the intensity of Perl’s Prussian blue at 700 nm (Gülçin 2002). Fe^2^⁺ forms a blue-colored Prussian blue complex with potassium ferricyanide, which absorbs strongly at the indicated wavelength. The color intensity, proportional to the amount of Fe^2^⁺ formed, is measured using a spectrophotometer. A standard curve (e.g., using ascorbic acid or Trolox) can be used to quantify the antioxidant capacity of the sample. In this method, the yellow solution transitions to green or blue based on the antioxidant’s reducing power, with higher absorbance reflecting greater ferric reducing potential (Gülçin et al. 2002a). However, it has been noted that results may vary depending on the analysis time, as the reaction between antioxidants and Fe^3^⁺ can take anywhere from several minutes to several hours (Pulido et al. 2000).

The advantages of the Fe^3^⁺ reducing assay are simple and cost-effective, does not require specialized equipment, provides a quick estimate of the antioxidant potential. The Fe^3^⁺ reducing assay has several limitations including does not distinguish between different types of antioxidants, sensitive to interfering substances that might also reduce Fe^3^⁺ and reaction conditions (e.g., pH, time) may influence results. The Fe^3^⁺ reducing assay remains a widely used and reliable method for assessing the reducing power and antioxidant activity of biological and chemical samples (Gulcin 2020).

### Folin–Ciocalteu reducing capacity (FCR assay)

Total Phenolic Content (TPC) is not a direct method for measuring antioxidant activity. Instead, it determines the concentration of phenolic compounds in plant and food materials, typically expressed as equivalents of gallic acid or another reference phenolic compound. Common alternatives include caffeic acid, catechin, chlorogenic acid, or ferulic acid equivalents. While TPC is not inherently an antioxidant assay, a higher phenolic content is often correlated with stronger antioxidant capacity, making it a significant parameter in evaluating total antioxidant potential. This method is widely used to analyze antioxidant-rich extracts from sources such as herbs, spices, fruits, cereals, and legumes. However, it has limitations, including sensitivity to factors like pH and temperature, which can influence the reaction time (Shahidi and Zhong 2015).

Phenolic compounds, estimated to number around 8000 in nature, share a general structure consisting of an aromatic hydroxyl nucleus (Ozturk Sarikaya et al. 2011). These compounds play a critical role as primary antioxidants and free radical scavengers. Plant polyphenols are highly versatile, functioning as reducing agents, hydrogen atom donors, and singlet oxygen quenchers. Additionally, some polyphenols exhibit metal-chelating properties, effectively inhibiting Fenton-type oxidation reactions by binding transition metal ions in their free states (Rice-Evans et al. 1996; Karaman et al. 2009; Gulcin 2012).

The Folin–Ciocalteu assay is a widely recognized method for determining total phenolic content. Initially developed to analyze proteins by targeting tyrosine, a phenolic amino acid (Folin and Ciocalteu 1927), it was later adapted to quantify phenolic compounds in food and plant extracts (Singleton et al. 1999). This method has been extensively used to measure the total phenolic content in plant materials (Gülçin et al. 2003a; Oktay et al. 2003). The Folin–Ciocalteu reducing (FCR) assay is a modified version of the Folin–Denis reducing (FDR) capacity assay, which originally aimed to measure tyrosine and tryptophan amino acids with greater sensitivity and reproducibility. Both methods are based on the reaction of FCR with an oxidizing agent, resulting in the formation of reduced molybdenum blue, which is proportional to the protein concentration. The primary distinction between the two lies in the molybdate concentration used in their preparation, with FCR using a higher amount to prevent the formation of a white precipitate, which can occur during FDR. The FCR assay works by reducing the FCR reagent with phenolic compounds under alkaline conditions (Shahidi and Zhong 2015). To prepare the FCR reagent, 0.1 g of sodium tungstate and 25 g of sodium molybdate are dissolved in 700 mL of distilled water, followed by acidification with 50 mL of concentrated HCl and 50 mL of 85% orthophosphoric acid (H₃PO₄). The mixture is boiled for 10 h, and 150 g of lithium sulfate (Li_2_SO_4_) is added to produce an intensely yellow solution known as the FCR reagent. While the exact chemical composition of the FCR reagent remains unclear, it is believed to consist of phosphomolybdic and phosphotungstic acid complexes that are reduced to form a blue-colored chromophore with a maximum absorbance at 765 nm (Shahidi and Zhong 2015). The FCR assay has long been utilized as a method for determining total phenolic content in natural products (Prior et al. 2005). This approach estimates the phenolic content in foods by relying on the electron transfer from phenolic compounds to the FCR reagent in an alkaline medium. It is a simple, widely applied technique (Singleton and Rossi 1965).

The FCR, also known as Folin’s phenol reagent, Folin–Denis reagent, or the gallic acid equivalence (GAE) method, is a mixture of phosphomolybdate and phosphotungstate used in a colorimetric assay to measure phenolic and polyphenolic antioxidants (Singleton et al. 1999). The method determines the amount of phenolic compound needed to inhibit the oxidation of the reagent. While the precise chemical nature of the FCR reagent is not fully understood, it is widely believed to contain phosphomolybdic and phosphotungstic acid complexes (Singleton and Rossi 1965). The assay operates in a highly basic environment (5–10% aqueous Na₂CO₃), where phenolics are readily oxidized, leading to the generation of superoxide radicals (O_2_·⁻). These radicals then interact with molybdate ions, resulting in the formation of molybdenum oxide (MoO_4_⁺), which exhibits strong absorbance near 750 nm. The original FCR method, developed in 1927, was derived from reagents used for tyrosine analysis (Folin and Ciocalteu 1927). In this process, the oxidation of phenols by the molybdotungstate reagent produces a colored complex with a peak of maximum absorbance at 745–750 nm. The molybdenum center in these complexes is widely regarded as the reduction site, where the molybdenum ion (Mo) accepts an electron donated by the phenolic antioxidant, facilitating the formation of the characteristic color.

**Fig. 29:**
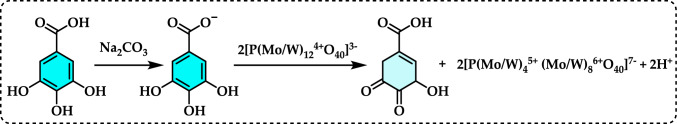
The proposed reaction involves phenolic compounds reacting with derivatives of phosphotungstic and phosphomolybdic acids in an alkaline environment, leading to the formation of a blue color, as determined by the Folin–Ciocalteu method

The Folin–Ciocalteu test is based on the reduction of the FCR by phenolic compounds in an alkaline state. While the exact chemical nature of the FCR is not clearly defined, it is thought to contain a complex of phosphomolybdic/phosphotungstic acid, which is reduced to produce a blue chromophore with a maximum absorption at 765 nm (Fig. 29). The FCR assay has long been employed to measure total phenolic content in natural products (Prior et al. 2005). This method relies on the SET in an alkaline medium, where electrons from phenolic compounds and other reducing agents are transferred to molybdenum, resulting in the formation of blue complexes detectable spectrophotometrically at wavelengths of 750–765 nm (Singleton et al. 1999). The FCR assay is recognized for its precision, sensitivity, and simplicity, making it valuable for characterizing and standardizing botanical samples, provided limitations and variables are carefully managed. However, the reaction is slow at acidic pH and lacks specificity.

An improved method introduced by Singleton and Rossi (1965) addressed variability and inconsistent results (Singleton et al. 1999). This improvement involved the use of molybdotungstophosphoric heteropolyanion reagents such as 3H₂O-P₂O₅-13WO₃-5MoO₃-10H₂O and 3H₂O-P₂O₅-14WO₃-4MoO₃-10H₂O, which are more specific for phenolic compounds, with the product exhibiting a λmax of 765 nm. Strict steps and conditions were recommended to ensure reliable data, including maintaining the correct alkali-to-FCR volume ratio, optimal reaction time and temperature, monitoring absorbance at 765 nm, and using gallic acid as a reference standard. The FCR reagent itself does not contain phenols; rather, it reacts with phenols and other reducing substances to form chromogens detectable by spectrophotometry. This reagent can also serve as a spray in chromatographic procedures. The color development occurs through electron transfer in a basic medium, reducing phosphomolybdic–phosphotungstic acid complexes and generating chromogens where the metals exist in lower valence states (Bray and Thorpe 1954).

Gallic acid is the most commonly used reference standard, with results expressed as gallic acid equivalents (GAE). Additionally, other equivalents such as pyrocatechol, tannic acid, catechin, chlorogenic acid, caffeic acid, protocatechuic acid, vanillic acid, and ferulic acid are also reported in literature (Gülçin et al. 2002b, 2003b, 2004b; Gulcin 2012). Importantly, the blue complexes formed in this assay are independent of the phenolic compound’s structure, ruling out coordination complex formation between metals and phenols (Singleton et al. 1999; Gülçin et al. 2004c; Elmastaş et al. 2006a). Despite its usefulness, the FCR assay lacks specificity for phenolic compounds. Many non-phenolic substances, including ascorbic acid, proteins, aromatic amines, sulfur dioxide, and various inorganic compounds (e.g., iron sulfate, manganese sulfate, sodium sulfite), can react with the FCR reagent, leading to inflated apparent phenolic content (Prior et al. 2005). Therefore, the assay is unsuitable for determining “total phenolic content” without accounting for or removing interfering species. It should not be confused with Folin’s reagent, which is used to detect amines and sulfur-containing compounds. Recently, the FCR assay has been proposed as a method for measuring the total reducing capacity of samples (Huang et al. 2005).

Strong correlations have been observed between FCR results and other SET-based antioxidant assays, such as TEAC and DPPH· (Roginsky and Lissi 2005; Gülçin 2005). However, it may not account for antioxidants operating via non-SET mechanisms, such as those assessed by HAT-based methods like ORAC, though correlations between these assays and FCR are generally good (Prior et al. 2005). This supports the FCR assay’s relevance for evaluating antioxidant capacity in food samples, as it is operationally straightforward, reproducible, and suitable for aqueous-phase antioxidants. Its commercial availability and absorption measurements at long wavelengths reduce interference from sample matrices, but it is unsuitable for lipophilic antioxidants. A common application of the FCR reagent is in the Lowry method for protein concentration determination (1951). Here, proteins are pre-treated with Cu^2^⁺ in a modified biuret reagent stabilized with sodium potassium tartrate. Adding the FCR generates chromogens, producing increasing absorbance between 550 and 750 nm.

### Radical scavenging assay

The free radical chain reaction is widely recognized as a primary mechanism underlying lipid peroxidation. Radical scavengers play a critical role by reacting with and neutralizing peroxide radicals, thereby halting the chain reactions of peroxidation and enhancing the quality and shelf life of food products (Soares et al. 1997). Radical chain reactions are common mechanisms in lipid autoxidation and peroxidation. Radical scavengers can interact with peroxide radicals to halt these chain reactions, thereby enhancing the stability and quality of food products. Among the mechanisms for inhibiting lipid oxidation, the radical scavenging activity of antioxidants plays the most critical role. This assay is an indispensable and standard test for evaluation of antioxidant ability studies (Gülçin and Alwasel 2023). Free radical scavenging is a key mechanism through which antioxidants inhibit lipid oxidation. This process is a standard approach in antioxidant activity research, offering a fast and efficient method to screen the radical scavenging potential of specific compounds. Among the most commonly used spectrophotometric assays to evaluate antioxidant capacity are those employing radicals such as DPPH·, ABTS^•+^, DMPD^•+^, and O_2_^·−^. These methods are highly popular for assessing the antioxidant properties of foods, beverages, and plant extracts. Both chromogenic agents and radical compounds directly interact with antioxidants. Furthermore, DPPH· and ABTS^•+^ scavenging assays are widely used because of their simplicity, speed, sensitivity, and reproducibility (Gülçin et al. 2004d). Antioxidants are thought to inhibit the free radical chain reactions of oxidation by donating hydrogen atoms from their phenolic hydroxyl groups. This donation results in the formation of stable end-products that do not propagate further lipid oxidation (Amarowicz et al. 2004).

Radical scavenging activity is of great significance due to the harmful effects of free radicals on both food stability and biological systems. Various methods are employed to evaluate the antioxidant activity of plant-derived phenolic compounds. Chemical assays typically measure the ability of antioxidants to neutralize synthetic free radicals, using diverse radical-generating systems and detection techniques to identify oxidation endpoints. Commonly applied spectrophotometric assays include DPPH•, ABTS^•+^, DMPD^•+^, and O_2_^·−^ radical scavenging methods. The addition of antioxidants to these radicals results in varying degrees of decolorization, reflecting the ability of antioxidants to counteract the formation of DPPH• radicals, ABTS^•+^, and DMPD^•+^ cations (Gulcin 2012).

$${\text{DPPH}}^{ \bullet } + {\text{AH}} \to {\text{ DPPH}}_{2} + {\text{A}}^{ \bullet }$$

$${\text{ABTS}}^{ \bullet + } + {\text{AH}} \to {\text{ ABTS}}^{ + } + {\text{A}}^{ \bullet }$$

$${\text{DMPD}}^{ \bullet + } + {\text{AH}} \to {\text{ DMPD}}^{ + } + {\text{A}}^{ \bullet }$$

These methods are quick and efficient, requiring only 15 min per sample for analysis. They are also low in labor demand, do not necessitate costly reagents, and do not rely on complex instrumentation. The chromogenic agents used in these assays are user-friendly, highly sensitive, and facilitate the rapid assessment of antioxidant activity across a large number of samples. Such assays have been widely employed to evaluate the antioxidant properties of food components (Köksal et al. 2009).

#### 1-Diphenyl-2-picrylhydrazyl (DPPH•) scavenging assay

The 1,1-diphenyl-2-picrylhydrazyl (DPPH) radical scavenging assay is one of the most commonly used methods for evaluating antioxidant activity. DPPH is a stable, long-lived nitrogen radical that appears deep blue in color. This assay is a simple, rapid and inexpensive method and widely used to evaluate the antioxidant capacity. DPPH· scavenging method depend on experiment conditions such as the reaction time and concentration, the experimental conditions (Yamauchi et al. 2024).

**Fig. 30:**
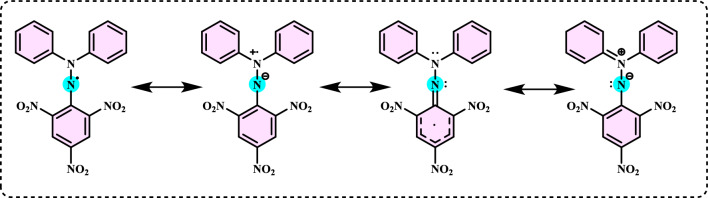
The chemical structures of 1,1-diphenyl-2-picrylhydrazil radical (DPPH∙)

The DPPH radical was first discovered by Goldschmidt and Renn in 1922. Later, Blois developed a method using this stable free radical, DPPH, to assess antioxidant activity (1958). The chemical structure of the DPPH radical (DPPH·) is illustrated in Fig. 30. This assay is based on the spectrophotometric evaluation of the ability of antioxidants to scavenge DPPH radicals. In 1995, Brand–Williams and his team further refined this test, which has since been widely adopted by researchers. Gulcin’s research group effectively utilized this method with slight modifications.

The single electron on the nitrogen atom of DPPH is reduced to form hydrazine (DPPH-H) by accepting a hydrogen atom from antioxidants. DPPH· is known for its remarkable stability and intense color, making it a popular tool in scientific research. These properties have made DPPH solutions widely used, particularly in polymer chemistry, electron paramagnetic resonance (EPR) spectroscopy, and the evaluation of antioxidant capacities of various chemicals (Bondet et al. 1997; Foti et al. 2015). The application of DPPH in assessing antioxidant capacities was first identified by Blois in 1958. The stability of the radical arises from the steric hindrance around the first-order divalent nitrogen atom and the "push–pull" effect between the second-order diphenylamino group as an electron donor and the picryl group as an electron acceptor. This effect significantly stabilizes the canonical structure. EPR spectroscopy measures the spin densities on the two hydrazyl nitrogen atoms, which are both large and essentially equal. The UV–Vis spectrum of DPPH· shows two distinct bands, primarily attributed to π − π* transitions, with the unpaired electron significantly contributing to the band in the visible region (Bondet et al. 1997; Kawai and Shibuya 2002).

When a DPPH solution is mixed with a hydrogen atom donor, such as an antioxidant, the characteristic violet color disappears, forming the reduced DPPH radical (DPPH-H). The intense violet color of DPPH· is attributed to its broad absorption band, which fades to pale yellow upon reduction. This color change is due to the transfer of hydrogen atoms from antioxidants, and the reaction is commonly referred to as the “DPPH test” in the literature. The reaction's color intensity can be easily measured using UV–Vis spectroscopy, making this method a standard for evaluating the antioxidant capacity of pure molecules, particularly plant extracts and phenolic compounds (Xie and Schaich 2014). DPPH remains a stable free radical due to the delocalization of its unpaired electron across the entire molecule (Fig. 30). This delocalization prevents the DPPH radical from dimerizing, unlike many other free radicals. Additionally, the delocalized electron is responsible for the molecule's deep purple color and its maximum absorption in ethanol at 517 nm (Erdogan et al. 2021). When the unpaired electrons are removed from the DPPH radical, the absorption decreases, and the resulting color change depends on the stoichiometry of the electron transfer. A concentrated solution of 0.5 mM in colored alcohol adheres to Lambert–Beer's law (Beer 1852). While DPPH· is only slightly soluble in nonpolar solvents, it dissolves well in various polar organic solvents and is nearly insoluble in water at room temperature. DPPH· reacts selectively with radicals and hydrogen atom donors at different reaction sites. Radicals typically target the phenyl rings, whereas hydrogen donors interact with the divalent nitrogen atom. Due to steric hindrance around the nitrogen atom, the addition of bulky radicals to this region is inhibited. However, hydrogen donors can approach the nitrogen atom and transfer hydrogen atoms, forming hydrazine (DPPH-H) (Foti et al. 2015).

Researchers have proposed using a methanolic solution of DPPH (which is readily available and easy to use) to evaluate the antioxidant activity of food and pharmaceutical components by measuring the absorbance of the remaining radicals in the reaction medium (Bondet et al. 1997). The method involves monitoring the residual DPPH radicals until equilibrium is reached. The simplicity of the process and its quick reaction time have made this approach widely popular (Blois 1958). Molecular oxygen (O_2_) does not react with DPPH· under normal conditions. However, in the presence of light, DPPH· may react slightly with molecular oxygen. DPPH· solutions stored in the dark, on the other hand, remain stable for extended periods. Unlike many free radicals, DPPH does not dimerize and exists as a free monomer in alcohol solutions (Ozcelik et al. 2003).

**Fig. 31:**
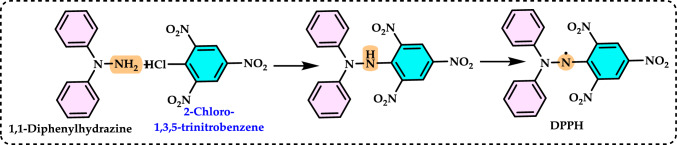
The synthesis route of 1,1-diphenyl-2-picrylhydrazil radicals (DPPH∙)

DPPH radicals can be readily prepared by oxidizing hydrazines using lead dioxide, lead tetraacetate, potassium permanganate, or silver oxide (Fig. 31). These reactions are typically performed in non-polar solvents such as benzene or dichloromethane. The desired radical can be obtained in quantitative yield through simple filtration. This method allows for the synthesis of various hydrazyl stable or persistent free radicals containing carboxyl or sulfonyl groups, often in a single step with high efficiency (Ionita 2021). It is characterized by the delocalization of its unpaired electron across the entire molecule, preventing dimerization, which is typical of freest radicals. This electron delocalization gives DPPH its deep violet hue, with a characteristic absorption peak around 517 nm in organic solvents. When mixed with an antioxidant that can donate a hydrogen atom, the DPPH radical is reduced, causing the loss of the violet color (Alam et al. 2013a, b). The DPPH radical scavenging assay is simple, cost-effective, rapid, and efficient, making it a popular method for measuring antioxidant activity and evaluating the radical scavenging capacity of non-enzymatic antioxidants (Elleuch et al. 2018). First introduced in the 1950s to identify hydrogen donors in natural substances, the DPPH assay has since been used to quantify the antioxidant potential of phenolic compounds, foods, and biologically relevant samples (Roginsky and Lissi 2005). DPPH radicals are easily obtained by oxidation of hydrazines with lead dioxide, lead tetraacetate, potassium permanganate or silver oxide (Fig. 31). These reactions are carried out in non-polar solvents such as benzene or dichloromethane. With a simple filtration, the desired radical is obtained in quantitative yield. In this way, many hydrazyl permanent or stable free radicals containing carboxyl or sulfono groups are obtained from these derivatives, generally in a single step with high yield (Blois 1958).

The DPPH radical is one of the few stable organic nitrogen radicals with a deep purple color. Unlike ABTS^·+^, which must be generated before use, DPPH is commercially available and ready for use. In the DPPH assay, the purple DPPH radical is reduced to 1,1-diphenyl-2-picrylhydrazine by hydrogen-donating antioxidants, resulting in a color change. This reduction is often monitored by measuring the decrease in absorbance or by electron spin resonance (ESR). While this method is widely used due to its simplicity, it does have some limitations, including the use of non-physiological radicals. The decolorization method, first reported by Blois in 1958, involves the scavenging of DPPH radicals by antioxidants, which reduces the absorbance at 517 nm (Blois 1958; Gülçin et al. 2009). This assay is convenient and does not require special sample treatments, but its sensitivity can be influenced by factors such as solvent type and concentration, presence of hydrogen ions or metal ions, and the freshness of the DPPH reagent (Dawidowicz et al. 2012; Shahidi and Zhong 2015).

**Fig. 32:**
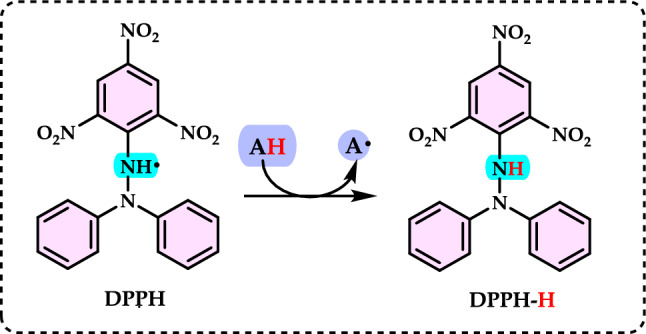
DPPH.^•^ scavenging mechanisms by an antioxidant (AH)

**Fig. 33:**
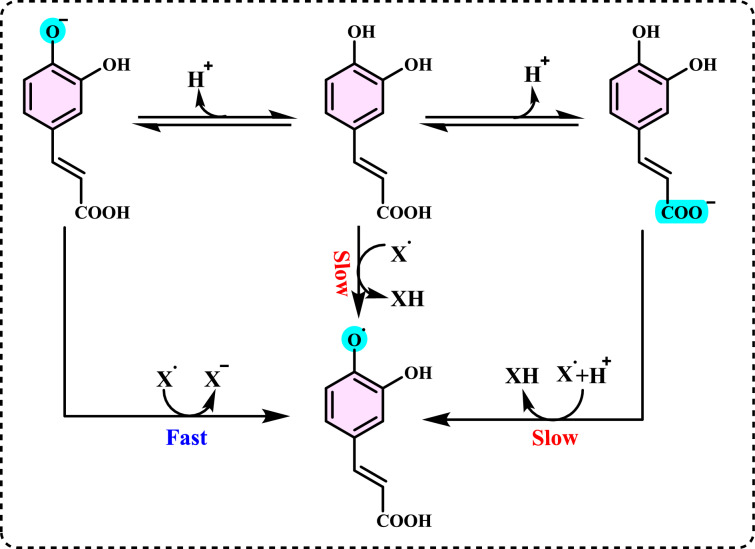
The purposed mechanism between cinnamic acids and DPPH radicals

One significant limitation of the DPPH assay is that some compounds, such as anthocyanins, absorb in the same wavelength range (500–550 nm) as DPPH, potentially causing interference and complicating result interpretation (Shahidi and Zhong 2015). In this assay, as illustrated in Figs. 32 and 33, antioxidants (AH) reduce the purple DPPH· radical to the pale yellow DPPH-H hydrazine, which can be easily measured with a UV–VIS spectrophotometer (Blois 1958; Elmastaş et al. 2006c). This simplicity and speed likely contribute to the widespread use of the assay in antioxidant screening.

The DPPH· radical is one of the few stable organic nitrogen radicals, known for its deep purple color. It is commercially available and, unlike ABTS, does not require generation prior to use. The DPPH· assay primarily relies on two mechanisms: ET and HAT. This assay measures the ability of antioxidants to reduce DPPH, reflecting their reducing power (Prior et al. 2005). As a stable free radical, DPPH· serves as a useful reagent for testing the scavenging abilities of compounds like phenols, catechols, and anilines. It is widely accepted that the interaction between phenolic compounds and DPPH can proceed via two different mechanisms: the direct HAT and the SPLET pathways (Foti et al. 2004a; Musialik and Litwinienko 2004).

$${\text{ArOH}} + {\text{DPPH}}^{ \bullet } \to {\text{ArOH}}^{ \bullet } + {\text{DPPH}}_{2 } \left( {{\text{HAT}}} \right)$$

$${\text{ArOH}} \leftrightarrows {\text{ArO}}^{ - } + {\text{H}}^{ + } \left( {{\text{SPLET}}} \right)$$

$${\text{ArO}}^{ - } + {\text{DPPH}}^{ \bullet } \to {\text{ArO}}^{ \bullet } + {\text{DPPH}}^{ - }$$

$${\text{DPPH}}^{ - } + {\text{H}}^{ + } \to {\text{ArO}}^{ \bullet } + {\text{DPPH}}_{2}$$

A freshly prepared DPPH solution is deep purple, with an absorption peak at 517 nm. This color fades when an antioxidant is present, as antioxidants can neutralize DPPH free radicals either by donating hydrogen atoms or by electron transfer. This reduces the purple color to a colorless or bleached product. In the assay, the initial absorbance of DPPH is measured, followed by the absorbance after the antioxidant has been added. The decrease in absorbance reflects the scavenging activity of the antioxidant on the DPPH radical (Gülçin et al. 2007b; Samadi et al. 2011). The blank is the reaction mixture without the test compounds. The half-maximal scavenging concentration (IC_50_) is the concentration of the sample needed to neutralize 50% of the DPPH radicals. These values are determined from the plot of inhibition percentages against concentrations (Cakmakcı et al. 2019; Oztaskin et al. 2019; Eruygur et al. 2019).

The DPPH scavenging capacity is typically evaluated in organic solvents like ethanol or methanol by tracking the absorbance decrease at 515–528 nm until it stabilizes (Brand-Williams et al. 1995; Gülçin et al. 2018; Maharramova et al. 2018). Alternatively, electron paramagnetic resonance (EPR) spectroscopy can directly measure the DPPH· concentration at submicromolecular levels, offering advantages over traditional spectrophotometry, particularly for highly colored or turbid samples (Gardner et al. 1998; Shahidi and Zhong 2015). Due to its toxicity, methanol is not preferred as a solvent. A recent study by Milardovic et al. (2006) used amperometric reduction of DPPH· at a glassy carbon electrode to determine radical scavenging capacity, where the current generated on the electrode was proportional to the residual DPPH· concentration. The reaction mechanism is primarily electron transfer, with hydrogen atom abstraction being a slower process in solvents like ethanol and methanol (Foti et al. 2004b). The solvent and pH conditions strongly influence the DPPH· scavenging activity (Magalhaes et al. 2008).

Studies have also examined the appropriate conditions for using water in a water–ethanol mixed solvent system for DPPH· assays (Stasko et al. 2007). It was found that a 50/50 aqueous/ethanol solution works well for both lipophilic and hydrophilic antioxidants, with scavenging efficiency increasing with more water in the solvent. However, above a 60/40 water–ethanol ratio, DPPH· begins to coagulate, reducing its availability for reaction. Typically, IC_50_ values are used to report antioxidant effectiveness, representing the concentration of antioxidant required to reduce the DPPH· concentration by 50% (Brand-Williams et al. 1995). The IC_50_ is a commonly used parameter for measuring radical scavenging activity, with lower IC_50_ values indicating higher efficiency. However, IC_50_ is influenced by the initial concentration of DPPH·, making absorbance changes a more reliable measure than percentage radical scavenged. The standard DPPH· concentration used in these assays is 10^–3^ M (Büyükokuroğlu et al. 2001; Büyükokuroğlu and Gülçin 2009), and results can be expressed as equivalent concentrations using a standard compound like ascorbic acid or Trolox.

The steric accessibility of the DPPH· radical is a major factor in reaction rates. Smaller molecules that can easily access the radical site tend to have higher antioxidant capacity, while larger compounds may react more slowly or remain inert. Another limitation is that DPPH· and similar radicals are not commonly found in biological systems or foods. Furthermore, spectrophotometric measurements can be influenced by compounds such as carotenoids, which absorb at similar wavelengths to DPPH, or by sample turbidity. In such cases, the electrochemical detection method proposed by Milardovic et al. (2006) may be a valid alternative. The DPPH· assay is not suitable for plasma antioxidant measurement due to protein precipitation in the alcohol medium. The reaction can take anywhere from 20 min to several hours to complete (Brand-Williams et al. 1995). Despite these limitations, the DPPH· radical is stable, commercially available, and does not need to be generated before use like ABTS^·+^. This makes it a practical and widely used method for screening the radical scavenging capacity of pure compounds (Gülçin et al. 2007c, 2008b), food constituents (Gülçin et al. 2006c, 2011c; Ak and Gülçin 2008), plant extracts (Elmastaş et al. 2006b; Serbetci and Gülçin 2010), and other samples like synthesized compounds (Talaz et al. 2009; Balaydın et al. 2010).

A recent study documented the interaction between DPPH· and usnic acid (Cetin and Gulcin 2019). The structure of usnic acid leads to interference with the DPPH· radical. After usnic acid interacts with the DPPH· radical, the DPPH· disappears, accepting either an electron or a hydrogen atom from usnic acid, resulting in the formation of DPPH_2_. This reaction between DPPH· and usnic acid is depicted in Fig. 12. Although the DPPH· scavenging mechanism of usnic acid has not been fully elucidated, it is believed that phenolic groups can stabilize the radicals formed on the phenolic carbon through their resonance structure. Usnic acid contains two hydroxyl groups on its phenolic ring, which makes it easy to donate hydrogen atoms. Usnic acid may form triradical structures by removing three DPPH radicals using its resonance structures, as shown in Fig. 12.

It has been reported that food components can exhibit strong DPPH· scavenging properties. Resveratrol, found in the skin and leaves of grapes, is a prominent constituent and belongs to the stilbene family of phenolic compounds. It is widely known for its antioxidant, cardioprotective, antidiabetic, anticancer, and antiaging effects. Recently, Gülçin (2010) demonstrated that resveratrol has significant DPPH radical scavenging activity. The antioxidant activity of polyphenolic compounds like resveratrol is attributed to the redox properties of their phenolic hydroxyl groups and the ability for electron delocalization across the molecule.

The radical scavenging reaction of phenolic compounds holds significant industrial and biological importance, as it is utilized to slow down the oxidation rate of organic matter exposed to molecular oxygen in the air. However, because peroxyl radicals react very rapidly with DPPH radicals, these reactions are challenging to monitor and require advanced equipment. In contrast, the colored DPPH· radical is readily available and exhibits much lower reactivity compared to ROO·. A prime example of this, as illustrated in Fig. 33, is the electron-transfer reaction between cinnamic acids and DPPH radicals in alcoholic solutions (Gulçin and Alwasel 2023).

**Fig. 34:**
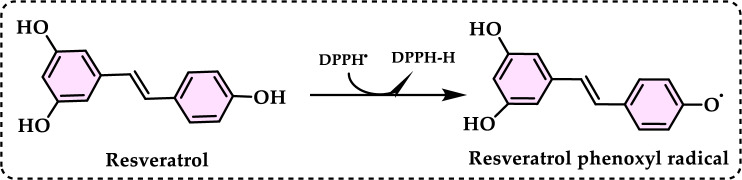
DPPH radical scavenging mechanism of resveratrol

The structure of resveratrol creates a chromophoric system, which interacts with DPPH·. The DPPH· scavenging capacity of resveratrol is summarized in Fig. 34. Like other phenolic compounds, resveratrol’s phenolic groups stabilize the radicals formed on the phenolic carbon through resonance structures.

**Fig. 35:**
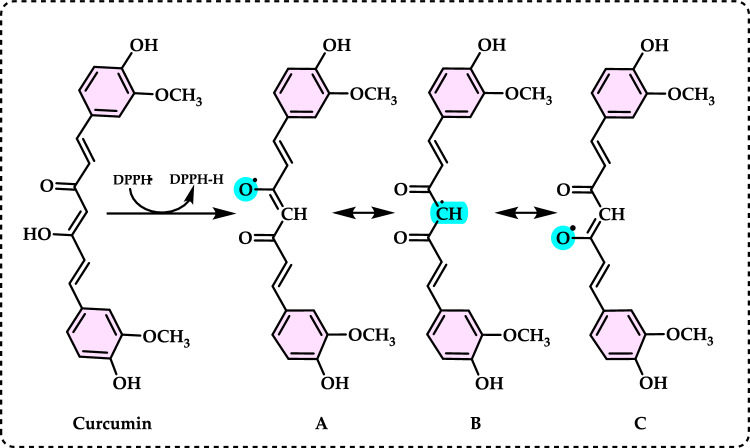
Proposed reaction between DPPH radicals and curcumin

Resveratrol contains two phenolic rings: a monophenol and a diphenol. The hydrogen atom from the monophenolic hydroxyl group can be easily abstracted (Gülçin 2010). In contrast, a different mechanism has been observed with curcumin. Curcumin is a yellow-orange hydrophobic compound extracted from *Curcuma longa*, widely used in oriental cultures. It is known for its good tolerability and safety profile and exhibits various biological properties, including antimicrobial, antioxidant, and anti-inflammatory activities (Ak and Gülçin 2008; Arshad et al. 2017). As shown in Fig. 35, in the keto form of curcumin, the heptadienone linkage between the two methoxyphenol rings contains a highly activated carbon atom. This enables curcumin to easily abstract a hydrogen atom from this carbon. However, hydrogen abstraction from the phenolic ring is more challenging since the phenolic hydrogen atoms in curcumin are intramolecularly hydrogen-bonded to the adjacent methoxy groups. Theoretical calculations suggest that intermediate B is the most stable among the intermediates (A, B, and C) (Gülçin 2012).

**Fig. 36:**
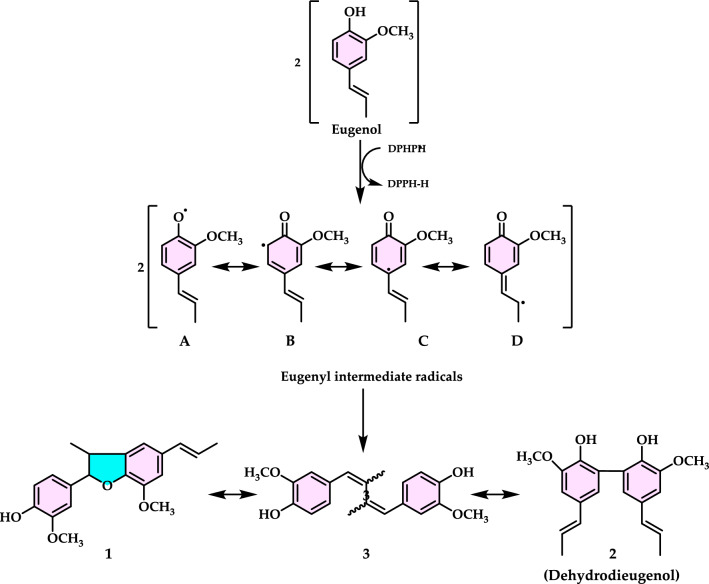
Estimated DPPH· scavenging mechanism of eugenol and formation of dehydrodieugenol by coupling reaction

The formation energy (ΔH) for compound B is calculated to be − 42.05 kcal/mol, while it is 39.45 kcal/mol for A and 54.70 kcal/mol for C. DPPH radicals are capable of easily abstracting an H-atom from the free hydroxyl group, a feature responsible for the “exceptional antioxidant” properties of curcumin. As a result, the reaction of DPPH radicals is reduced in the presence of curcumin in alcoholic media, with the phenolic portion of curcumin acting as the electron donor in this reaction (Jovanovic et al. 1999). The electron donation capacity of curcumin is evaluated by measuring its one-electron transfer to DPPH radicals. The HAT reactions of curcumin were also studied using tert-butoxyl [(CH_3_)_3_CO·] radicals, with similar results, indicating the same scavenging mechanism. Furthermore, it has been suggested that the donation of an H-atom from the β-diketone moiety of curcumin to a lipid alkyl or lipid peroxyl radical may represent an even more significant antioxidant action (Jovanovic et al. 1999; Ak and Gülçin 2008). On the other hand, Kawabata et al. (2002) reported that dimers were formed from gallic and protocatechuic acids after their reaction with DPPH radicals. Brand-Williams et al. (1995) and Bondet et al. (1997) proposed a more complex reaction mechanism involving dimeric species when studying the interaction of DPPH with BHT, eugenol, and isoeugenol. Dehydrodiisoeugenol, for instance, formed through oxidative coupling of eugenol (Bortolomeazzi et al. 2010; Gülçin 2011). To explore the role of the side chain, a group of three monophenols namely, isoeugenol, dihydroeugenol, and eugenol, differing by one double bond or its position in the chain, was also examined. Conjugation was found to enhance the antioxidant and radical scavenging activity of eugenol (Nenadis et al. 2003). The scavenging mechanism of this reaction is thought to involve the abstraction of a hydrogen atom from a phenol donor, resulting in the formation of DPPH-H and a phenoxy radical (Mastelic et al. 2008). Eugenol has been reported to reduce two or more DPPH radicals, despite the availability of hydrogen from its hydroxyl group. Different hypotheses have been proposed to explain the varying antiradical efficiencies of different monophenolic compounds (Brand-Williams et al. 1995; Mastelic et al. 2008). In another study, the co-antioxidant behavior of phenols such as eugenol and isoeugenol was investigated. A synergistic effect, where α-tocopherol is regenerated by the co-antioxidant, was observed when combining α-tocopherol with eugenol in the presence of peroxyl radicals (Kadoma et al. 2006; Gülçin 2011). As depicted in Fig. 36, the dimers formed from two phenolic hydroxyl groups in compounds A, B, and C were present in very low amounts compared to C. All of these compounds likely originated from C8–C8 and C5–C5 coupling processes (Bortolomeazzi et al. 2010).

Eugenol contains an aromatic ring, and the phenolic group in its structure stabilizes a radical formed on the carbon through conjugation. Eugenol scavenges radicals on this aromatic ring. Similarly, the structures of other aromatic compounds also feature a chromophoric system that interferes with DPPH·. It is widely recognized that phenolic groups stabilize a radical formed on the phenolic carbon through their resonance structure (Gülçin 2011).

Various methods for the DPPH assay have been described, including the use of different initial concentrations of the DPPH solution. Additionally, varying volumes of the extracts and DPPH solutions have been reported, resulting in differing final concentrations of the plant extract or pure compound and DPPH. It has also been noted that different time intervals are employed for the radical scavenging process to mature (Scherer et al. 2009). The results of DPPH assays are commonly presented in different formats, such as the percentage inhibition of the DPPH free radical (A%), calculated using the formula:

$${\text{A}}\% \, = \, \left[ {\left( {{\text{A}}_{{\text{o}}} - {\text{ A}}_{{\text{s}}} } \right)/{\text{A}}0} \right] \, \times { 1}00$$

where A_o_ represents the absorbance of the control, and A_s_ represents the absorbance in the presence of the test compound. However, most studies express the results as the IC_50_ value, which is defined as the concentration of antioxidant required to reduce the initial DPPH concentration by 50%. This is typically determined using a graph plotting the percentage inhibition against the extract concentration (Ani et al. 2006). Despite the widespread use of the DPPH method, the lack of standardized reporting complicates the comparison of antioxidant strength across different plant extracts or pure compounds. To date, no study in the literature has proposed a universal index for the DPPH assay. The data for plant extracts or pure compounds, such as inhibition percentage (I%) or the IC_50_ value, vary depending on the final concentration of DPPH used. Therefore, this study aimed to introduce a new antioxidant activity index (AAI) using the DPPH method (Scherer et al. 2009). The IC_50_ value (the concentration required for 50% inhibition) was determined graphically by plotting the extract concentration against the corresponding scavenging effect within the linear range. The intensity of IC_50_ of antioxidant activity may vary in different antioxidant as categorized in Table 9 (Fatmawaty et al. 2019).

**Table 9:** Category of antioxidant activity strength in vitro against DPPH radicals

Intensity of IC_50_	Value (µg/mL)
Very active	< 50
Active	50–100
Medium	100–250
Weak	250–500
Inactive	> 500

The antioxidant activity was expressed as the antioxidant activity index (AAI), calculated using the following formula:

AAI = Final concentration of DPPH (μg/mL)/IC_50_ (μg/mL)

This approach allows the AAI to be calculated based on the mass of DPPH and the mass of the tested compound in the reaction, producing a constant value for each compound that is independent of the DPPH and sample concentrations used. In this study, plant extracts were classified as having poor antioxidant activity when AAI < 0.5, moderate activity when AAI is between 0.5 and 1.0, strong activity when AAI is between 1.0 and 2.0, and very strong activity when AAI > 2.0. All assays were conducted in triplicate, and all sample and standard solutions, as well as DPPH solutions, were freshly prepared daily (Scherer et al. 2009).

#### ABTS^•+^ scavenging assay

The 2,2′-azino-bis(3-ethylbenzothiazoline-6-sulfonic acid radicals (ABTS^•+^) scavenging assay is a spectrophotometric method used to evaluate the antioxidant capacity of substances. It is based on the ability of antioxidants to reduce the blue–green ABTS radical cation (ABTS^•+^), which is formed by the oxidation of ABTS. In this method, ABTS is oxidized by oxidants to produce its radical cation, ABTS^•+^, which exhibits an intense color. The antioxidant capacity is determined by the ability of test compounds to reduce this color, reacting directly with the ABTS radical. ABTS^•+^ is suitable for evaluating both hydrophilic and lipophilic compounds. Like DPPH radicals ABTS^•+^ is a stable radical not found in the human body. Also, unlike DPPH radicals, which primarily undergo HAT reactions, ABTS^•+^ participate in both HAT and SET mechanisms. The generation of the ABTS radical cation forms the foundation of a widely used spectrophotometric technique to assess the total antioxidant activity of pure substances, aqueous mixtures, and beverages (Gulcin 2008). A more convenient approach involves a decolorization method where the ABTS^•+^ radical is first generated in a stable form before being exposed to potential antioxidants. ABTS^•+^ exhibits absorption maxima in aqueous media at 414, 734, and 815 nm, and in ethanolic media at 414, 730, and 873 nm.

**Fig. 37:**
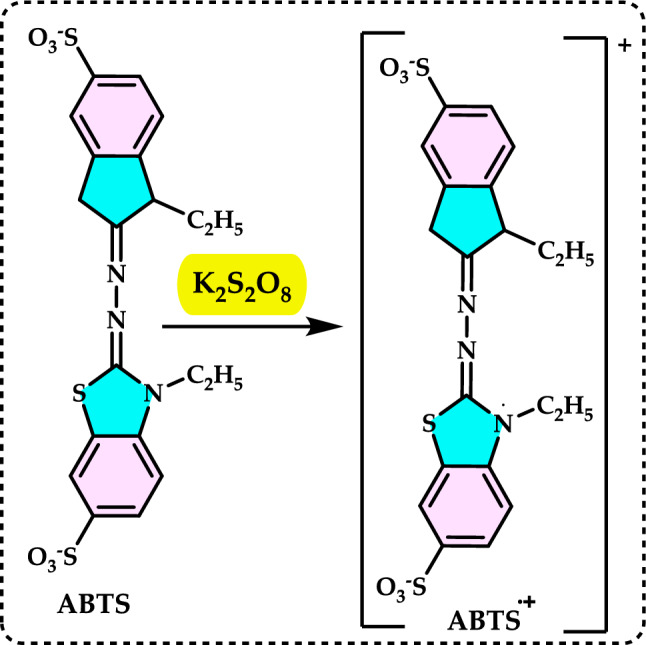
Oxidation of ABTS with K_2_S_2_O_8_ and formation of ABTS^•+^

The original ABTS^•+^ scavenging assay, developed by Miller et al. (1993), relied on metmyoglobin activated with H_2_O_2_ to generate ferrylmyoglobin radicals, which then reacted with ABTS to produce ABTS^•+^. Over time, various methods for generating ABTS^•+^ have been introduced, differing in reaction strategies, applied reaction times, detection wavelengths, and reference antioxidants. ABTS^•+^ can be generated chemically using agents like MnO_2_, AAPH (Van den Berg et al. 1999), or potassium persulfate (K_2_S_2_O_8_) (Miller et al. 1996). Alternatively, enzymatic methods utilizing metmyoglobin (Miller et al. 1993) or horseradish peroxidase (Cano et al. 1998) can be employed, as well as electrochemical generation techniques (Alonso et al. 2002). For instance, as shown in Fig. 37, chemical generation using potassium persulfate is common, involving electron transfer that splits the K_2_S_2_O_8_ molecule. Detection at 734 nm is often preferred as it minimizes interference from sample turbidity and other absorbing components (Arnao 2000). While chemical generation methods, such as the use of K_2_S_2_O_8_, often require long reaction times (up to 16 h) or elevated temperatures (60 °C for ABAP generation), enzymatic approaches offer faster and milder reaction conditions. Horseradish peroxidase has been utilized to produce ABTS^•+^ under a wide range of pH levels, with acidic conditions favoring electron transfer (Cano et al. 1998). This method has also been adapted to evaluate hydrophilic and lipophilic antioxidants by modifying reaction media or using solvent partitioning techniques (Wu et al. 2004). However, reactions in aqueous environments are generally preferred (Pulido et al. 2003). The ABTS^•+^ assay is straightforward and widely applied for antioxidant screening and routine determinations. Typically, ABTS^•+^ is generated by oxidizing ABTS with potassium persulfate, and the assay monitors the inhibition of ABTS^•+^ absorbance, particularly at 734 nm, as a measure of antioxidant activity (Gulcin 2012).

It is important to highlight that the reaction with ABTS^•+^ is remarkably rapid, typically completing within 0.25 to 0.5 min. The decolorization of a preformed blue-green ABTS^•+^ solution has been widely employed to assess the antioxidant capacity of both complex mixtures and individual compounds. The interaction between the preformed radical and free radical scavengers is easily monitored by observing the decrease in absorbance at 734 nm (Gülçin et al. 2009). The ABTS radical cation can be generated using various oxidants, with K_2_S_2_O_8_ being a commonly used agent. Studies indicate that the presence of peroxodisulfate enhances the rate of ABTS^•+^ generation. In the ABTS/K_2_S_2_O_8_ system, ABTS^•+^ is efficiently formed, making this approach a standard for evaluating antioxidant activity.

$${\text{S}}_{2} {\text{O}}_{8}^{2 - } + {\text{ABTS}} \to {\text{ SO}}_{4}^{2 - } + {\text{ SO}}_{4}^{ \bullet - } + {\text{ ABTS}}^{ \bullet + }$$

where the scission of the K_2_S_2_O_8_ could take place after the electron transfer. In the presence of excess ABTS, the sulphate radical will react according to

$${\text{SO}}_{4}^{ \bullet - } + 2{\text{ABTS}} \to {\text{ SO}}_{4}^{2 - } + { }2{\text{ABTS}}^{ \bullet + }$$

leading to the overall reaction represented by

$${\text{S}}_{2} {\text{O}}_{8}^{2 - } + 3{\text{ABTS}} \to { }2{\text{SO}}_{4}^{2 - } + { }3{\text{ABTS}}^{ \bullet + }$$

ABTS^•+^ radicals are more reactive than DPPH radicals. Unlike the reactions involving DPPH radicals, which primarily follow the hydrogen atom transfer (HAT) mechanism, those involving ABTS^•+^ radicals predominantly proceed via the single electron transfer (SET) mechanism (Kaviarasan et al. 2007; Köksal et al. 2017). The ABTS radical scavenging assay has the advantage of being applicable across a wide pH range, making it valuable for studying the influence of pH on antioxidant mechanisms in food components (Kınalıoğlu Gören and Gülçin 2024; Dikici et al. 2024). Additionally, ABTS radicals are soluble in both water and organic solvents, allowing the assessment of antioxidant capacity in both hydrophilic and lipophilic substances. However, the results are influenced by the antioxidant standard used, as it may exhibit different kinetic behaviors. This assay’s outcome is also time-dependent, with reaction times ranging from 1 to 30 min commonly reported in the literature. Despite its utility, the ABTS radical assay has faced criticism for not being representative of biomolecules or naturally occurring radicals in biological or food systems. Thermodynamically, any compound with a redox potential lower than that of ABTS^•+^ can react with the radical, which may limit its specificity (Magalhaes et al. 2008; Durmaz et al. 2024).

The advantages of this methods are suitable for testing a wide variety of compounds, including pure substances, mixtures, and biological fluids. Another advantage allows for the quantification of antioxidant capacity in diverse media and provides insights into the antioxidant activity of substances in both hydrophilic and lipophilic environments (Güven et al. 2003d; Çelik et al. 2024). However, it has some limitations. The ABTS radical is not naturally found in biological or food systems, which may reduce its biological relevance. Results can vary based on the antioxidant standard used and the assay's time of analysis. Also, ABTS^•+^ are not free radicals. They must be produced from the ABTS molecules under laboratory conditions in the presence of an oxidizing agent (Karageçili et al. 2024).

In conclusion, ABTS^•+^ reacts swiftly with antioxidants in food components, usually completing within 30 min. Its broad pH applicability allows for the study of pH effects on antioxidant mechanisms. Furthermore, ABTS^•+^ solubility in both aqueous and organic solvents, along with its independence from ionic strength, makes it suitable for determining the antioxidant capacities of both hydrophilic and lipophilic compounds in diverse media, including extracts and biological fluids (Awika et al. 2003).

#### DMPD^•+^ scavenging assay

The DMPD radical cation is an unstable species generated in situ through oxidation with potassium persulfate (K_2_S_2_O_8_) and ferric chloride (FeCl_3_). In the presence of ferric ions (Fe^3^⁺), N,N-dimethyl-p-phenylenediamine dihydrochloride (DMPD) is oxidized to form the colored DMPD radical cation (DMPD^•+^). The DMPD^•+^ assay is based on the principle that antioxidant molecules in the test samples readily scavenge DMPD radicals. This method is quite similar to the ABTS^•+^ assay, with the DMPD^•+^ assay serving as an alternative. Fogliano et al. (1999) introduced a modified version of the ABTS test, replacing ABTS^•+^ with the stable DMPD^•+^ radical cation derived from N,N-dimethylphenylenediamine. As reported by Fogliano et al. (1999) and Schleisier et al. (2002), the DMPD^•+^ assay is simpler, more efficient, and more cost-effective than the traditional ABTS assay.

**Fig. 38:**
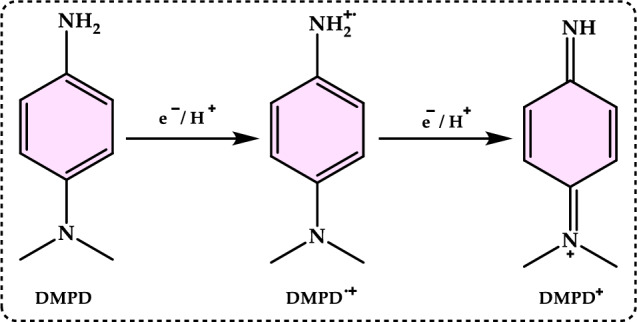
The formation of DMPD radicals (DMPD.^•+^)

In the presence of an oxidizing agent or acidic pH, DMPD is converted into a stable, colored DMPD radical cation (DMPD^•+^). Antioxidant molecules capable of donating a hydrogen atom or electron to DMPD^•+^ cause rapid decolorization of the solution. This reduction in absorbance at 505 nm is measured, with a stable endpoint indicating antioxidant activity. However, data on the stoichiometry with antioxidant standards and the stability of the radical remain limited. Another limitation is that DMPD is soluble only in water, making it unsuitable for hydrophobic antioxidants (Fogliano et al. 1999). As illustrated in Fig. 38, antioxidant compounds that transfer a hydrogen atom to DMPD^•+^ neutralize the radical, resulting in the solution's decolorization. This reaction is quick, with a stable endpoint, which serves as a measure of antioxidant efficiency. Thus, the assay evaluates the capacity of hydrogen-donating antioxidants to scavenge the single electron from DMPD^•+^. The stable endpoint reflects the antioxidant’s efficiency (Fogliano et al. 1999; Ak and Gulcin 2008).

Preliminary experiments reveal that the choice of oxidant solution and the ratio between the concentration of DMPD^•+^ and the oxidative compound are critical for the method’s effectiveness. The formation of the radical cation is notably slow, leading to a continuous increase in absorbance. The best results were achieved using FeCl_3_, which produces a stable, colored solution at a final concentration of up to 0.1 mM. Additionally, this method offers a low-cost solution and highly reproducible results (Gülçin 2008). The DMPD assay is particularly well-suited for assessing hydrophilic antioxidants but is less responsive to hydrophobic bioactive compounds, a limitation not shared by other assays. Unlike the ABTS procedure, the DMPD^•+^ scavenging method provides a very stable endpoint, which is especially beneficial for large-scale screening applications. However, a significant drawback of the DMPD^•+^ method is its reduced sensitivity and reproducibility when hydrophobic antioxidants like α-tocopherol or BHT are analyzed. Furthermore, it has been reported that organic acids can cause interference in the assay (Sanchez-Mareno 2002). Consequently, standard antioxidant compounds were not used in this antiradical assay.

#### Superoxide anion radical scavenging assay

Although the Superoxide anions radical (O_2_^• −^) is weak oxidant, it eventually generates highly reactive and harmful hydroxyl radicals and singlet oxygen, both of which play a significant role in causing oxidative stress. Superoxide anions (O_2_^• −^), although weak oxidants, ultimately generate highly reactive and harmful species such as hydroxyl radicals (OH•) and singlet oxygen (^1^O^2^), both of which contribute significantly to oxidative stress (Gulcin et al. 2006a). These radicals are biologically toxic and are utilized by the immune system to eliminate invading microorganisms. In phagocytes, superoxide anions are produced in substantial quantities by the enzyme NADPH oxidase as part of oxygen-dependent pathogen-killing mechanisms. O_2_^• −^ is an oxygen-centered radical with selective reactivity, formed by the transfer of a single electron to oxygen in vivo. It originates from various metabolic processes or is generated following oxygen activation by irradiation (Halliwell 2006). Superoxide anion is converted into H_2_O_2_ through enzymatic and nonenzymatic dismutation processes (Fridovich 1989). Subsequently, H_2_O_2_ is further transformed into the highly reactive OH• via the Fenton reaction, which requires the presence of reduced iron or copper ions (Halliwell and Gutteridge 1990). Although superoxide anions are relatively weak oxidants with limited chemical reactivity, they can generate more hazardous species, such as ^1^O_2_ and OH•, which are known to cause lipid peroxidation (Halliwell and Chirico 1993). As precursors to highly reactive free radicals, superoxide anions can interact with biological macromolecules, leading to tissue damage (Halliwell and Gutteridge 1984). Superoxide is also easily produced during the radiolysis of water in the presence of oxygen, which allows for precise measurement of reaction rate constants (Gülçin and Daştan 2007).

**Fig. 39:**
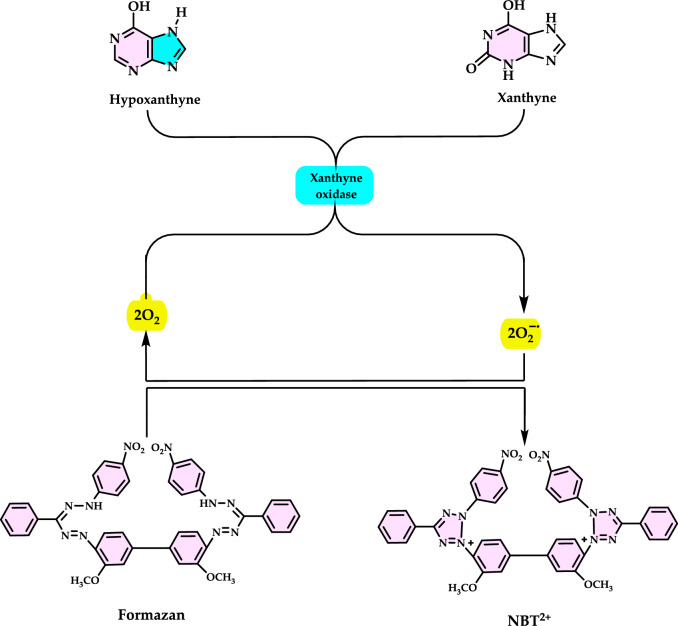
The formation of superoxide anion radicals (O_2_^•−^) in xanthine-xanthine oxidase and reducing effect of nitroblue NBT^2+^ into formazan

Superoxide anions have been implicated in several pathological processes due to their ability to transform into more reactive species like OH•. They are also known to directly initiate lipid peroxidation (Wickens 2001). Studies have shown that the antioxidant properties of certain flavonoids are effective primarily because they scavenge superoxide anions. As precursors of H_2_O_2_, OH•, and ^1^O_2_, superoxide anions induce oxidative damage in lipids, proteins, and DNA. The effects of O_2_^• −^ are magnified because they lead to the formation of other free radicals and oxidizing agents (Pietta 2000). However, detecting and measuring O_2_^• −^ levels within cells is challenging due to the instability of this reactive oxygen species (ROS) in aqueous solutions. Detection methodologies typically involve reacting superoxide with an indicator that forms a stable product through oxidation, reduction, or binding. The efficiency, sensitivity, and specificity of O_2_^• −^ detection vary significantly depending on the reaction pathways used (Fridovich 1997). Bioanalytical methods to determine O_2_^• −^ scavenging activity often use the xanthine-xanthine oxidase system at pH 7.4 to generate superoxide anion radicals. As illustrated in Fig. 39, O_2_^• −^ can reduce nitroblue tetrazolium (NBT) to formazan, which can be monitored spectrophotometrically at 560 nm in this system (Aruoma et al. 1993).

**Fig. 40:**
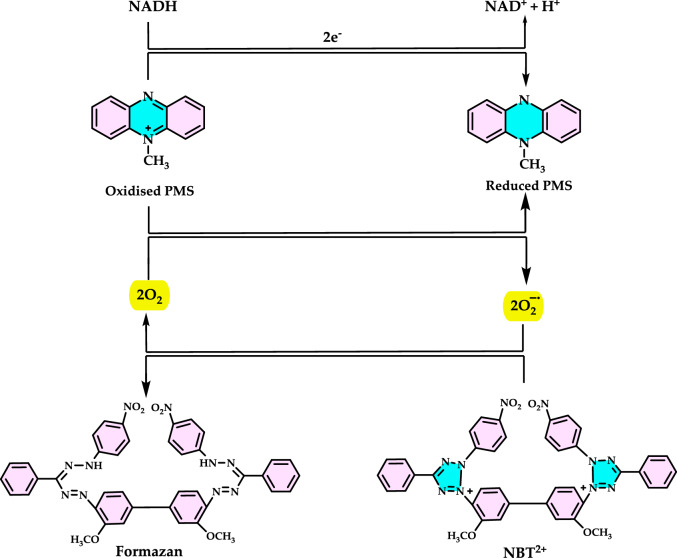
The formation of superoxide anion radicals (O_2_^•−^) in NADH-PMS and reducing effect of nitroblue NBT^2+^ into formazan

In normal tissues, xanthine oxidase functions as a dehydrogenase enzyme that transfers electrons to nicotinamide adenine dinucleotide (NAD). Under stress conditions, this enzyme converts to an oxidase, leading to the production of O_2_^• −^ and H_2_O_2_. In vitro, xanthine oxidase combined with hypoxanthine (or xanthine) at pH 7.4 can be used to generate O_2_^• −^. Xanthine oxidase catalyzes the oxidation of its substrates while reducing oxygen, thereby producing O_2_^• −^ and H_2_O_2_. Subsequently, O_2_^•−^ reduces nitroblue tetrazolium (NBT) to formazan, which can be quantified spectrophotometrically at 560 nm (Bull et al. 1983). To enhance the throughput and simplify the procedure, the assay has been adapted to a microplate format, replacing NBT with cytochrome c, and the absorbance is measured at 550 nm (MacDonald-Wicks et al. 2006). Additionally, the O_2_^• −^ scavenging capacity in the xanthine-xanthine oxidase system has been assessed by its reaction with α-ketomethiolbutyric acid, producing ethylene, which is analyzed using gas chromatography (Lavelli et al. 1999). The scavenging activity for this radical can also be determined using electron spin resonance (ESR) spectroscopy (Calliste et al. 2001). In a similar approach to the xanthine–xanthine oxidase system, O_2_^• −^ can also be generated via a non-enzymatic reaction using phenazine methosulfate (PMS) in the presence of nicotinamide adenine dinucleotide (NADH). In both systems, O_2_^• −^ reduces NBT into formazan, and the reaction is monitored spectrophotometrically at 560 nm (Aruoma et al. 1993). This method is depicted in Fig. 40.

Another widely used assay for O_2_^•−^ production is the riboflavin-methionine-illumination system. In this method, superoxide anions generated from dissolved oxygen through the riboflavin-methionine-illumination reaction reduce nitroblue tetrazolium (NBT). Specifically, the superoxide anion reacts with the yellow dye (NBT^2+^), converting it into blue formazan, which can be measured spectrophotometrically at 560 nm. Antioxidants prevent the formation of blue NBT, and a decrease in absorbance at 560 nm in the presence of antioxidants reflects the consumption of superoxide anions in the reaction mixture. This demonstrates the antioxidant’s ability to inhibit the formation of blue formazan (Parejo et al. 2002). The two principal reactions are involved in this assay are (Liochev and Fridovich 1995):

$$2{\text{NBTH}}^{ \bullet } { } \to {\text{NBT}} + {\text{NBTH}}_{2} \left( {\text{a}} \right)$$

$${\text{NBTH}}^{ \bullet } + {\text{ O}}_{2} { } \leftrightarrow {\text{NBT}} + {\text{ O}}_{2}^{ \bullet - } { }\left( {\text{b}} \right)$$

When riboflavin undergoes photochemical activation, it reacts with NBT to produce NBTH∙ (Beauchamp and Fridovich 1971; Gülçin et al. 2002a), which subsequently leads to the formation of formazan as described in reaction (a). Under aerobic conditions, radical species are controlled by a quasi-equilibrium process (b), leading to the indirect generation of superoxide anion radicals. In the presence of an antioxidant molecule capable of donating an electron to NBT, the characteristic purple color of formazan diminishes, and this decrease can be monitored spectrophotometrically at 560 nm (Gülçin et al. 2003c; 2005b). Antioxidants effectively inhibit NBT formation and scavenge superoxide anion radicals. The reduction in absorbance at 560 nm in the presence of antioxidants indicates the neutralization of superoxide anions in the reaction mixture (Bursal and Gülçin 2011).

Antioxidant molecules compete with NBT for O_2_^•−^, thereby reducing the rate of the reaction. Another commonly used probe for detecting O_2_^•−^ is cytochrome c. The reduction of ferricytochrome c to ferrocytochrome c can be kinetically analyzed and monitored at 550 nm (Aruoma et al. 1993; Quick et al. 2000; Gülçin et al. 2004d). It has been observed that the inhibition of NBT reduction is typically more pronounced than the inhibition of cytochrome c reduction (Aruoma et al. 1993). This occurs because O_2_^•−^ reacts more rapidly with cytochrome c than with NBT, causing a given concentration of O_2_^•−^ scavenger to compete less efficiently in the cytochrome c system and thereby exert a lower degree of inhibition.

**Fig. 41:**
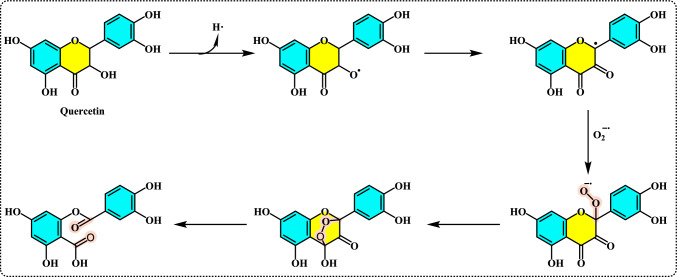
The purposed scavenging mechanism of superoxide anion radical of quercetin

Quercetin is a flavonol known for its ability to protect DNA from oxidative damage caused by OH·, H_2_O_2_ and O_2_^•−^ targeting DNA oligonucleotides. Mechanism of O_2_^•−^ scavenging activity of quercetin as a flavonoid commonly found in fruits, vegetables, and plants was shown in Fig. 41. However, quercetin has also been reported to act as a carcinogenic agent. Studies indicate that quercetin exhibits dual effects on DNA damage induced by cupric ions, depending on their concentration. Consequently, it is crucial to consider the concentration of chelating metal ions, such as copper or iron, when evaluating the protective or harmful effects of quercetin and other bioflavonoids (Nimse and Pal 2015).

The hydroxyl groups on quercetin’s aromatic rings especially at positions 3, 5, 7, and 4’ play a critical role in scavenging free radicals. These groups donate electrons to neutralize O₂·⁻. Then the quercetin radical intermediate is stabilized through delocalization of electrons across the flavonoid structure, preventing further propagation of oxidative damage. Also, quercetin radicals formed during the scavenging process can be regenerated back to quercetin through interactions with other antioxidants (e.g., vitamin C or glutathione), enhancing its antioxidant efficiency.

#### Nitric oxide radical (NO·) scavenging assays

Nitric oxide radicals (NO·) are produced in biological systems by specific nitric oxide synthases, which catalyze the conversion of arginine to citrulline with the formation of NO· through a five-electron oxidative reaction. This radical plays a critical role in regulating various physiological and pathophysiological processes (Pacher et al. 2007; Gülçin, 2012). Nitric oxide serves as an essential cell-signaling molecule in mammals, including humans (Hou et al. 1999). At low levels, NO production is significant for protecting organs like the liver from ischemic damage. Within metabolic processes, NO is synthesized endogenously from l-arginine, oxygen, and NADPH by various nitric oxide synthase enzymes. Highly reactive, NO has a short half-life of just a few seconds and diffuses easily across cellular membranes, making it an ideal transient paracrine and autocrine signaling molecule (Stryer 1995). Vriesman et al. (1997) developed a straightforward method to quantify the NO· scavenging capacity of sulfur-containing compounds in aqueous solutions using an amperometric NO· sensor. In this method, the natural logarithm of the NO· concentration and time exhibits a linear relationship. After accounting for the spontaneous degradation of NO·, the second-order rate kinetics of the scavenging reaction was determined. Their study found that only compounds containing a thiol group demonstrated significant NO· scavenging capacity. This method involves a non-competitive reaction mechanism, as the reactive species in the reaction medium are exclusively NO· and the scavenger molecules.

Additionally, the NO· scavenging capacity has been evaluated using ESR spectrometry (Asanuma et al. 2001). In this approach, NO· is generated from the donor compound 3-(2-hydroxy-1-methyl-ethyl-2-nitrosohydrazino)-N-methyl-1-propanamine, which is oxidized to NO2 by the target compound 2-(4-carboxyphenyl)-4,4,5,5-tetramethylimidazoline-1-oxyl-3-oxide (carboxy-PTIO). This reaction results in the formation of a carboxy-PTI spin adduct, which is measured via ESR. This method has been applied to assess the effects of non-steroidal anti-inflammatory drugs. However, a notable limitation of this technique is its accessibility, as the required detection technology is not widely available, and the reaction time is relatively long, approximately two hours.

Sodium nitroprusside is known to decompose in aqueous solutions at physiological pH, generating NO·. Under aerobic conditions, NO· reacts with oxygen to form stable products such as nitrate and nitrite, whose concentrations can be measured using the Griess reagent (Marcocci et al. 1994). When sulfanilamide is added, the nitrite ion reacts to produce a diazonium salt. This salt subsequently reacts with an azo dye agent, N-α-naphthyl-ethylenediamine, resulting in the development of a pink color. The diamine is used in place of the simpler and cheaper α-naphthylamine because the latter is a potent carcinogen. Additionally, the diamine forms a more polar and soluble dye in an acidic aqueous medium.

The Griess reaction is widely employed to assess NO· production by whole cells or enzymes (Krol et al. 1995). In summary, it is a two-step diazotization reaction where a nitrosating agent derived from NO (e.g., N₂O₃), formed through the acid-catalyzed conversion of nitrite to nitrous acid, reacts with sulfanilic acid to form a diazonium ion. This ion then couples with N-(1-naphthyl)ethylenediamine, producing a chromophoric azo product that strongly absorbs light at 548 nm (Grisham et al. 1996). This method is also frequently applied to evaluate NO· scavenging capacity in vitro. In such cases, the remaining amount of nitric oxide after its reaction with the test sample is quantified as nitrite. It is important to note that nitrate may also form in the reaction, necessitating its reduction to nitrite before measurement. For this purpose, NADH-dependent nitrate reductase is used, and interference from NADH is eliminated by adding lactate dehydrogenase and pyruvate (Perez et al. 2007). The resulting chromophoric azo derivative formed from nitrite during the Griess reaction is then measured spectrophotometrically at 548 nm. Standard curves are created using sodium nitrite, and results are expressed as a percentage change compared to the control response (Magalhães et al. 2008).

**Fig. 42:**
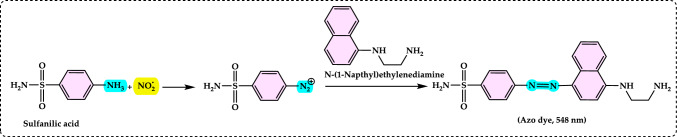
Evaluation of NO· Production Using the Griess Reagent

In this method, nitrite is detected and analyzed through the formation of a red-pink color upon treating a nitrite-containing sample with the Griess reagent. When sulfanilic acid is added, the nitrite ions form a diazonium salt. Upon the addition of the azo dye agent (α-naphthylamine), a pink color develops. A typical commercial Griess reagent contains 0.2% naphthylenediamine dihydrochloride and 2% sulfanilamide in 5% phosphoric acid. The Griess reagent is used for photometric detection of nitrite. It consists of two key components: sulfanilic acid and N-(1-naphthyl)ethylenediamine. Under acidic conditions, sulfanilic acid reacts with nitrite to form a diazonium salt, which readily couples with N-(1-naphthyl)ethylenediamine, creating a highly colored azo dye detectable at 548 nm (Fig. 42). Under physiological conditions, NO is unstable and rapidly oxidized into a mixture of nitrite and nitrate. To indirectly measure NO levels from nitrite, nitrate is enzymatically reduced to nitrite using nitrate reductase, enabling the total nitrite amount to be determined (Gülçin 2012).

Additionally, the nitrosating agent dinitrogen trioxide (N_2_O_3_), generated either through the autoxidation of nitric oxide (NO) or the acidification of nitrite (NO_2_), reacts with sulfanilamide to produce a diazonium derivative. This reactive intermediate then interacts with N-(1-naphthyl)ethylenediamine to form a colored diazo product that exhibits strong absorption at 548 nm (Tarpey et al. 2004). When compared to other methods, this technique is more complex, requiring the addition of multiple enzymatic reagents. The fluorescent probe 4,5-diaminofluorescein, commonly used for in vivo detection and imaging of NO, has also been adapted for assessing NO· scavenging capacity (Nagata et al. 1999). Nitrosation of the weakly fluorescent 4,5-diaminofluorescein by NO· derivatives leads to the formation of a highly fluorescent green triazolofluorescein product. The NO· scavenging capacity is evaluated based on the ability of test compounds to inhibit NO-induced nitration of 4,5-diaminofluorescein. Results are expressed as the percentage inhibition of 4,5-diaminofluorescein oxidation relative to the concentration of the NO· scavenging compound. However, caution is advised when interpreting results obtained using 4,5-diaminofluorescein as a target for NO· quantification. Certain antioxidant compounds, such as ascorbic acid and dehydroascorbic acid, can directly react with 4,5-diaminofluorescein, forming fluorescent products with emission spectra similar to the green-fluorescent triazolofluorescein product (Magalhaes et al. 2008).

### Peroxynitrite radical (ONOO·) scavenging assays

Peroxynitrite (ONOO⁻) is a key reactive oxidant and nitrating agent known to contribute to neuronal cell damage and has been implicated in the pathogenesis of neurodegenerative diseases (Chen et al. 2012). Peroxynitrite anion (ONOO⁻) is a highly reactive, short-lived oxidant with a half-life of approximately 1 s under physiological conditions. It is highly permeable to cell membranes (Schieke et al. 1999). Formed by the bi-radical reaction between nitric oxide (NO) and superoxide (O₂^•^⁻) at a diffusion-limited rate, ONOO⁻ is a potent oxidizing and nitrating species. The peroxynitrite radical (ONOO•) exerts cytotoxic effects through strong oxidative reactions with cellular components, including lipids, thiols, amino acids, and nucleotides, leading to outcomes such as lipid peroxidation, protein oxidation, carcinogenesis, and aging. These radicals are generated in vivo by endothelial cells, neutrophils, and macrophages. Although peroxynitrite is relatively stable compared to other free radicals, once protonated, it forms highly reactive peroxynitrous acid (ONOOH). This acid decomposes rapidly with a short half-life of 1.9 s at 37 ℃, producing various cytotoxic agents. Peroxynitrite can induce lipid peroxidation, oxidation of protein thiol (-SH) groups, tyrosine nitration, and nitrosation reactions, ultimately disrupting cell metabolism and signal transduction. This can result in tissue and cellular damage, DNA strand breaks, and apoptotic cell death. Excessive production of ONOO⁻ has been linked to several human diseases, including Alzheimer’s disease, cancer, rheumatoid arthritis, and atherosclerosis. Due to the absence of endogenous enzymes that specifically inactivate ONOO•, the development of targeted peroxynitrite scavengers is crucial.

A described method for assessing ONOO⁻ scavenging involves using a stock solution of dihydroxyrhodamine (5 mM) prepared in dimethylformamide, purged with nitrogen, and stored at − 80 °C. Before the experiment, a working solution of dihydroxyrhodamine (5 μM) is diluted from the stock, kept on ice, and protected from light. The phosphate buffer solution (50 mM, pH 7.4) containing NaCl (90 mM), KCl (5 mM), and diethylenetriaminepentaacetic acid (0.1 mM) is purged with nitrogen and kept on ice before use. The scavenging activity of ONOO radicals is determined by measuring the oxidation of dihydroxyrhodamine using a microplate fluorescence spectrophotometer. The fluorescence is recorded at excitation and emission wavelengths of 485 nm and 530 nm, respectively, at room temperature. Background and final fluorescence intensities are measured 5 min after treatment, in the absence of 3-morpholinosydnonimine or authentic ONOO⁻. While the oxidation of dihydroxyrhodamine by 3-morpholinosydnonimine decomposes gradually, authentic ONOO⁻ oxidizes dihydroxyrhodamine rapidly, with the resulting fluorescence intensity stabilizing over time (Alam et al. 2013a, b).

### Nonradical reactive oxygen species scavenging Assay

#### Hydrogen peroxide (H_2_O_2_) scavenging

Humans are exposed to hydrogen peroxide (H₂O₂) indirectly through the environment at an estimated rate of approximately 0.28 mg/kg per day, with the primary source being leafy vegetables. H₂O₂ can enter the human body through inhalation of its vapor or mist, as well as via contact with the eyes or skin. Once inside the body, it rapidly decomposes into molecular oxygen and water, which can lead to the production of hydroxyl radicals (OH·). These radicals are highly reactive and can initiate lipid peroxidation and cause DNA damage. H₂O₂ is a biologically significant, non-radical oxidizing agent, naturally formed in tissues as a by-product of oxidative processes. It is also produced in organisms during oxygen metabolism. The reduction of O₂ by electrons initially forms superoxide anion (O₂^•^⁻), which is then either spontaneously or enzymatically converted to H₂O₂. This compound can degrade biological macromolecules such as proteins, enzymes, lipids, carbohydrates, and nucleic acids by generating OH· radicals. In the presence of transition metal ions, H₂O₂ is readily converted to OH· through the Fenton (1984) or Haber–Weiss (1934) reactions. Almost all living organisms possess peroxidase enzymes that catalytically and safely decompose low concentrations of H_2_O_2_ into water and oxygen (Gülçin et al. 2003c). In physiological conditions, H₂O₂ is generated in vivo by peroxisomes, through the activity of various oxidative enzymes such as glucose oxidase and D-amino acid oxidase, or via the dismutation of superoxide radicals catalyzed by superoxide dismutase. Although H₂O₂ can easily cross cellular membranes, it is relatively unreactive at low concentrations and reacts slowly with most compounds. However, it plays a crucial role in the respiratory burst of activated phagocytes and is produced by several oxidase enzymes within the body.

Hydrogen peroxide (H_2_O_2_) is also produced from polyphenol-rich beverages under quasi-physiological conditions, with its concentration increasing over longer incubation periods. The H_2_O_2_ generated by activated phagocytes plays a key role in the destruction of various bacterial and fungal strains. It is widely recognized as a powerful oxidizing agent, and there is growing evidence that H_2_O_2_, either directly or indirectly via its reduction product (OH·), functions as a signaling molecule in the synthesis and activation of inflammatory mediators. H_2_O_2_ can cross cell membranes and gradually oxidize several compounds. It is also utilized during the respiratory burst of activated phagocytes (Gülçin et al. 2003c). One of the most common methods to evaluate the scavenging activity against H_2_O_2_ involves its intrinsic absorption in the UV spectrum at 230 nm (Santocono et al. 2006; Berges et al. 2007). Measuring H_2_O_2_ scavenging activity in food and biological fluids is critical, and various assays have been developed for this purpose. These methods often rely on the oxidation of spectrophotometric, fluorogenic, or chemiluminogenic probes by H_2_O_2_, with horseradish peroxidase or transition metal ions serving as oxidation catalysts (Apak et al. 2022).

A standard assay to assess H_2_O_2_ scavenging capacity uses a solution of H_2_O_2_ (40 mM) prepared in phosphate buffer (50 mM, pH 7.4). The remaining H_2_O_2_ concentration is determined spectrophotometrically by measuring absorption at 230 nm after 10 min of incubation, compared to a blank containing only the phosphate buffer (Gülçin et al. 2003c). As H₂O₂ is scavenged by tested compounds, the absorbance at 230 nm decreases. However, this method has limitations. For example, some samples also absorb light at this wavelength, necessitating a “blank” measurement, which can compromise the method’s precision and accuracy (Magalhães et al. 2008). Additionally, distinguishing small absorbance changes against a significant background can be challenging. Moreover, the interaction between samples and H₂O₂ may alter absorption properties, rendering the blank measurement invalid. Another commonly used assay employs horseradish peroxidase, which uses H₂O₂ to oxidize scopoletin into a non-fluorescent product. The presence of H_2_O_2_-decomposing compounds inhibits this oxidation, allowing the reaction to be monitored fluorometrically (Corbett 1989). It is also well-established that H_2_O_2_ is toxic and induces cell death in vitro. It can attack cellular energy production systems, such as deactivating the glycolytic enzyme glyceraldehyde-3-phosphate dehydrogenase (Ak and Gülçin 2008). Although H_2_O_2_ itself is not highly reactive, its toxicity arises from its conversion into hydroxyl radicals (HO·) within cells, a process that can cause oxidative DNA damage in a transition metal ion-dependent manner. In cultured cells, H_2_O_2_ concentrations of 20–50 μg/cell exhibit limited cytotoxicity to many cell types. However, it is widely believed that H_2_O_2_ is highly toxic in vivo and must be rapidly eliminated by enzymes like catalases, peroxidases, and thioredoxin-linked systems (Halliwell et al. 2000). Thus, removing H_2_O_2_, along with superoxide anions, is crucial for protecting pharmaceutical products and food systems (Chai et al. 2003). The primary danger of H₂O₂ lies in its ready conversion to the highly reactive HO· radical, either through ultraviolet light exposure or interactions with transition metal ions, particularly iron (Halliwell et al. 2000).

#### Singlet oxygen (^1^O_2_) quenching assays

Singlet oxygen (^1^O₂) acts as a more selective oxidant compared to other ROS, primarily targeting double bonds to form endoperoxides. These can further reduce to alkoxyl radicals, initiating radical chain reactions (Prior et al. 2005). As an excited state of molecular oxygen, singlet oxygen is a non-radical oxidant with no unpaired electrons, making it highly reactive with biomolecules. Its decay to the ground state emits characteristic phosphorescence at 1270 nm, utilized for measuring ^1^O₂ scavenging activity (Wilkinson et al. 1995). The intensity of self-emission luminescence of ^1^O_2_ often lacks reproducibility. A sensitive method involving fluorescence quenching of tetratert-butylphthalocyanine was developed for better quantification. Additionally, fluorescence-based microplate screening assays using thermal decomposition of endoperoxides have been employed for evaluating ^1^O_2_ scavenging activity (Fu et al. 1997).

Singlet oxygen is implicated in UV-induced skin damage, cataract formation, macular degeneration, and photosensitivity from certain phytochemicals and pharmaceuticals (Zigman 2000). It’s in vivo formation, independent of light, can result from O₂^•−^ dismutation or the non-photochemical decomposition of H_2_O_2_ via metals or hypochlorite (MacDonald-Wicks et al. 2006; Huang et al. 2005). This assay has been applied to known ^1^O_2_ quenchers like β-carotene, α-tocopherol, and lauric acid, measuring the quenching rate constants with commonly available equipment (Huang et al. 2005).

#### Metal chelating assay

Metal ions are essential for maintaining the vital functions of living organisms. However, for thousands of years, humans have extensively used metals for everyday purposes without fully considering their negative impacts and consequences. As a result, metal ions have not only disrupted entire ecosystems but also polluted water resources, significantly harming plant and animal life. Today, the primary sources of metal pollution include mining activities, industrial wastewater, urban waste, acid rain, fossil fuel residues, fertilizers, and pesticides. Heavy metals are generally defined as elements with a density greater than 5 g/cm^3^. These elements are found in the periodic table and often have high atomic weights (Duffus 2002). Examples include lead (Pb), cadmium (Cd), mercury (Hg), and arsenic (As), among others. While some heavy metals are essential in trace amounts for biological processes (e.g., zinc, iron, and copper), others are toxic even at low concentrations (e.g., Cd, Hg, and Pb). Their toxicity often stems from their ability to bioaccumulate and disrupt cellular functions by binding to proteins and enzymes. Heavy metals are widely studied in environmental science, toxicology, and industrial contexts due to their persistence, non-biodegradability, and potential harm to ecosystems and human health (Tchounwou et al. 2012).

Iron (Fe) serves as a prime example of metals to which we are frequently exposed or consume excessively in daily life. The average human body contains approximately 4–5 g of elemental iron. Two-thirds of this iron is found in hemoglobin, the oxygen-transporting protein, while the remaining one-third is stored in iron-binding proteins such as hemosiderin and ferritin (Vacca et al. 2004). Iron also plays a critical role in cytochromes, hemoglobin, myoglobin, and is essential for the function of various enzymes, including peroxidases, catalase, succinate dehydrogenase, aconitase, aldehyde oxidase, and oxygenase (Beard 2008; Kim et al. 2019). Despite the human body's ability to tolerate relatively high levels of iron, excessive iron can be highly toxic. Cases of metal poisoning have become increasingly common in young children due to the overconsumption of iron-fortified supplements. While acute iron poisoning is rare in adults, chronic iron overload is frequently observed in patients with β-thalassemia as a result of the regular blood transfusions they require (Harris et al. 1981; Vacca et al. 2004). Excessive iron accumulation primarily impacts the heart and liver. The regular elimination of excess metal ions can be enhanced by using an appropriate chelating agent (Gülçin and Alwasel 2022).

Metal chelation capacity is evaluated by assessing the ability of antioxidants to chelate metal ions, such as Fe^2^⁺, which an elemental species, plays a role in promoting ROS generation within animal and human systems. Substances capable of chelating Fe^2^⁺ demonstrate significant antioxidant potential. While iron is an essential mineral for normal physiological functions, its excess can lead to cellular damage. Among transition metals, iron is considered the most critical pro-oxidant in lipid oxidation due to its high reactivity (Gulcin 2012). Transition metal ions stimulate lipid peroxidation both through the Fenton reaction and by breaking down lipid hydroperoxides into more reactive peroxyl and alkoxyl radicals.

Iron is one of the most important elements for most forms of life and plays a significant role in various physiologically important functions, owing to its distinctive redox properties (Bou-Abdallah 2010). However, the high reactivity and interconversion between the multiple oxidation states of iron can be problematic and can cause cellular damage and apoptosis. One of the most harmful reactions in biology is the Fenton reaction, where Fe^2+^ ions catalyze the generation of highly toxic reactive chemical species that can lead to pathological processes, including cancer and neurodegenerative diseases (Li and Reichmann 2016). Many chemical tools and assays have been developed to qualitatively and quantitatively measure the redox status of iron and its distribution and accumulation in biological systems (Hirayama and Nagasawa 2017). One of the most common and frequently used spectroscopic methods to quantify the concentration of metal ions in aqueous solutions involves the measurement of absorbance changes using chromogenic reagents (Smith et al. 2021).

These metal ions-ligand complexes show unique absorption spectra with characteristic colors in the visible region of the electromagnetic spectrum. Effective Fe^2^⁺ chelators can mitigate oxidative damage by sequestering iron, thereby preventing its participation in hydroxyl radical (HO·) production through Fenton-type reactions. Reduced metals involved in Fenton reactions contribute to oxidative stress by forming highly reactive hydroxyl radicals. While Fe^3^⁺ can also generate radicals from peroxides, the reaction rate is approximately ten times slower than that of Fe^2^⁺ (Kehrer 2000)

$${\text{Fe}}^{2 + } + {\text{H}}_{2} {\text{O}}_{2} \to {\text{ Fe}}^{3 + } + {\text{ OH}}^{ - } + {\text{OH}}^{\cdot}$$

The term “Fenton reagent” refers to a mixture of H₂O₂ and ferrous salts, an effective oxidant for a wide variety of organic substrates. Discovered in 1894 by Fenton, this reagent oxidizes tartaric acid to dihydroxymaleic acid in the presence of low concentrations of ferrous salts and H_2_O_2_. Later in 1934, Haber and Weiss suggested that hydroxyl radicals (OH·) are active intermediates formed during the iron salt-catalyzed decomposition of H_2_O_2_. O₂·⁻ can also be detoxified into H₂O₂ through the dismutation reaction catalyzed by SOD and subsequently broken down to water by CAT. When H₂O₂ reacts with Fe^2^⁺, the Fenton reaction produces hydroxyl radicals (Carocho and Ferreira 2013).

Hydroxyl radicals generated by the Fenton reagent are efficient hydroxylating agents. Numerous metal ions in lower oxidation states, such as Cu⁺, Ti^3^⁺, Cr^2^⁺, and Co^2^⁺, also react with H_2_O_2_ in a manner similar to Fe^2^⁺. These combinations with H_2_O_2_ are termed “Fenton-like” reagents (Ou et al. 2002a, b). Unlike outer-sphere electron transfer, the reaction between transition metals and H_2_O_2_ occurs via an inner-sphere mechanism, forming a complex before electron transfer (Goldstein et al. 1993). Transition metals, when coordinately saturated, cannot react with H₂O₂ to produce ROS. Halliwell et al. demonstrated that iron chelators inhibit the Fe^2^⁺-mediated Fenton reaction, thereby preventing oxidative damage (Ou et al. 2002a, b).

Iron is one of the essential elements in the body, and its concentration is tightly regulated. Iron overload, characterized by iron deposition in multiple organs, is associated with a serum ferritin level exceeding 1000 µg/L (Angelucci 2015). Iron, an essential mineral for biological functions, becomes toxic as free ions due to its role in hydroxyl radical formation via the Fenton reaction. Ferrous ions (Fe^2^⁺) are potent lipid oxidation pro-oxidants, stimulating ROS production and damaging lipids, proteins, and nucleic acids (Halliwell and Gutteridge 1984). Effective Fe^2^⁺ chelators protect against oxidative stress by reducing ROS formation. Ferric ions (Fe^3^⁺), though less reactive, also produce radicals from peroxides. In food systems, chelating ferrous ions reduces oxidative damage, offering significant antioxidative benefits (Gülçin 2012). Iron is stored in cells via proteins like ferritin, which can sequester up to 4500 iron atoms per molecule as a ferrihydrite mineral core, protecting cells from the toxic effects of free iron ions (Arosio et al. 2009a, b). Ferritin level changes paralleled with liver iron concentration variations. In particular, ferritin levels above 2500 ng/mL are associated with an increased risk of morbidity and mortality and should be trigger intensification of chelation therapy (Ricchi et al. 2010). It was reported that the 71% mortality rate in cardiac disease due to iron accumulation in the myocardium is a significant complication of iron overload in beta-thalassemia (Telfer et al. 2000). The aim of chelation therapy is to prevent the accumulation of excess iron and its complications, such as hepatic, endocrinological, and cardiac dysfunction (Junqueira et al. 2013). Excessive iron levels are associated with diseases such as vascular conditions, cancer, and neurological disorders, often linked to iron-mediated ROS formation and DNA damage (Valko et al. 2007).

**Fig. 43:**
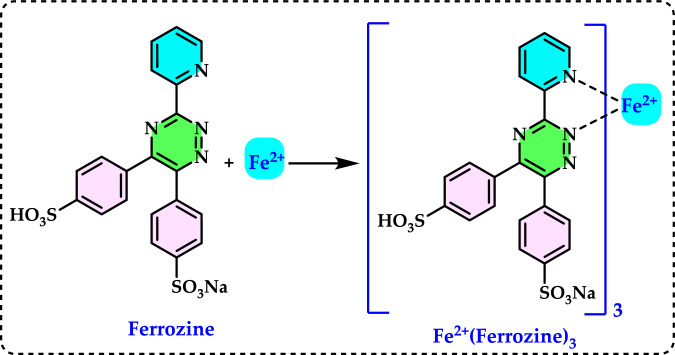
The chemical structure of ferrozine and Ferrozine-Fe^2+^ complex

Metal chelation is the process by which metal ions, often transition metals like Fe^2^⁺, Cu^2^⁺, or Zn^2^⁺, are bound by specific molecules called chelating agents to form stable complexes. Chelating agents typically have multiple donor atoms such as oxygen, nitrogen, or sulfur that form coordinate bonds with the metal ion. These agents can prevent metals from participating in chemical reactions that might otherwise lead to harmful effects, such as oxidative damage (Andjelkovic et al. 2006). Chelating agents often have functional groups like –OH, –COOH, –SH, –NH_2_, or C=O, which can donate electron pairs to the metal ion. Chelators like transferrin and ferritin in the body regulate iron levels (Gülçin 2020). One of the most common and frequently used ferrous iron chelators is ferrozine, which has been extensively used in a variety of applications to quantify ferrous ions. One of the main advantages of ferrozine over other reagents is its high water solubility, high sensitivity, and stability over a wide range of pH. The ferrous-ferrozine complex exists in a 3:1 ratio of ferrozine to iron and is reported to have one of the highest molar absorptivity values (i.e., 27,900 M^−1^ cm^−1^) at 562 nm among other commonly used iron complexes (pH 4 to 9) (Stookey 1970). Ferrozine has one pyridyl group and two phenylsulfonate groups on a 1,2,4-triazine core. The two sulfonate groups provide high water-solubility characteristics and allow utilization in aqueous samples. The ferrozine assay has been widely used in the detection of ferrous ions in a variety of samples, including mineral and biological systems. The accurate quantification of Fe^2+^ ions is affected by several factors, including pH, temperature, concentration of reagents, incubation time, anions such as oxalate, cyanide, and nitrite, and also the presence of Fe^3+^ cations (Smith et al. 2021). Due to the relatively high binding affinity of ferrozine for Fe^2+^ ions, many studies have used stoichiometric amounts of ferrozine, or a slight excess of chelator, to ensure that Fe^2+^ ions are fully bound. Surprisingly, while investigating the reductive mobilization of iron from the major iron storage protein ferritin, we obtained conflicting results and anomalous absorbance values that were inconsistent with literature reports and the published molar absorptivity for the Fe^2+^-(Ferrozine)_3_ complex (Fig. 43) (Thompsen and Mottola 1984; Smith et al. 2021).

$$3\text{Ferrozine }+{\text{Fe}( {\text{H}}_{2}\text{O})}_{6}^{2+} \to {\text{Fe}-(\text{Ferrozine})}_{3}^{4-} + {6\text{H}}_{2}\text{O}$$

A key limitation of this complexation reaction is its dependency on the relative binding constants of the Antioxidant-Fe^2^⁺ and ferrozine-Fe^2^⁺ complexes, as well as the competition between the two chelators for iron binding (Taslimi et al. 2017; Sarı et al. 2018). This can lead to a significant underestimation of weak antioxidant iron chelators during quantitative measurements. From a nutritional standpoint, the role of weak antioxidant iron chelators in preventing the Fenton reaction in vivo remains unclear. Despite this, the assay provides a convenient method for evaluating the iron-chelating activity of antioxidants. The metal chelating capacity was significant as it reduced the concentration of the catalyzing transition metal in lipid peroxidation. It was reported that chelating agents are effective as secondary antioxidants because they reduce the redox potential, thereby stabilizing the oxidized form of the metal ions (Gulcin 2012).

The chelating capacity of ferrous ions can be measured using the “ferrozine method”, where ferrozine forms a red complex with Fe^2^⁺. Chelating agents disrupt this reaction, reducing the red color intensity. The degree of color reduction quantifies the metal chelating activity, with lower absorbance indicating stronger chelation. This method can detect iron concentrations at nanomolar levels, and it is widely used to evaluate antioxidant activity in conjunction with other assays (King et al. 1991a, b; Gülçin et al. 2003c). Chelating agents can mitigate oxidative stress-induced diseases by reducing free iron ion levels and preventing ROS generation. Chelation's ability to inhibit Fe^2^⁺-mediated lipid peroxidation and other oxidative processes highlights its therapeutic and preservative potential in biomedical and food sciences.

Ferrous sulfate (FeSO_4_) and ferrous chloride (FeCl₂) are among the most commonly used sources of ferrous ions. The decrease in absorbance at 485 nm (for 2,2′-bipyridine) or 562 nm (for ferrozine) following the addition of antioxidants indicates the formation of a metal-antioxidant complex. This change allows the metal chelation capacity of the antioxidant to be quantified using spectrophotometric analysis (Sujayev et al. 2016; Turan et al. 2016). One method for evaluating the metal-chelating activity of an antioxidant involves measuring the absorbance of the Fe^2^⁺-ferrozine complex after pre-treatment of a ferrous ion solution with the test material. Ferrozine specifically forms a complex with free Fe^2^⁺, but not with Fe^2^⁺ already bound to other chelators. A reduction in the amount of ferrozine-Fe^2^⁺ complex formed following antioxidant treatment indicates the presence of antioxidant chelators. The ferrozine-Fe^2^⁺ complex produces a red chromophore with absorbance measurable at 562 nm (Gulçin and Alwasel 2022).

**Fig. 44:**
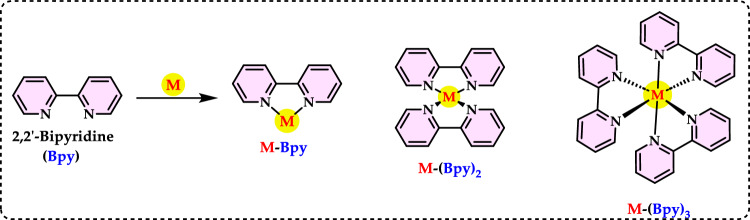
The coordination behaviors of 2,2'-bipyridine (Bpy) and metal

2,2′-Bipyridine (Bpy) units can in principle be used as bridges to interconnect metal centers in a well-defined spatial arrangement. Bpy is the unique molecular scaffold of the bioactive natural products. Bpy is extensively used as the core structure of many chelating ligands by acting as a bridge in the arrangement of the catalytic center. Bpy shows robust redox stability and hyperglycemic activity (Gulçin and Alwasel 2022). Transition metal complexes of Bpy are coordination complexes containing one or more Bpy ligands. Complexes have been described for all of the transition metals. Bipyridine complexes absorb intensely in the visible part of the spectrum. In the M(Bpy) catalyses, catalytically active species are likely to be in the form of Bpy-monochelated M-complexes M(Bpy), but dynamic coordination equilibria per-turb the number of Bpy ligands on the M atom (Fig. 44). These coordination behaviors may lead to a decrease in catalytic efficiency (Kawa-mata et al. 2019). Analysis of metal coordination behaviors of the Bpy ligands by UV–Vis absorption spectroscopy indicated the apparent monochelating nature of the dumbbell-shaped Bpy ligands (Kim et al. 2021).

**Fig. 45:**
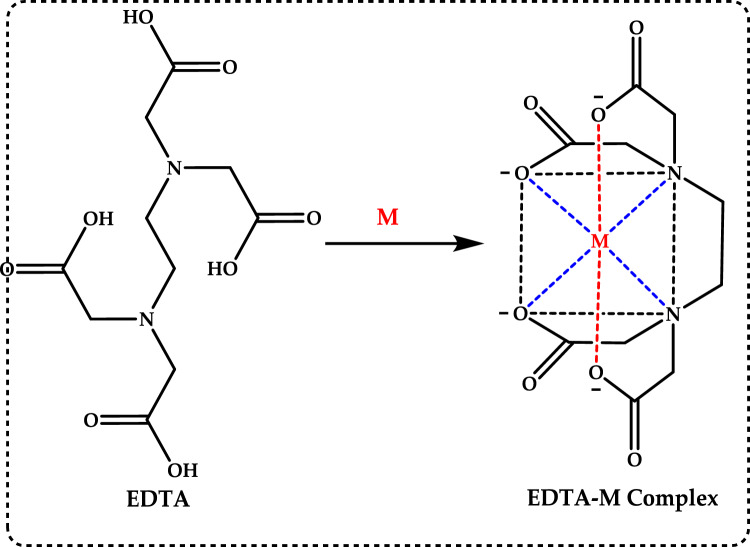
The formation of ethylenediaminetetraacetic acid (EDTA) and metal complex (EDTA-M)

Ethylenediaminetetraacetic acid (EDTA) is widely used as a standard metal chelator in food and pharmaceutical applications. The metal chelation activity of antioxidant compounds or plant extracts is often expressed in terms of EDTA equivalents (Ozbey et al. 2016). EDTA is a hexadentate chelating agent, commercially available as its disodium salt. The formation of a chelate complex with EDTA depends on the pH of the medium, as each metal ion forms a complex with the chelating agent at a specific pH. Due to its hexadentate nature, EDTA can bind only one metal ion per molecule (Fig. 45) (King Lin and Kester 1991). In medicine, derivatives of EDTA are used for chelation therapy to bind and remove harmful metal ions. The calcium sodium derivative of EDTA, administered intravenously, has been employed for treating mercury and lead poisoning. It is also effective in eliminating excess iron from the body. The FDA has approved EDTA for treating lead or mercury poisoning. Additionally, EDTA is used as an anticoagulant in blood samples. The most common side effect of EDTA is renal toxicity, which should be carefully monitored during its use.

**Fig. 46:**
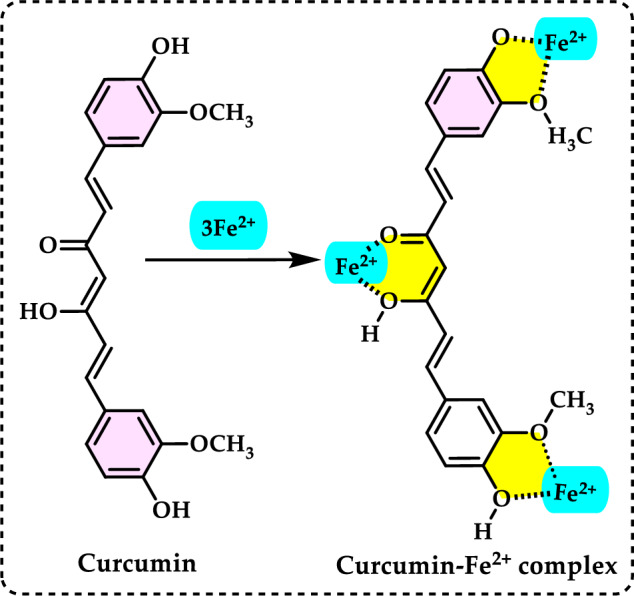
The suggested mechanism for ferrous ion (Fe^2^⁺) chelation by curcumin

It was reported that food constituents have a marked capacity for iron binding, suggesting that their main action as peroxidation inhibitors may be related to their iron-binding capacity (Ak and Gülçin 2008; Gülçin 2010). In this assay, curcumin, as a food constituent, interfered with the formation of the ferrous-ferrozine complex, showing chelating activity and the ability to capture ferrous ions with a higher binding affinity than ferrozine. For example, as depicted in Fig. 46, curcumin may chelate the ferrous ion with its -OH and -OCH_3_ groups. It was reported that compounds with structures containing C–OH and C=O functional groups can chelate metal ions. Kazazica and co-workers (2006) demonstrated that flavonoids, such as kaempferol, chelated Cu^2+^ and Fe^2+^ through the functional carbonyl groups. Compounds with structures containing two or more of the following functional groups: –OH, –SH, –COOH, –PO_3_H_2_, C=O, -NR_2_, –S– and –O– in a favorable structure–function configuration can exhibit metal chelation activity (Yuan et al. 2005; Gulcin 2006a). The structure of curcumin and its binding sites for metal chelation are given in Fig. 46. Recently, Fiorucci and co-workers (2007) demonstrated that quercetin chelated metal ions in the same way.

**Fig. 47:**
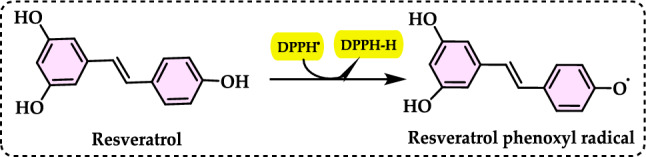
Two resveratrol molecules chelate activity one ferrous ion before ferrozine

In a different study, it was demonstrated that L-Carnitine chelates Fe^2^⁺ through its carbonyl and hydroxyl functional groups. Similarly, curcumin was shown to bind Fe^2^⁺ via its carbonyl and hydroxyl groups (Ak and Gülçin 2008). Likewise, L-Adrenaline binds Fe^2^⁺ through its amine and hydroxyl groups (Gülçin, 2009). In a comparable manner, two molecules of resveratrol bind Fe^2^⁺ through their hydroxyl groups. Resveratrol exhibits a strong iron-binding capacity, suggesting that its primary function as a peroxidation inhibitor is likely linked to this property. In this assay, resveratrol disrupts the formation of the ferrous-ferrozine complex, indicating its chelating activity and ability to capture ferrous ions prior to ferrozine. The structure of resveratrol and its binding sites for metal chelation are depicted in Fig. 47.

**Fig. 48:**
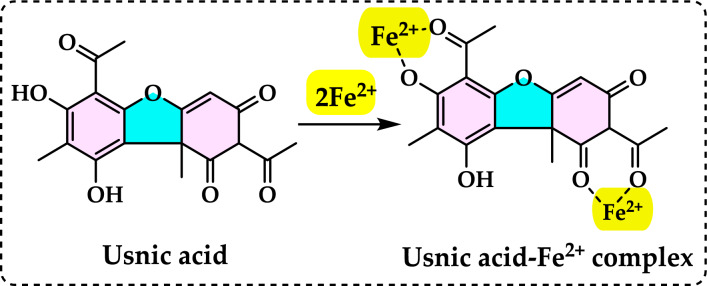
Possible ferrous ions (Fe^2+^) chelating mechanism of usnic acid

A recent study explored the potential Fe^2^⁺ chelating mechanism of usnic acid. The findings revealed that usnic acid disrupted the formation of the ferrozine-Fe^2^⁺ complex. Acting as a metal-binding agent, usnic acid exhibited Fe^2^⁺ chelation activity, effectively binding Fe^2^⁺ ions before ferrozine. Figure 48 illustrates the structure of usnic acid along with its binding sites for metal chelation, indicating that it may chelate Fe^2^⁺ through hydroxyl and carboxyl groups attached to its phenolic ring (Çetin Çakmak and Gülçin, 2019).

### Thiobarbituric acid value (TBA)

The thiobarbituric acid reactive substances (TBARS) assay is widely utilized for evaluating and monitoring lipid peroxidation due to its simplicity and cost-effectiveness. In this method, MDA, an advanced byproduct of unsaturated lipid degradation, reacts with thiobarbituric acid (TBA) under acidic conditions. This reaction produces a distinct chromogenic compound, [MDA-(TBA)₂], which forms at 100 °C temperatures and is measured spectrophotometrically at 532 nm (Gülçin, 2012). MDA is a naturally occurring organic compound and a recognized marker of oxidative stress. ROS degrade polyunsaturated lipids, generating MDA (Pryor and Stanley 1975).

**Fig. 49:**
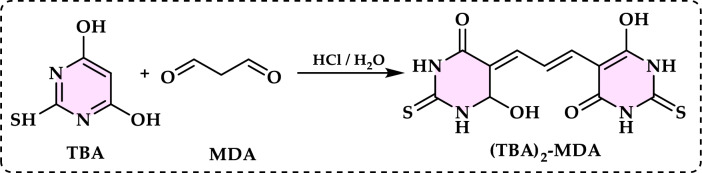
Formation of condensation reaction between thiobarbituric acid (TBA) and malondialdehyde (MDA)

MDA, a reactive aldehyde, belongs to a class of reactive electrophile species that induce toxic stress in cells and form covalent protein adducts known as advanced lipoxidation end products, similar to advanced glycation end products. This aldehyde serves as a biomarker for assessing oxidative stress levels in organisms (Farmer and Davoine 2007). MDA can be derived from polyunsaturated fatty acids with three or more double bonds. Its concentration is typically evaluated by reacting it with TBA, forming red condensation products (Fig. 49) that absorb light at 532–535 nm, with a molar absorptivity of 27.5 absorbance units/mmol. MDA and other TBARS react with two equivalents of TBA, resulting in a fluorescent red derivative detectable spectrophotometrically (Nair, O'Neil, and Wang, 2008). The MDA equivalents (μmol) in samples are determined using the molar extinction coefficient of the chromogenic product (1.56 × 10^5^ M⁻^1^ cm⁻^1^). The TBARS assay quantifies the MDA generated from lipid peroxidation; however, other aldehydes produced during the process may also react with TBA and absorb at 532 nm. Consequently, this assay is not entirely specific for lipid peroxidation products. TBA interacts with various aldehydes, not exclusively those arising from lipid peroxidation (Apak et al. 2022).

The reaction, however, is not specific, as it can interact with a wide variety of other compounds, potentially influencing the absorbance measurement. For instance, 2,4-alkadienals such as 2,4-decadienal also react with TBA, exhibiting strong absorption at 532 nm, while saturated aldehydes typically absorb at lower wavelengths after reacting with TBA. Additionally, various food components, including proteins, Maillard reaction products, and sugar degradation products, can interfere with the determination process. To highlight this lack of specificity, the results of the test are generally referred to as TBARS or TBA-reactive substances. A detailed review of the TBA test was conducted by Guillen-Sans and Guzman-Chozas in 1998. MDA has been shown to react with deoxyadenosine and deoxyguanosine in DNA, leading to the formation of DNA adducts, with M1G being the primary and mutagenic product. Additionally, the guanidine group in arginine residues condenses with MDA to produce 2-aminopyrimidines (Marnett 1999). The TBARS method has limitations, as it cannot distinguish between the stoichiometry of the oxidation reaction and its kinetics. Furthermore, the oxidation of TBA by substances unrelated to lipid peroxidation and the formation of Schiff bases between MDA and amines can lead to inaccurate estimates of antioxidant protection (Spickett et al. 2010; Apak et al. 2022).

## Conclusion

Oxygen metabolism leads to the formation of various radical and non-radical oxidants, each with its own distinct properties. The chemical reactivity of these molecules varies, and their targets in a physiological environment are determined by kinetic factors. In some cases, biological mechanisms can be easily explained by the known reactions of the involved species. However, in other cases, when mechanisms do not align with the chemistry, more detailed analysis is required. The core principle of antioxidant activity is centered on the availability of electrons to neutralize free radicals. Additionally, antioxidant activity is influenced by the quantity and arrangement of hydroxyl groups on the aromatic ring. It is widely believed that the ability to donate hydrogen and prevent oxidation improves as the number of hydroxyl groups on the phenolic ring increases. Phenolic compounds represent a highly diverse class of phytochemicals, which are abundantly present in plants such as fruits, vegetables, tea, olive oil, and tobacco. In recent times, there has been increasing interest in substances with antioxidant properties, either as components of food or as targeted preventive pharmaceuticals. As a result, antioxidants have become indispensable in food preservation technology and modern healthcare. Plants with antioxidative and pharmacological attributes are widely recognized for their phenolic compounds, particularly phenolic acids and flavonoids. The effective discovery of natural antioxidant sources requires reliable methods for evaluating antioxidant activity. Currently, numerous bioanalytical techniques are available to determine the antioxidant capacity of food components, as detailed in this study. These assays vary significantly in terms of reaction mechanisms, substrates, oxidants and target species, reaction conditions, oxidation initiators, data presentation, and ease of execution. They also differ mechanistically. Total antioxidant capacity depends on numerous factors, and understanding the behavior of antioxidants is essential to develop a comprehensive antioxidant profile. However, comparing data across different studies is often challenging. Thus, when selecting a method, it is critical to prioritize the reaction mechanism. For determining antioxidant capacity via in vitro assays, it is important to consider factors such as sources of oxidation, concentrations, interactions with other oxidants, biological targets, relevance to oxidative stress, and the surrounding environment. Selecting the appropriate method or a combination of methods is crucial for accurately assessing antioxidant activity and for evaluating the potential applications of antioxidants in food preservation, pharmaceuticals, or as health-enhancing agents.
